# Fractionation, Stability, and Isolate-Specificity of QTL for Resistance to *Phytophthora infestans* in Cultivated Tomato (*Solanum lycopersicum*)

**DOI:** 10.1534/g3.112.003459

**Published:** 2012-10-01

**Authors:** Emily B. Johnson, J. Erron Haggard, Dina A. St.Clair

**Affiliations:** Department of Plant Sciences, University of California, Davis, California 95616

## Abstract

Cultivated tomato (*Solanum lycopersicum*) is susceptible to late blight, a major disease caused by *Phytophthora infestans*, but quantitative resistance exists in the wild tomato species *S. habrochaites*. Previously, we mapped several quantitative trait loci (QTL) from *S. habrochaites* and then introgressed each individually into *S. lycopersicum*. Near-isogenic lines (NILs) were developed, each containing a single introgressed QTL on chromosome 5 or 11. NILs were used to create two recombinant sub-NIL populations, one for each target chromosome region, for higher-resolution mapping. The sub-NIL populations were evaluated for foliar and stem resistance to *P. infestans* in replicated field experiments over two years, and in replicated growth chamber experiments for resistance to three California isolates. Each of the original single QTL on chromosomes 5 and 11 fractionated into between two and six QTL for both foliar and stem resistance, indicating a complex genetic architecture. The majority of QTL from the field experiments were detected in multiple locations or years, and two of the seven QTL detected in growth chambers were co-located with QTL detected in field experiments, indicating stability of some QTL across environments. QTL that confer foliar and stem resistance frequently co-localized, suggesting that pleiotropy and/or tightly linked genes control the trait phenotypes. Other QTL exhibited isolate-specificity and QTL × environment interactions. Map-based comparisons between QTL mapped in this study and Solanaceae resistance genes/QTL detected in other published studies revealed multiple cases of co-location, suggesting conservation of gene function.

Late blight is a disease of cultivated tomato (*Solanum lycopersicum*) and its close relative potato (*Solanum tuberosum*) that is caused by the oomycete *Phytophthora infestans*. Infection causes lesions on the leaves and stems and facilitates secondary infection, causing the tomato fruit or potato tubers to rot and become unmarketable (Fry and Goodwin 1997). *P. infestans* is a highly aggressive pathogen that spreads rapidly and can cause complete defoliation and death of the host within 1-2 weeks of the first symptoms (Fry and Goodwin 1997), and it causes significant crop losses in many parts of the world. In the US, late blight causes an estimated $5 billion per year in disease control costs and crop losses to potato and tomato growers (Judelson and Blanco 2005). For disease control, growers employ crop rotations, frequent fungicide applications, and tolerant varieties when available.

Tomato is a major vegetable crop in many parts of the world, and the second most valuable vegetable in US production (National Agriculture Statistics Service 2011). Currently utilized commercial tomato cultivars are not resistant to *P. infestans*. Several qualitative resistance genes from wild tomato species have been deployed in tomato cultivars, but none confer resistance to all current lineages of the pathogen (Foolad *et al.* 2008). Quantitative resistance has been demonstrated in interspecific crosses between *S. lycopersicum* and several wild tomato species, including *S. pimpinellifolium*, *S. pennellii*, and *S. habrochaites* (Frary *et al.* 1998; Brouwer *et al.* 2004; Smart *et al.* 2007; AVRDC 2008). However, to our knowledge quantitative resistance to *P. infestans* has not been incorporated into commercial tomato cultivars.

Quantitative resistance to *P. infestans* has been found in the wild species *S. habrochaites* (Lobo and Navarro 1987; Black *et al.* 1996), and was mapped in interspecific cultivated × wild tomato populations derived from *S. habrochaites* accession LA2099 (Brouwer *et al.* 2004). QTL were detected by Brouwer *et al.* (2004) on all twelve chromosomes, with some QTL showing significance across multiple assay methods and environments. Three of the most consistently detected QTL, located on chromosomes 4, 5, and 11, were selected for further study, and near-isogenic lines (NILs) and sub-NILs were created for QTL validation and fine-mapping (Brouwer and St.Clair 2004). The QTL on chromosome 5 conferred foliar resistance, while the QTL on chromosomes 4 and 11 conferred both foliar and stem resistance. The introgressed regions containing the resistance QTL were also associated with some horticultural traits such as maturity, yield, fruit size, and canopy density, suggesting either pleiotropy or linkage between the *S. habrochaites* resistance alleles and the horticultural trait alleles (Brouwer and St.Clair 2004). Two of the resistance QTL from *S. habrochaites*, located on chromosomes 5 and 11 (designated *lb5b* and *lb11b*, respectively), were targeted for further QTL dissection and characterization.

The objectives of the current study were: (1) to perform higher-resolution mapping on two major *P. infestans* resistance QTL on chromosomes 5 and 11 [*lb5b* and *lb11b*, respectively, per Brouwer *et al.* (2004)] introgressed from *S. habrochaites* and to identify markers that are tightly linked to the QTL; (2) to determine whether each introgressed QTL region is composed of a single QTL or multiple resistance QTL; (3) to compare resistance QTL detected in replicated field and growth chamber experiments; and (4) to determine whether the QTL detected are isolate-specific or confer resistance to multiple isolates.

## Materials and Methods

### Plant material

Parental near-isogenic line (NIL) plant material was developed as described previously (Brouwer *et al.* 2004; Brouwer and St.Clair 2004). Briefly, an interspecific backcross population was developed from a cross between susceptible *S. lycopersicum* cv. NC84173 and resistant wild *S. habrochaites* accession LA2099. Parental genotypes, origins, characteristics, and seed sources are described by Brouwer *et al.* (2004). Resistance QTL were identified in the BC_1_ generation (Brouwer *et al.* 2004). Subsequently, additional backcrossing of selected BC_1_ progeny to susceptible *S. lycopersicum* cv. Hypeel45 was performed, with both foreground and background marker-assisted selection (MAS) employed each backcross generation, to produce advanced generation NILs that each contained a single target QTL region. Hypeel45, which is resistant to *Tobacco mosaic virus*, was used as the recurrent parent to facilitate propagation and maintenance of plant materials. After fine-mapping three of the resistance QTL (Brouwer and St.Clair 2004), two NILs, each containing the wild allele at either QTL *lb5b* or *lb11b* on chromosomes 5 and 11, respectively, were selected for further study. To create the materials used in the present study, *lb5b* and *lb11b* NILs were each individually backcrossed to susceptible *S. lycopersicum* cv. E6203, and the progeny was allowed to self-pollinate, generating two independent populations of BC_6_S_1_ sub-NILs. E6203 was used as the recurrent parent for the final backcross generation to incorporate genetic material that was widely used in California processing tomato germplasm. Heterozygous recombinant sub-NILs for the target QTL regions were marker-selected and allowed to self-pollinate, and recombinant homozygotes were marker-selected (BC_6_S_2_ generation). MAS was used throughout line development to maintain the desired alleles in the target QTL regions.

From the two BC_6_S_2_ sub-NIL populations, a total of 120 homozygous sub-NILs were selected and used as the principle material for our experiments: 58 for the *lb5b* introgression on chromosome 5 (introgression designated as chr5) and 62 for the *lb11b* introgression on chromosome 11 (introgression designated as chr11). Selection of the sub-NILs was based on genotypic class, as defined by the unique *S. habrochaites* introgression segments that they contained (see supporting information, Table S2 and Table S3 for marker genotypes). At least two independently generated individuals were selected to represent each genotypic class, unless only one was available. Seed sufficient for replicated field and growth chamber experiments was obtained for the 120 sub-NILs by allowing plants to self-pollinate in the greenhouse.

The 120 sub-NILs representing chr5 and chr11 were evaluated together for the growth chamber and 2009 field experiments. For the 2010 field experiments, to enable increased replication per line for enhanced mean estimation within our resource limitations, we selected a subset of 83 sub-NILs (41 for chr5 and 42 for chr11) from the original 120 lines that reduced both phenotypic and genotypic redundancy (see Table S2 and Table S3 for marker genotypes). To select lines for use in the 2010 field experiments, a preliminary analysis was performed on the 2009 field data. If a genotypic class showed significant phenotypic diversity for late blight resistance and/or horticultural traits in 2009 (data not shown), sub-NIL representatives of each phenotype within the genotypic class were maintained in the 2010 subset. The 83 sub-NILs from chr5 and chr11 were evaluated together in the 2010 field experiments.

### Genotyping

DNA was prepared from young leaves harvested from plants grown in a greenhouse at UC Davis using a modified CTAB mini-prep procedure (Fulton *et al.* 1995). Two generations of sub-NILs were genotyped: the BC_6_S_1_ generation was subject to MAS to generate a linkage map for each introgressed region (chr5 and chr11), and the BC_6_S_2_ generation was used to verify marker genotypes and select lines for phenotypic evaluation. Sequential genotyping was performed in the BC_6_S_1_ generation as described by Blair *et al.* (2003). PCR-based markers for chr5 and chr11, including SCARs, CAPs, and SSRs, were used to genotype each generation (Table S1). The PCR reaction conditions were 2 min initial denaturation at 94°, 35 cycles of amplification (45 sec denaturation at 94°, 1 min annealing, 1 min extension at 72°), and 5 min final extension at 72°. PCR products from the CAP and SSR markers were digested using restriction enzymes (Table S1), all PCR products were visualized using 1.5–3% agarose gels stained with ethidium bromide, and marker alleles were scored visually.

### *P. infestans* isolates and inoculum preparation

Three *P. infestans* isolates of the A1 mating type representing the isolate diversity found in California tomato-growing regions were obtained from a Phytophthora collection at University of California, Riverside: p9175 was isolated from Gonzales, Monterrey County in 1995; p7629 was isolated from San Luis Rey, San Diego County in 1981; and p10353 was isolated from Hollister, San Benito County in 2003.

A fourth isolate, Sal10, was collected from a natural *P. infestans* infestation that occurred in our Salinas field experiments in Monterrey County in 2010. Isolation was performed according to the CIP laboratory manual protocol (International Potato Center 2001). Plastic sample box chambers were lined with filter paper and autoclaved for 30 min. Immediately before use, they were surface sterilized with 70% ethanol, and 10 ml ddH_2_O was added to each chamber to maintain ∼100% relative humidity. The sample boxes were incubated at 16–18° with indirect light and a 14 hr daylight period, with incubation time varying for different steps in the procedure. After the isolation procedure had been completed, sporangia or mycelia were collected using either a platinum loop or sterile damp filter paper squares and transferred onto Rye B media (Caten and Jinks 1968; International Potato Center 2001) containing rifampicin, vancomycin, and nystatin antibiotics (RVN).

To maintain virulence, all isolates were passaged monthly on leaf tissue from susceptible tomato cultivar E6203 in sterile sample boxes using the incubation conditions detailed above. Tomato leaflets were surface sterilized in 10% bleach solution for 5 min, then rinsed twice in ddH_2_O for 5 min each. Leaflets were then placed in sample boxes that had been prepared as described above. Leaflets were inoculated using either 20 μl drops of inoculum at ∼1 × 10^4^ spores/ml or mycelia-covered agar plugs. After 7–10 days, sporangia or mycelia from the leaflets were harvested and transferred onto RVN media. After 14 days, agar plugs from the RVN plates were transferred to non-antibiotic plates for growth and maintenance. The isolates were grown on either Rye A, Rye B (Caten and Jinks 1968; International Potato Center 2001), or Rye/V8 media, depending on which media yielded the best mycelia growth and sporangia formation. Rye/V8 media was prepared in a similar manner to Rye B media. After the aqueous solution containing 50 g rye seeds was filtered and the seeds discarded, 50 ml V8 juice, 0.2 g CaCO_3_, and 17 g agar were added to the filtrate. The final volume was adjusted to 1 L, and the media was autoclaved for 30 min. All cultures on media plates were incubated under the same conditions as the sample boxes, as described above.

The culture plates were used for inoculum preparation when the mycelia reached the edge of the plate (7–14 days). Inoculum was obtained by adding 10 ml ddH_2_O to each plate and chilling for 2.5–3 hr at 4°. The plates were gently scraped with a glass rake prior to collecting the inoculum concentrate. The sporangia concentration was determined using a hemacytometer (Fisher Scientific) and adjusted to the desired concentration with ddH_2_O.

### Field experiments

In 2009 field experiments (denoted subsequently as 09FD_120), the 120 sub-NILs, two susceptible cultivar controls (Hypeel 45 and E6203), and two resistant controls (*lb5b* NIL and *lb11b* NIL) were grown in replicated experiments at two fields (hereafter referred to as Loc1 and Loc2) at the U.S. Department of Agriculture-Agricultural Research Service (USDA-ARS) Field Station in Salinas, CA. Loc1 had a loam soil, and Loc2 a sandy loam. Standard field practices for processing tomato were used at both locations, with sprinkler irrigation as needed. The field plots were arranged in a randomized complete block design (RCBD) with three blocks per location. Seedlings were grown for six weeks in a greenhouse at UC Davis, and then transplanted into the fields on June 18. The sub-NILs and controls were grown in five-plant plots with one plot per genotype per block. The experiments included multiple identical control plots per block for phenotypic trait comparison purposes during data collection (*i.e.* E6203-A and E6203-B) (see Table S2 and Table S3). The plants within a plot were spaced 0.30 m apart, and rows were spaced 1.02 m apart. On September 15 (89 days after transplanting), during the fruit set stage of plant development, the fields were inoculated with local isolate p9175 at a concentration of 1 × 10^3^ sporangia/ml. To apply inoculum, a spray wand of a backpack sprayer was inserted into the middle of the plant canopy at two points, at the second and fourth plants in each five-plant plot, and the spray trigger was depressed for approximately two seconds. Field observations suggest that there may have been a natural *P. infestans* infestation present in addition to the infection from our inoculum. Data collection began at the first observation of disease symptoms. Loc1 was scored for foliar and stem infection on three dates (9/23, 9/26, and 9/30), and Loc2 was scored on four dates (9/26, 9/30, 10/3, and 10/9).

In 2010 field experiments (denoted hereafter as 10FD_83), a subset of 83 sub-NILs (see *Plant Materials* and Table S2 and Table S3) and the same four controls were grown in replicated experiments at Loc1 and Loc2 in Salinas, CA, using the same field practices, plot size, and spacing as in 2009. The experiments included multiple identical control plots per block for phenotypic trait comparison purposes during data collection (see Table S2 and Table S3). The field plots were arranged in a RCBD with five blocks per location. Greenhouse-grown 6-week-old seedlings were transplanted into the fields on June 24. One block in Loc2 was lost shortly after transplanting due to bacterial speck disease and was omitted from the experiment. Natural *P. infestans* infestation occurred in the fields in early September (89 days after transplanting) during the tomato flowering stage, obviating the need for inoculation. Loc1 was scored for infection on six dates (9/21, 9/24, 10/1, 10/7, 10/25, and 10/28), and Loc2 was scored on seven dates (9/21, 9/24, 10/1, 10/7, 10/25, 10/28, and 11/1).

Each plot was evaluated for *P. infestans* symptom progression on the leaves and stems on multiple dates, as noted above. Foliar and stem disease scoring scales were slightly modified from those described by Brouwer and St.Clair (2004). The foliar symptom scale, based on the percentage of the foliage covered by lesions, was defined as: 1 = 0–5%, 2 = 6–25%, 3 = 26–50%, 4 = 51–80%, 5 = 81–95%, 6 = 96–99%, 7 = 100%. The stem symptom scale, based on the average size and appearance of the stem lesions, was defined as: 0 = no lesions, 1 = small spot or threadlike lesions, 2 = coalescing lesions ≤ 2 cm long, 3 = coalescing lesions > 2 cm long, 4 = lesions completely covering 3+ internodes, 5 = lesions with stem necrosis evident. From these symptom scores (File S1), area under the disease progress curve (AUDPC), as defined by Shaner and Finney (1977), was calculated for both foliar and stem disease symptom progression. The AUDPC formula is:

$$AUDPC=\;\overset{n}{\underset{i=1}{\sum }}[(Y_{i+n1}+Y_{i})/2][X_{i+1}-X_{i}]$$

where *Y_i_* is disease severity at the *i*^th^ observation, *X_i_* is the time at the *i*^th^ observation, and *n* is the total number of observations (Shaner and Finney 1977). To facilitate the comparison of disease score data across experiments with different intervals of time, all AUDPC calculations were standardized such that the time between the onset of disease symptoms (*i.e.* the first scoring date) and final scoring date was equivalent to 1. Each interval between scoring dates (*X_i+1_ – X_i_*) was divided by the total length of time between the first and last scores, thus becoming a proportion of 1. For example, for four scoring dates with intervals (*X_i+1_ – X_i_*) of 3, 4, and 5 days, the total timeframe of 12 days is set to a value of 1, and the intervals become 3/12 = 0.25, 4/12 = 0.33, and 5/12 = 0.42. Lower AUDPC values indicate less disease symptom progress, and are therefore indicative of increased disease resistance. The variables LEAF and STEM refer to the AUDPC values calculated for foliar and stem disease progression, respectively, and are subsequently employed throughout the text as higher-level group terms for trait-experiment combinations.

### Growth chamber (GC) experiments

The 120 sub-NILs and 4 controls that were used in the 09FD_120 experiments were also evaluated for foliar and stem symptoms in replicated growth chamber experiments (denoted subsequently as GC_120) inoculated with individual isolates of *P. infestans*. The experiments were performed over the course of 18 months. They employed a RCBD with temporal blocking. One randomized replication of all experimental lines fit within a growth chamber, and blocks were repeated over time in multiple chambers. There were six 73-cell flats per replication, which contained the randomized experimental lines in 3-plant plots. Three Conviron growth chambers (designated as GC-1, GC-2, and GC-3) at UC Davis were used for these experiments. They were set to identical conditions of 14 hr daylight period with 16° days / 14° nights and 95+% relative humidity. Wide-spectrum light was provided by a combination of metal halide and high-pressure sodium lights. Overhead mist systems in the chambers were set for 10 sec mist every 10 min for the first 24 hr post-inoculation, and then 5 sec mist every 3 hr for the remainder of the experiment. Plants were seeded out in flats and grown in a greenhouse until they reached the 4-true-leaf stage, and then the flats were transferred into the chambers and inoculated.

Flats in a chamber were inoculated with one of three isolates: p7629, p10353, or Sal10 (the isolate collected from the 2010 field experiments). These isolates represented the isolate diversity in California, and they were used to determine whether the detected QTL showed isolate-specificity. For p7629 and p10353, the inoculum concentration used was ∼2.5 × 10^3^ sporangia/ml. For Sal10, a concentration of ∼1 × 10^4^ was determined to provide even infection within the chambers. The final data set consisted of nine, eight, and six replications of p7629, p10353, and Sal10, respectively. Numbers of replications per isolate varied due to culturing and growth differences among isolates.

Plants were assessed individually for foliar and stem disease symptom progression starting 5–7 days post-inoculation at the onset of disease symptoms in the susceptible controls, and they were scored every two days for the 2–3 week duration of disease progression (File S2). Data collection for each replication was terminated when the majority of the susceptible controls in a chamber reached 100% coverage with foliar lesions. The disease symptom scoring scales for LEAF and STEM were the same as those used in the field experiment (described previously), and the standardized AUDPC was calculated from the mean score for each three-plant plot as described above.

### Statistical analyses

All data were subjected to normality tests, homogeneity of variances (HOV) tests, and analysis of variance (ANOVA), as described below. The chr5 and chr11 populations were analyzed separately for each experiment because the two populations were independently generated, as described previously. As mentioned previously, LEAF and STEM refer to the AUDPC values calculated for foliar and stem disease symptom progression in all experiments, and chr5 and chr11 refer to the chromosome 5 and 11 introgression regions, respectively.

LEAF and STEM data were subjected to the Shapiro-Wilk test for normality and the Levene’s test for HOV, which were performed using SAS v9.1 software (SAS Institute, Cary, NC). The assumption of normality was met when Shapiro-Wilk W > 0.95. A Levene’s test for HOV was considered significant when *P* ≤ 0.05 for a given factor in the linear additive model, indicating heterogeneity of variances (heteroscedasticity), in which case data were weighted by the inverse of the variance for that factor. When the Levene’s test showed an interaction to be significant but neither of the main factors was significant, the analysis was weighted by both of the main factors. Genotype was not used as a weighting factor because the high degrees of freedom and low F value for this factor would make the weights negligible. ANOVAs were performed for all LEAF and STEM data, and an effect of *P* ≤ 0.05 was considered significant. For the GC analyses with all isolates, the number of sample points exceeded the maximum allowed in SAS for the Shapiro-Wilk test. To calculate a W value equivalent to that generated by the Shapiro-Wilk test, ranks were calculated from the residual errors, and the ranks and residual errors were correlated. The assumption of normality was met if the correlation coefficient (equivalent to W) was greater than 0.95. When a significant factor*Genotype interaction (*e.g.* Loc*Genotype) was detected in ANOVA, each factor (*e.g.* Loc1 and Loc2) was re-analyzed separately for accurate estimation of genotypic means for all sub-NILs and controls for use in means separation and QTL mapping (see below).

#### Field ANOVAs:

The field data were analyzed for chr5 and chr11 populations with the general linear model procedure (Proc GLM) in SAS using the linear additive model (LAM):

$$\text{LEAF or STEM}=\text{Loc}+\text{ Block(Loc)}+\text{Genotype}+{\text{ Loc}}^{*}\text{Genotype}$$

where Loc was the location, Genotype was the individual control or sub-NIL within chr5 or chr11, a * indicated an interaction, and parentheses indicated a nested variable. Block(Loc) was considered a random variable.

In 09FD_120, chr5 LEAF had a significant (*P* ≤ 0.05) Loc*Genotype interaction; therefore, each location was analyzed separately. In 10FD_83, data collection occurred during two distinct time periods due to an intervening period of unusually high daytime temperatures that temporarily interrupted *P. infestans* disease progression. There was re-growth of leaf tissue in many plots, which caused a decrease in the foliar disease rating. Therefore, disease symptom scores from the two time periods were analyzed separately: “early” includes the symptom scores from the first four data collection dates, and “late” includes the scores from the final two to three dates. Several 10FD-early_83 and 10FD-late_83 analyses demonstrated a significant Loc*Genotype interaction; therefore, the two locations were analyzed separately. These analyses include 10FD-early_83 chr5 and chr11 STEM, 10FD-late_83 chr11 LEAF, and 10FD-late_83 chr5 and chr11 STEM. All other ANOVAs for 09FD_120 and 10FD_83 evaluated both locations concurrently.

#### Growth chamber ANOVAs:

GC_120 experiment data were analyzed using a mixed model procedure (Proc MIXED) in SAS due to an unbalanced design resulting from the fact that there were six plots of each of the controls per replication to monitor disease progression within a chamber, but only one plot of each sub-NIL. During the experiments, spatial heterogeneity of symptom development was observed within the chambers and was related to whether a plot was located on the edge or inside of a flat and the position of a flat within the chamber. To account for this variation, the variables Edge and FlatPos were included in the model. The LAM for the analysis of GC data is:

$$\begin{array}{c} \text{LEAF or STEM}=\text{Isolate}+\text{Genotype}+\text{Chamber}\\ +{\text{ Block(Isolate}}^{*}\text{Chamber)}+\text{ Edge}\\ +\text{ FlatPos}+{\text{ Isolate}}^{*}\text{Genotype}+{\text{ Isolate}}^{*}\text{Chamber}+{\text{ Isolate}}^{*}\text{Edge}\\ +{\text{ Isolate}}^{*}\text{FlatPos}+{\text{ Chamber}}^{*}\text{Genotype}\\ +{\text{ Chamber}}^{*}\text{Edge +}{\text{ Chamber}}^{*}\text{FlatPos}\\ +{\text{ Edge}}^{*}\text{FlatPos} \end{array}$$

Block(Isolate*Chamber) was considered a random variable. For isolate p7629, there was one of the three chambers in which only one replication (instead of two) was completed. Consequently, SAS was unable to calculate the means using the above LAM because the effects of the Chamber interaction terms could not be estimated, so these terms were removed from the p7629 model. The p7629 LAM is:

$$\begin{array}{l} \text{LEAF or STEM}=\text{Genotype}+\text{Chamber}+\text{Block(Chamber)}\\ +\text{Edge}+\text{ FlatPos}+{\text{ Edge}}^{*}\text{FlatPos} \end{array}$$

In GC_120 LEAF and STEM, for both chr5 and chr11, there were significant (*P* ≤ 0.05) Isolate*Genotype interactions, so for each trait the isolates were analyzed separately. In the chr11 Sal10 STEM analysis, there was a significant (*P* ≤ 0.05) Chamber*Genotype interaction; thus, analysis was performed by Chamber.

#### Means separation:

Means separation in SAS was performed on the trait means in all experiments. In the field data analyses, means separation was performed using Proc MEANS with Tukey’s Honestly Significant Difference (HSD) test. In the GC analyses, Tukey’s HSD test was performed using least squares means via a macro in SAS (Saxton 1998).

#### Correlations:

For 09FD_120, GC_120, and 10FD_83, Pearson correlation coefficients (*r*) between LEAF and STEM genotypic means within each experiment were obtained using Proc CORR in SAS. Correlations of genotypic means comparing LEAF or STEM traits across locations and isolates were also performed between GC_120 and 09FD_120 traits.

To compare across experiments that differed in the number of sub-NILs (09FD_120 and GC_120 with 10FD_83), the 120-line datasets were reduced to the same 83 sub-NILs that were evaluated in the 10FD_83 (referred to as 09FD_83 and GC_83, respectively). Subsequently, 09FD_83 and GC_83 were analyzed using the methods described previously to obtain correlations across experiments.

### Linkage and QTL mapping

Linkage maps for the chr5 and chr11 *S. habrochaites* introgressed regions were constructed via JoinMap 3.0 (van Ooijen and Voorrips 2001) using 652 BC_6_S_1_ sub-NIL individuals for chr5 and 852 BC_6_S_1_ sub-NIL individuals for chr11. The Kosambi function with a 3-LOD significance threshold was used to construct the maps.

Composite interval mapping (CIM) was performed for each trait using sub-NIL means obtained from ANOVA (see above) and WinQTLCartographer2.5 (Wang *et al.* 2011). For the purposes of analysis and discussion, a trait was defined as the combination of disease scoring method (LEAF or STEM) and the experiment and location or isolate in which it was evaluated (*e.g.* 09FD_120 Loc1 LEAF). Significance thresholds for each trait were calculated at *P* = 0.05 significance using 2000 permutations, and a QTL was considered significant at *P* ≤ 0.05 if the peak LOD value exceeded this permuted threshold. QTL mapping was performed using CIM Model 6 (Standard Model) and the forward and backward regression method with a walkspeed of 1 cM and a window size of 2 cM. Multiple QTL were declared for a single trait when the LOD values between significant (*P* ≤ 0.05) peaks on the same linkage group decreased below the significance threshold for at least two contiguous markers.

Each QTL mapped in this study was named using the following nomenclature: experiment (09FD, 10FD, or GC), number of sub-NILs included in the data set (120 or 83), field location or isolate (L1, L2; p76, p10, or sal), disease trait (Lf or St), and chromosome introgression (5 or 11). In 10FD, early and late datasets were identified with an ‘e’ or ‘l’ after 10FD, respectively. In GC, if only one of the chambers was used in the analysis, the number of that chamber is placed after GC (*e.g.* GC-1). For location, L1 and L2 refer to Loc1 and Loc2, and L12 indicates that both locations were analyzed together. For isolate, p76, p10, and sal refer to p7629, p10353, and Sal10, respectively. For introgression, 5 and 11 refer to chr5 and chr11, respectively. Sequential numbers (*e.g.* -1, -2) at the end of a QTL name differentiate between multiple QTL detected in a single analysis. For example, 10FDe_83_L12_Lf5-1 is the name of the first QTL detected in the 10FD-early, 83-lines, Loc1 and Loc2, LEAF chr5 analysis.

**Figure 1:**
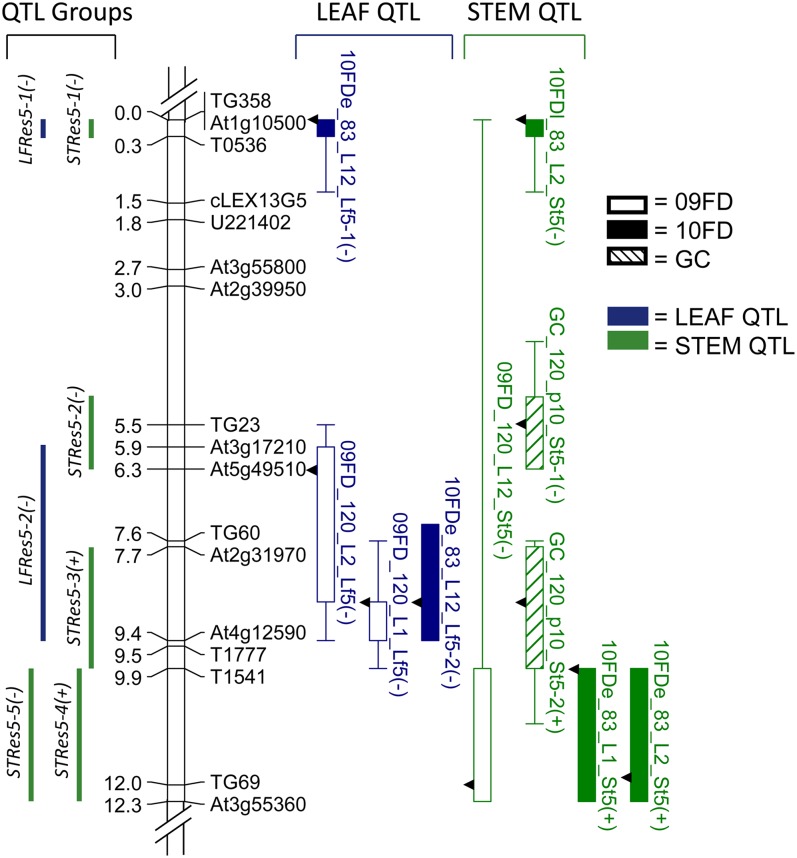
Linkage map of chromosome 5 introgressed region from *S. habrochaites* and QTL for LEAF and STEM traits. Left of linkage map are QTL group locations and distances in cM; right of linkage map are QTL detected for LEAF and STEM traits. Boxes and whiskers show 1-LOD and 2-LOD intervals, respectively. Arrows on QTL bars indicate LOD peak locations. QTL names are given by dataset, location or isolate, and trait evaluated (see *Materials and Methods*). The effect of the *S. habrochaites* allele at a QTL is indicated after the QTL name: a minus sign (−) indicates a decrease in AUDPC and thus an increase in resistance.

**Figure 2:**
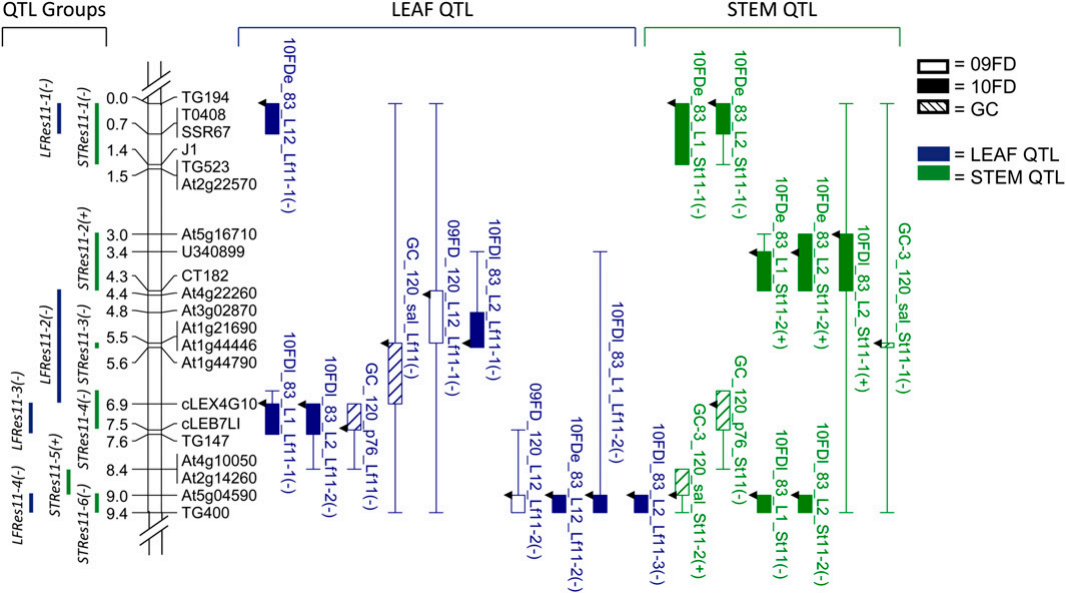
Linkage map of chromosome 11 introgressed region from *S. habrochaites* and QTL for LEAF and STEM traits. Left of linkage map are QTL group locations and distances in cM; right of linkage map are QTL detected for LEAF and STEM traits. Boxes and whiskers show 1-LOD and 2-LOD intervals, respectively. Arrows on QTL bars indicate LOD peak locations. QTL names are given by dataset, location or isolate, and trait evaluated (see *Materials and Methods*). The effect of the *S. habrochaites* allele at a QTL is indicated after the QTL name: a minus sign (−) indicates a decrease in AUDPC and thus an increase in resistance.

For each introgressed region (spanning portions of chromosomes 5 and 11), a linkage map showing QTL locations for each trait was constructed using MapChart2.1 (Voorrips 2002). QTL locations are indicated as 1-LOD bars and 2-LOD whiskers on the linkage maps (Figures 1 and 2). For ease of comparison across experiments, QTL were placed into groups based on STEM or LEAF, coincidence of the 1-LOD intervals, and directionality of the wild allele phenotypic effect.

## Results

### Statistical analyses

#### ANOVAs:

The set of 120 sub-NILs and 4 controls was analyzed for late blight disease symptoms in 2009 field and growth chamber experiments (09FD_120 and GC_120, respectively). A subset of 83 sub-NILs and controls was also evaluated in the field during 2010 for disease resistance (10FD_83). LEAF and STEM refer to the AUDPC values calculated for foliar and stem disease symptom progression in all experiments, and chr5 and chr11 refer to the chromosome 5 and 11 introgression regions, respectively. R^2^ values were generally higher for the GC_120 analyses than for the 09FD_120 and 10FD_83 analyses (Table 1). For chr5, genotype had a significant effect (*P* ≤ 0.05) in all 09FD_120 and 10FD_83 analyses, and in most GC_120 analyses. For chr11, there was a significant genotypic effect in all 09FD_120 and 10FD_83 analyses, and in most GC_120 analyses (Table 1).

**Table 1:** Summary of ANOVAs from all analyses

			F value		
Chr	Dataset	Trait	Genotype	Loc or Chamber*^a^*	R^2^
Chr5	09FD_120	09FD_120 Loc1 LEAF	1.69 **	–	0.47
		09FD_120 Loc2 LEAF	2.77 ***	–	0.77
		09FD_120 Loc1&2 STEM	5.03 ***	0.01 ^ns^	0.64
	10FD_83	10FD-early_83 Loc1&2 LEAF	7.02 ***	68.70 ***	0.77
		10FD-early_83 Loc1 STEM	5.63 ***	–	0.59
		10FD-early_83 Loc2 STEM	5.78 ***	–	0.66
		10FD-late_83 Loc1&2 LEAF	4.49 ***	0.02 ^ns^	0.50
		10FD-late_83 Loc1 STEM	1.72 **	–	0.43
		10FD-late_83 Loc2 STEM	4.40 ***	–	0.61
	GC_120	GC_120 p7629 LEAF	3.67 ***	0.82 ^ns^	0.60
		GC_120 p10353 LEAF	2.58 ***	0.02 ^ns^	0.78
		GC_120 Sal10 LEAF	1.88 ***	0.18 ^ns^	0.81
		GC_120 p7629 STEM	2.36 ***	1.25 ^ns^	0.55
		GC_120 p10353 STEM	1.75 ***	0.03 ^ns^	0.67
		GC_120 Sal10 STEM	1.36 ^ns^	0.22 ^ns^	0.74
Chr11	09FD_120	09FD_120 Loc1&2 LEAF	2.22 ***	19.00 *	0.74
		09FD_120 Loc1&2 STEM	2.77 ***	1.30 ^ns^	0.55
	10FD_83	10FD-early_83 Loc1&2 LEAF	6.42 ***	60.36 ***	0.74
		10FD-early_83 Loc1 STEM	4.12 ***	–	0.52
		10FD-early_83 Loc2 STEM	4.93 ***	–	0.62
		10FD-late_83 Loc1 LEAF	5.10 ***	–	0.62
		10FD-late_83 Loc2 LEAF	3.01 ***	–	0.52
		10FD-late_83 Loc1 STEM	2.31 ***	–	0.45
		10FD-late_83 Loc2 STEM	3.98 ***	–	0.60
	GC_120	GC_120 p7629 LEAF	1.90 ***	0.49 ^ns^	0.53
		GC_120 p10353 LEAF	1.95 ***	0.01 ^ns^	0.63
		GC_120 Sal10 LEAF	2.05 ***	0.29 ^ns^	0.78
		GC_120 p7629 STEM	2.59 ***	0.44 ^ns^	0.54
		GC_120 p10353 STEM	1.64 **	0.04 ^ns^	0.61
		GC-1_120 Sal10 STEM	1.40 ^ns^	–	0.84
		GC-2_120 Sal10 STEM	1.21 ^ns^	–	0.78
		GC-3_120 Sal10 STEM	2.42 ***	–	0.66

Within each chromosome, trait names are given according to the dataset, the location or isolate, and the trait evaluated (see *Materials and Methods* for details). F values are presented for all relevant main effects included in the model. R^2^ indicates the fit of the data to the linear additive model for each analysis. ns = not significant. ^*^P ≤ 0.05; ^**^P ≤ 0.01; ^***^P ≤ 0.001.

a Location (Loc) was tested in the Field experiments; Chamber was tested in the GC experiments. A dash (‑) means not included in model.

#### Means separation:

There were significant (*P* ≤ 0.05) differences among genotype means for most traits analyzed (Table S2 and Table S3). There were several lines within each population that had the lowest mean AUDPC for multiple traits. For chr5, these lines included 08GH6947 and LB5-NIL-A. For chr11, these lines included 08GH4659 and LB11-NIL-B. The experiments included multiple identical control plots per block for disease phenotypic trait comparison purposes during data collection (Table S2 and Table S3). In general, identical controls plots in a given experiment had means that were tightly grouped. However, control cultivar E6203 sometimes exhibited a range of AUDPC values in the means separation, although the differences were not significant.

#### LEAF-STEM correlations:

Pearson correlation coefficients (*r*) were obtained for LEAF and STEM genotypic means within each experiment (Table 2). All significant (*P* ≤ 0.05) LEAF-STEM correlations were positive. With the exception of chr11 10FD-late_83 (*r* = 0.39–0.57), the LEAF-STEM correlations in the field experiments were either significant but weak (*r* = 0.16–0.41) or not significant (*P* > 0.05). The GC LEAF-STEM correlations were moderate to high (*r* = 0.46–0.86) and highly significant (*P* ≤ 0.001).

**Table 2:** Pearson correlations between leaf and stem within each experiment

Chr	Experiment	*r*
Chr 5	09FD_120 Loc1	ns
	09FD_120 Loc2	ns
	10FD-early_83 Loc1	ns
	10FD-early_83 Loc2	ns
	10FD-late_83 Loc1	0.40 **
	10FD-late_83 Loc2	ns
	GC_120 p7629	0.62 ***
	GC_120 p10353	0.76 ***
	GC_120 Sal10	0.75 ***
Chr 11	09FD_120 Loc1&2	ns
	10FD-early_83 Loc1	ns
	10FD-early_83 Loc2	ns
	10FD-late_83 Loc1	0.57 ***
	10FD-late_83 Loc2	0.49 ***
	GC_120 p7629	0.63 ***
	GC_120 p10353	0.73 ***
	GC-1_120 Sal10	0.76 ***
	GC-2_120 Sal10	0.69 ***
	GC-3_120 Sal10	0.67 ***

Correlations (*r*) were performed by genotype means. Experiment names are given according to the dataset and the Location or Isolate (see *Materials and Methods* for details). ns = not significant. ^*^P ≤ 0.05; ^**^P ≤ 0.01; ^***^P ≤ 0.001.

#### LEAF-LEAF and STEM-STEM correlations:

Pearson correlations were obtained between both LEAF and STEM genotypic means across different experiments for the chr5 and chr11 populations (Table 3). Correlations coefficients (*r* ≥ 0.40) are summarized in Table 3, all of which were significant at *P* ≤ 0.01. All chr5 and chr11 trait correlations were positive. For chr5 LEAF traits, the only strong correlation was between 10FD-early_83 Loc1&2 and 10FD-late_83 Loc1&2 (*r* = 0.58) (Table 3). For chr5 STEM traits, the most consistently significant and moderate to strong correlations were between 09FD_83, 10FD-early_83, and 10FD-late_83 (*r* = 0.30–0.78) (Table 3).

**Table 3:** Pearson correlations between leaf or stem traits in Chr5 and Chr11 across experiments

Chr	LEAF / STEM	Trait 1	Trait 2	*r*
Chr 5	LEAF-LEAF	10FD-early_83 Loc1&2	10FD-late_83 Loc1&2	0.58 ***
	STEM-STEM	09FD_83 Loc1&2	10FD-early_83 Loc1	0.66 ***
		09FD_83 Loc1&2	10FD-early_83 Loc2	0.67 ***
		09FD_83 Loc1&2	10FD-late_83 Loc1	0.41 **
		09FD_83 Loc1&2	10FD-late_83 Loc2	0.54 ***
		10FD-early_83 Loc1	10FD-early_83 Loc2	0.78 ***
		10FD-early_83 Loc1	10FD-late_83 Loc2	0.73 ***
		10FD-early_83 Loc2	10FD-late_83 Loc2	0.72 ***
		10FD-late_83 Loc1	10FD-late_83 Loc2	0.45 **
Chr 11	LEAF-LEAF	09FD_83 Loc1&2	10FD-early_83 Loc1&2	0.57 ***
		09FD_83 Loc1&2	10FD-late_83 Loc1	0.42 **
		10FD-early_83 Loc1&2	10FD-late_83 Loc1	0.68 ***
		10FD-early_83 Loc1&2	10FD-late_83 Loc2	0.49 ***
		10FD-late_83 Loc1	10FD-late_83 Loc2	0.51 ***
		GC_120 p7629	GC_120 p10353	0.50 ***
Chr 11	STEM-STEM	09FD_83 Loc1&2	10FD-early_83 Loc1	0.54 ***
		09FD_83 Loc1&2	10FD-early_83 Loc2	0.52 ***
		09FD_83 Loc1&2	10FD-late_83 Loc1	0.45 **
		09FD_83 Loc1&2	10FD-late_83 Loc2	0.44 **
		10FD-early_83 Loc1	10FD-early_83 Loc2	0.68 ***
		10FD-early_83 Loc1	10FD-late_83 Loc1	0.50 ***
		10FD-early_83 Loc1	10FD-late_83 Loc2	0.43 **
		10FD-early_83 Loc2	10FD-late_83 Loc1	0.47 **
		10FD-early_83 Loc2	10FD-late_83 Loc2	0.52 ***
		10FD-late_83 Loc1	10FD-late_83 Loc2	0.91 ***

Trait correlations (*r*) were performed between either LEAF or STEM genotypic means across experiments. Only significant correlations of *r* ≥ 0.40 are reported. Trait names are given according to the dataset and the Location or Isolate. ^*^P ≤ 0.05; ^**^P ≤ 0.01; ^***^P ≤ 0.001.

For chr11 LEAF traits, all of the correlations between 10FD-early_83, 10FD-late_83, and 09FD_83 were significant and moderate to strong (*r* = 0.39–0.68). There was also a moderately strong correlation between the different isolates of GC_120 (*r* = 0.50) (Table 3). For chr11 STEM traits, there were significant and moderate to strong correlations between the 09FD_83, 10FD-early_83, and 10FD-late_83 traits (*r* = 0.43–0.91) (Table 3).

### Linkage and QTL mapping

#### Chr5:

The linkage map of the chromosome 5 introgressed region (chr5) included 17 polymorphic markers that spanned 12.3 cM (Figure 1). The average intermarker distance was 0.7 cM and the largest gap was 2.5 cM, and all markers were linked at 6-LOD, above the 3-LOD significance threshold. Graphical genotypes of the chr5 BC_6_S_2_ sub-NILs that were selected for phenotypic evaluation are presented in Table S2. There were 10 QTL detected for trait means, which were placed into two LEAF and five STEM QTL groups (Table 4 and Figure 1). R^2^ values in this section are the percentage of phenotypic variation explained by the marker-trait association at the LOD peak of each QTL.

**Table 4:** Summary of QTL detected from all analyses

Chr	Trait	QTL	Group*^a^*	Peak Marker or Interval	LOD Peak / Threshold	R^2^
Chr5	09FD_120 Loc1 LEAF	09FD_120_L1_Lf5	*LFRes5-2*	At2g31970-At4g12590	4.27/1.75	0.29
	09FD_120 Loc2 LEAF	09FD_120_L2_Lf5	*LFRes5-2*	At5g49510	2.93/1.71	0.18
	09FD_120 Loc1&2 STEM	09FD_120_L12_St5	*STRes5-5*	T1541-TG69	1.89/1.72	0.18
	10FD-early_83 Loc1&2 LEAF	10FDe_83_L12_Lf5-1	*LFRes5-1*	TG358	4.76/1.72	0.21
		10FDe_83_L12_Lf5-2	*LFRes5-2*	At2g31970-At4g12590	7.77/1.72	0.47
	10FD-early_83 Loc1 STEM	10FDe_83_L1_St5	*STRes5-4*	T1541	2.74/1.70	0.21
	10FD-early_83 Loc2 STEM	10FDe_83_L2_St5	*STRes5-4*	T1541-TG69	3.63/1.72	0.21
	10FD-late_83 Loc2 STEM	10FDl_83_L2_St5	*STRes5-1*	TG358	5.43/1.77	0.41
	GC_120 p10353 STEM	GC_120_p10_St5-1	*STRes5-2*	TG23	3.01/1.71	0.19
		GC_120_p10_St5-2	*STRes5-3*	At2g31970-At4g12590	2.34/1.71	0.18
Chr11	09FD_120 Loc1&2 LEAF	09FD_120_L12_Lf11-1	*LFRes11-3*	At4g22260	1.89/1.63	0.07
		09FD_120_L12_Lf11-2	*LFRes11-5*	At5g04590	3.70/1.63	0.17
	10FD-early_83 Loc1&2 LEAF	10FDe_83_L12_Lf11-1	*LFRes11-1*	TG194	3.28/1.68	0.21
		10FDe_83_L12_Lf11-2	*LFRes11-5*	At5g04590	5.82/1.68	0.38
	10FD-early_83 Loc1 STEM	10FDe_83_L1_St11-1	*STRes11-1*	TG194	3.39/1.59	0.23
		10FDe_83_L1_St11-2	*STRes11-2*	U340899	4.27/1.59	0.31
	10FD-early_83 Loc2 STEM	10FDe_83_L2_St11-1	*STRes11-1*	TG194	2.44/1.64	0.18
		10FDe_83_L2_St11-2	*STRes11-2*	U340899	4.47/1.64	0.33
	10FD-late_83 Loc1 LEAF	10FDl_83_L1_Lf11-1	*LFRes11-4*	cLEX4G10	3.03/1.76	0.24
		10FDl_83_L1_Lf11-2	*LFRes11-5*	At5g04590	2.30/1.76	0.15
	10FD-late_83 Loc2 LEAF	10FDl_83_L2_Lf11-1	*LFRes11-3*	At1g21690	3.50/1.74	0.22
		10FDl_83_L2_Lf11-2	*LFRes11-4*	cLEB7L1	2.97/1.74	0.24
		10FDl_83_L2_Lf11-3	*LFRes11-5*	At5g04590	3.16/1.74	0.19
	10FD-late_83 Loc1 STEM	10FDl_83_L1_St11	*STRes11-6*	At5g04590	4.56/1.78	0.28
	10FD-late_83 Loc2 STEM	10FDl_83_L2_St11-1	*STRes11-2*	At5g16710	1.88/1.74	0.13
	10FD-late_83 Loc2 STEM	10FDl_83_L2_St11-2	*STRes11-6*	At5g04590	3.23/1.74	0.23
	GC_120 p7629 LEAF	GC_120_p76_Lf11	*LFRes11-4*	cLEX4G10	3.80/1.69	0.22
	GC_120 Sal10 LEAF	GC_120_sal_Lf11	*LFRes11-3*	At1g44446	1.87/1.61	0.12
	GC_120 p7629 STEM	GC_120_p76_St11	*STRes11-4*	cLEX4G10	3.06/1.68	0.18
	GC-3_120 Sal10 STEM	GC-3_120_sal_St11-1	*STRes11-3*	At1g21690	1.87/1.61	0.10
		GC-3_120_sal_St11-2	*STRes11-5*	At4g10050	3.35/1.61	0.21

Chr	Trait	QTL	Allele Dir*^b^*	1-LOD Interval	Flanking Markers
Chr5	09FD_120 Loc1 LEAF	09FD_120_L1_Lf5	(−)	8.7-9.4	At2g31970-At4g12590
	09FD_120 Loc2 LEAF	09FD_120_L2_Lf5	(−)	5.9-8.7	At3g17210-At4g12590
	09FD_120 Loc1&2 STEM	09FD_120_L12_St5	(−)	9.9-12.3	T1541-At3g55360
	10FD-early_83 Loc1&2 LEAF	10FDe_83_L12_Lf5-1	(−)	0.0-0.3	TG358-T0536
		10FDe_83_L12_Lf5-2	(−)	7.3-9.4	At5g49510-At4g12590
	10FD-early_83 Loc1 STEM	10FDe_83_L1_St5	(+)	9.9-12.3	T1541-At3g55360
	10FD-early_83 Loc2 STEM	10FDe_83_L2_St5	(+)	9.9-12.3	T1541-At3g55360
	10FD-late_83 Loc2 STEM	10FDl_83_L2_St5	(−)	0.0-0.3	TG358-T0536
	GC_120 p10353 STEM	GC_120_p10_St5-1	(−)	5.0-6.3	At2g33950-At5g49510
		GC_120_p10_St5-2	(+)	7.7-9.9	At2g31970-T1541
Chr11	09FD_120 Loc1&2 LEAF	09FD_120_L12_Lf11-1	(−)	4.3-5.5	CT182-At1g21690
		09FD_120_L12_Lf11-2	(−)	9.0-9.4	At4g10050-TG400
	10FD-early_83 Loc1&2 LEAF	10FDe_83_L12_Lf11-1	(−)	0.0-0.7	TG194-T0408
		10FDe_83_L12_Lf11-2	(−)	9.0-9.4	At5g04590-TG400
	10FD-early_83 Loc1 STEM	10FDe_83_L1_St11-1	(−)	0.0-1.4	TG194-J1
		10FDe_83_L1_St11-2	(+)	3.4-4.3	At5g16710-CT182
	10FD-early_83 Loc2 STEM	10FDe_83_L2_St11-1	(−)	0.0-0.7	TG194-T0408
		10FDe_83_L2_St11-2	(+)	3.0-4.3	At5g16710-CT182
	10FD-late_83 Loc1 LEAF	10FDl_83_L1_Lf11-1	(−)	6.9-7.6	cLEX4G10-TG147
		10FDl_83_L1_Lf11-2	(−)	9.0-9.4	At5g04590-TG400
	10FD-late_83 Loc2 LEAF	10FDl_83_L2_Lf11-1	(−)	4.8-5.6	At4g22260-At1g44790
		10FDl_83_L2_Lf11-2	(−)	6.9-7.6	cLEX4G10-TG147
		10FDl_83_L2_Lf11-3	(−)	9.0-9.4	At5g04590-TG400
	10FD-late_83 Loc1 STEM	10FDl_83_L1_St11	(−)	9.0-9.4	At5g04590-TG400
	10FD-late_83 Loc2 STEM	10FDl_83_L2_St11-1	(+)	3.0-4.3	AAt5g04590-TG400
	10FD-late_83 Loc2 STEM	10FDl_83_L2_St11-2	(−)	9.0-9.4	At5g04590-TG400
	GC_120 p7629 LEAF	GC_120_p76_Lf11	(−)	6.9-7.5	cLEX4G10-cLEB7L1
	GC_120 Sal10 LEAF	GC_120_sal_Lf11	(−)	5.5-6.9	At1g21690-cLEX4G10
	GC_120 p7629 STEM	GC_120_p76_St11	(−)	6.6-7.5	At1g44790-cLEB7L1
	GC-3_120 Sal10 STEM	GC-3_120_sal_St11-1	(−)	5.5-5.6	At1g21690-At1g44790
		GC-3_120_sal_St11-2	(+)	8.4-9.0	At4g10050-At5g04590

Trait and QTL names are given according to the dataset, location or isolate, and trait evaluated. QTL names also include the chromosome, and if multiple QTL were detected for a single trait, a dash and number was included to differentiate among QTL (see *Materials and Methods*). 1-LOD interval positions refer to the cM distances on the linkage map for each introgressed region from *S. habrochaites* on chromosome 5 or 11. The R^2^ values are the proportion of phenotypic variation explained by the marker-trait association.

a Group indicates coincident QTL, as defined by trait measured, directionality of the *S. habrochaites* allele effect, and coincidence of the 1-LOD intervals.

b Allele directionality is the direction of the effect of the *S. habrochaites* allele at that QTL: a minus sign (−) indicates a decrease in AUDPC and thus an increase in resistance.

For chr5 LEAF traits, there were four QTL detected in 09FD_120 and 10FD_83, and none detected in GC_120 (Table 4 and Figure 1). One group of coincident LEAF QTL was detected across experiments. This group, designated *LFRes5-2*, explained 18–47% of the phenotypic variation (%PV) and included three QTL: 09FD_120_L1_Lf5, 09FD_120_L2_Lf5, and 10FDe_83_L12_Lf5-2. One other LEAF QTL was not coincident with any other QTL, 10FDe_83_L12_Lf5-1 (group *LFRes5-1*), which explained 21%PV. The alleles associated with increased disease resistance for both *LFRes5-1* and *LFRes5-2* were from *S. habrochaites*.

For chr5 STEM traits, there were six QTL detected (Table 4 and Figure 1). There was one group of coincident QTL mapped across experiments. This group, *STRes5-4*, explained 21%PV and contained QTL 10FDe_83_L1_St5 and 10FDe_83_L2_St5. Four additional non-coincident QTL were detected: 10FDl_83_L2_St5 (group *STRes5-1*), GC_120_p10_St5-1 (*STRes5-2*), GC_120_p10_St5-2 (*STRes5-3*), and 09FD_120_L12_St5 (*STRes5-5*) explained 41, 19, 18, and 18%PV, respectively. The alleles associated with increased disease resistance in *STRes5-1*, *STRes5-2*, and *STRes5-5* were from *S. habrochaites*, and those in *STRes5-3* and *STRes5-4* were from *S. lycopersicum*.

#### Chr11:

The linkage map of the chromosome 11 introgressed region (chr11) included 21 polymorphic markers that spanned 9.4 cM (Figure 2). The average intermarker distance was 0.5 cM and the largest gap was 1.5 cM, and all markers were linked at 6-LOD, above the 3-LOD significance threshold. Graphical genotypes of the chr11 BC_6_S_2_ sub-NILs that were selected for phenotypic evaluation are presented in Table S3. There were 21 QTL detected for trait means, which were placed into four LEAF and six STEM QTL groups.

For chr11 LEAF traits, there were 11 QTL (Table 4 and Figure 2). There were three groups of coincident QTL detected across experiments. The first group, *LFRes11-2*, explained 7–22%PV and included three QTL: 09FD_120_L12_Lf11-1, 10FDl_83_L2_Lf11-1, and GC_120_sal_Lf11. The second group, *LFRes11-3*, explained 22–24%PV and contained three QTL: 10FDl_83_L1_Lf11-1, 10FDl_83_L2_Lf11-2, and GC_120_p76_Lf11. The third group, *LFRes11-4*, explained 15–38%PV and included four QTL: 09FD_120_L12_Lf11-2, 10FDe_83_L12_Lf11-2, 10FDl_83_L1_Lf11-2, and 10FDl_83_L2_Lf11-3. One QTL, 10FDe_83_L12_Lf11-1 (group *LFRes11-1*), was not coincident with any other QTL and explained 21%PV. The alleles associated with disease resistance in all QTL groups were from *S. habrochaites*.

For chr11 STEM traits, there were 10 QTL in total detected in 10FD_83 and GC_120, and none in 09FD_120 (Table 4 and Figure 2). Three groups of coincident QTL were detected across experiments. The first group, designated as *STRes11-1*, explained 18–23%PV and was comprised of two QTL: 10FDe_83_L1_St11-1 and 10FDe_83_L2_St11-1. The second QTL group, *STRes11-2*, explained 13–33%PV and consisted of three QTL: 10FDe_83_L1_St11-2, 10FDe_83_L2_St11-2, and 10FDl_83_L2_St11-1. The third group, *STRes11-6*, explained 23–28%PV and included QTL 10FDl_83_L1_St11 and 10FDl_83_L2_St11-2. There were three non-coincident QTL detected in the GC experiments: GC-3_120_sal_St11-1 (group *STRes11-3*), GC_120_p76_St11 (*STRes11-4*), and GC-3_120_sal_St11-2 (*STRes11-5*), which explained 10, 18, and 21%PV, respectively. The alleles associated with disease resistance in *STRes11-1*, *-3*, *-4*, and *-6* were from *S. habrochaites*, and those in *STRes11-2* and *-5* are from *S. lycopersicum*.

## Discussion

### QTL fractionation

With higher resolution mapping, two late blight resistance QTL regions (*lb5b* and *lb11b*) introgressed from *S. habrochaites* and fine-mapped in previous studies (Brouwer and St.Clair 2004) fractionated into multiple QTL for both foliar and stem resistance to *P. infestans*. The chromosome 5 QTL *lb5b* controlling foliar resistance fractionated into two and five groups of QTL for foliar and stem resistance, respectively (Figure 1). Similarly, the chromosome 11 QTL *lb11b* conferring both foliar and stem resistance fractionated into four and six groups of QTL for foliar and stem resistance, respectively (Figure 2). It is interesting that stem resistance QTL were detected in this chr5 region because the donor parent (*lb5b* NIL) for this sub-NIL population previously exhibited only foliar resistance (Brouwer and St.Clair 2004). Furthermore, two of the QTL groups covering larger genetic regions, *LFRes5-2* and *LFRes11-2*, contain QTL that are minimally overlapping and have widely differing QTL peaks, suggesting that these QTL may fractionate further upon higher-resolution mapping. Our results suggest that the genetic architecture of quantitative resistance to *P. infestans* from *S. habrochaites* is complex, involving multiple loci on both chromosomes 5 and 11. The complexity of quantitative *P. infestans* resistance has also been demonstrated in potato-mapping studies, which have found a high number of resistance QTL from various genetic sources located throughout the potato genome (Danan *et al.* 2011). Other mapping studies have also found that medium-effect QTL (*i.e.* those that account for 20–40%PV) can fractionate into multiple QTL, indicating complex genetic structure (Graham *et al.* 1997; Chen and Tanksley 2004; Gao *et al.* 2004; Lecomte *et al.* 2004; Chen *et al.* 2010; Studer and Doebley 2011).

Typically, QTL mapping studies are performed initially on a global, genome-wide scale using low-density markers spaced at 5–10 cM intervals and population sizes in the low hundreds, resulting in restricted power to detect smaller effect QTL. Subsequently, QTL fractionation can occur during fine- and high-resolution mapping, when larger effect QTL detected in global mapping studies are dissected using higher marker densities and an increased number of recombinants within the QTL region (Mackay *et al.* 2009). The increased level of resolution for a specific region allows closely linked QTL to be separated into their individual effects (Mackay *et al.* 2009). Studer and Doebley (2011) noted that higher-resolution mapping, which localizes a QTL to a smaller genetic region, does not eliminate the potential for additional tightly-linked, undetected QTL in the surrounding region. Thus, QTL fractionation is a clear indication that the trait is controlled by a complex genetic architecture involving multiple loci.

### QTL stability

Effective utilization of favorable QTL alleles in crop breeding requires QTL to be stably expressed across environments and years. Numerous studies in various crop plants describe disease resistance QTL that are stable across multiple environments, including studies examining resistance to Aphanomyces root rot in pea (Hamon *et al.* 2011), blackleg in oilseed rape (Pilet *et al.* 2001), *Fusarium* head blight in wheat (Anderson *et al.* 2001; Yang *et al.* 2003; Zhou *et al.* 2003), late blight in potato (Rauscher *et al.* 2010; Danan *et al.* 2011), leaf rust in barley (Qi *et al.* 2000; Marcel *et al.* 2007), and white mold in bean (Miklas *et al.* 2006; Miklas 2007; Ender *et al.* 2008). Although these studies frequently mapped many (>10) QTL, most detected only a small number (1 to 4) of stable QTL that each explained 14–46%PV and were consistent across environments, years, genetic backgrounds, and occasionally isolates or races of the pathogen, with the exception of Danan *et al.* (2011) and Miklas *et al.* (2006), which reported consensus QTL maps for a given trait.

Our research yielded results similar to these studies in terms of number of stable QTL identified for foliar and stem resistance, and the proportion of phenotypic variation explained by these QTL: four groups of LEAF QTL (*LFRes5-2*, *LFRes11-2*, *LFRes11-3*, and *LFRes11-4*) and four groups of STEM QTL (*STRes5-4*, *STRes11-1*, *STRes11-2*, and *STRes11-6*) were mapped across environments and explained 7–47%PV (Table 4 and Figures 1 and 2). Of the 12 total groups containing 09FD or 10FD QTL, 8 groups contained QTL detected in multiple locations and/or years, indicating stability of QTL expression. Furthermore, 2 of the 7 groups containing GC QTL also contained 09FD and/or 10FD QTL, demonstrating stability of some loci across environments. Significant moderate-to-strong genotypic correlations (*r* ≥ 0.4) across field locations and years also suggest phenotypic stability across environments (Table 3).

QTL stability was also observed in the comparisons between two time periods of data collection within a single growing season (10FD-early and 10FD-late). Significant moderate to strong correlations (*r* = 0.43–0.73) were observed between many of 10FD-early and 10FD-late traits (Table 3). One group of LEAF QTL and one group of STEM QTL contained co-located 10FD-late and 10FD-early QTL (Figures 1 and 2). These significant correlations and co-localizations of QTL suggest that the same QTL are conferring resistance at different times during the season. Few studies have explored consistency of resistance QTL effects throughout a single growing season. Clements *et al.* (2000) examined gray leaf spot resistance QTL in maize detected during two different rating periods (“early” and “late”), and reported QTL that were consistent across the early and late ratings. Resistance QTL determined in our study that were detected in both early and late ratings, and in multiple environments and years, have the most potential to be effective for breeding late blight resistance in tomato (Collard and Mackill 2008; St.Clair 2010).

### QTL × environment interaction and QTL instability

The majority of the studies discussed in relation to QTL stability (see previous section) also reported other QTL, generally of smaller phenotypic effect, that were not consistently identified across environments and genetic backgrounds. Instability of QTL is commonly due to QTL × environment (QTL × E) interactions, wherein the QTL phenotypic effect is influenced by environmental factors, such as water, light, humidity, temperature, soil type, etc. (Mackay *et al.* 2009; St.Clair 2010). Factors such as pathogen isolate and level of disease pressure can also contribute to QTL × E interactions (Pilet *et al.* 2001; Hamon *et al.* 2011). QTL may be unstable due to QTL × genetic background interactions (Collard *et al.* 2005; Bernardo 2008; Collard and Mackill 2008; St.Clair 2010). QTL exhibiting QTL × E and QTL × genetic background interactions are not preferred in breeding due to their ineffectiveness in some environments and genetic backgrounds.

In our research, two and seven LEAF and STEM QTL, respectively, were not co-located with any other QTL of the same trait and allele directionality, suggesting a QTL × E effect (Figures 1 and 2). This QTL instability may have been in part due to QTL × genetic background interactions as three *S. lycopersicum* cultivars were included as recurrent parents. QTL instability was also observed between some 10FD-early and 10FD-late QTL. Although two QTL groups contained QTL detected in both early and late measurements, there were nine groups containing QTL detected in either 10FD-early or 10FD-late, but not in both (Figures 1 and 2). Clements *et al.* (2000) mapped QTL for gray leaf spot resistance in maize that were consistent across two different disease rating periods (early and late), and also reported six QTL that were unique to either early or late measurements. Additionally, LEAF and STEM traits were significantly correlated (*r* = 0.62–0.76) for all isolates tested in GC, but the correlations were only significant for some field experiments (Table 2). This suggests that in the controlled GC environment, foliar and stem disease progression are more co-dependent than in variable field environments.

Smaller-effect QTL can also remain undetected or appear to be unstable due to limited statistical power caused by small sample sizes or low numbers of replications (Collard *et al.* 2005; Mackay *et al.* 2009). For both chr5 and chr11, QTL were more consistently detected across field locations in 10FD than in 09FD (Table 4 and Figures 1 and 2). This difference is likely due to the higher number of replications used per location in 10FD, which resulted in increased accuracy to estimate the genotypic means and increased the power to detect QTL.

### Isolate-specific and isolate-nonspecific QTL

Growth chamber (GC) experiments were used to evaluate whether chr5 and chr11 QTL were associated with resistance to multiple isolates. The GC experiments revealed that resistance QTL for three individually tested isolates did not co-locate (Figures 1 and 2), suggesting that the QTL identified under growth chamber conditions may be primarily isolate-specific. Several late blight resistance studies in potato detected both isolate-specific and isolate-nonspecific QTL (Oberhagemann *et al.* 1999; Villamon *et al.* 2005; Rauscher *et al.* 2010), while Bradshaw *et al.* (2006) reported only isolate-nonspecific QTL, suggesting that the detection of isolate-nonspecific QTL varies depending on the isolates evaluated and the host populations tested.

A GC QTL that was co-located with field QTL (group LFRes11-3, Figure 2) was mapped using isolate p7629, which was not tested in the field experiments, suggesting that under field conditions, some of the QTL detected in the GC may confer resistance to multiple isolates. In contrast, correlations between GC and field genotypic means were either weak (*r* < 0.40) or not significant, suggesting that QTL expression in growth chambers may not be representative of expression under field conditions (Table 3).

QTL conferring resistance to multiple isolates of a pathogen are preferred in breeding because they are more likely to provide a broader spectrum of resistance against pathogen infestations (St.Clair 2010). *P. infestans* has a history of rapidly overcoming isolate-specific resistance genes due to the inherent diversity in the pathogen population and the potential for sexual recombination (Wastie 1991; Fry 2008). The deployment of isolate-nonspecific QTL in cultivars may provide more durable resistance to this pathogen. Numerous reports in potato of QTL that confer resistance to multiple isolates of *P. infestans* [reviewed in Foolad *et al.* (2008)] suggest that potato breeding could benefit from such genetic resources.

### Co-location of LEAF and STEM QTL

The majority of LEAF QTL identified in this study was co-located with STEM QTL, and the alleles contributing to increased disease resistance for each trait were generally donated by the same parent (Table 4 and Figures 1 and 2). This finding implies that the same genes may be controlling resistance to *P. infestans* in different plant organs. To our knowledge, the only previous study in tomato to report *P. infestans* resistance QTL that were associated with both foliar and stem resistance is Brouwer and St.Clair (2004), in which co-located QTL were mapped on chromosomes 4 and 11. Stems are rarely assayed in potato late blight resistance experiments, but several potato studies reported the co-location of QTL for both foliar and tuber resistance to *P. infestans* (Oberhagemann *et al.* 1999; Bradshaw *et al.* 2004, 2006; Park *et al.* 2005; Sliwka *et al.* 2007; Mayton *et al.* 2010). Co-location of foliar and stem resistance QTL implies pleiotropic effects or tightly-linked QTL for each trait, or both. A recent study by Studer and Doebley (2011) detailed the dissection of a single QTL in maize that had demonstrated phenotypic effects on multiple traits for plant architecture and ear morphology, and also contained a 12 kb regulatory region for the *teosinte branched1* (*tb1*) gene. Multiple tightly-linked QTL for ear morphology traits were mapped within the region, including QTL that co-located with the *tb1* control region. In contrast, the only QTL detected for plant architecture traits were co-located with the *tb1* control region, and thus are likely to be pleiotropic effects (Studer and Doebley 2011).

Studies in both tomato (Brouwer and St.Clair 2004) and potato (Oberhagemann *et al.* 1999; Park *et al.* 2005; Simko *et al.* 2006; Sliwka *et al.* 2007; Mayton *et al.* 2010) have reported *P. infestans* resistance QTL that are specific to foliage, stems, or tubers. These results suggest that some of the genes controlling late blight resistance are organ-specific. In support of this hypothesis, research in potato has indicated that phenotypic correlations between foliar and tuber resistance to late blight disease depend on both the *P. infestans* isolate and the host genotype (Wastie 1991; Kirk *et al.* 2001; Foolad *et al.* 2008).

Tightly-linked QTL can be optimal for breeding if the desirable resistance alleles are in coupling phase linkage and there is no detrimental linkage drag associated with the chromosomal region. QTL with pleiotropic effects can also be useful for breeding if the effect on each trait is positive. QTL conferring resistance in a specific organ only are less than ideal, but can still be effectively utilized. For example, QTL can be simultaneously transferred (pyramided) into the same genotype to yield disease resistance in both foliage and stems [reviewed in St.Clair (2010)].

Most sets of co-located LEAF and STEM QTL identified in this research had resistance alleles that were donated by the same parent, although one set of co-located QTL (*LFRes5-2* and *STRes5-3*) had foliar and stem resistance alleles donated by different parents (Figure 1). Oberhagemann *et al.* (1999) reported tightly linked QTL for late blight resistance in different organs (foliage and tubers) of potato, for which the alleles associated with foliage resistance were associated with susceptibility in tubers, and vice versa. They hypothesized that, due to the heterozygous, tetraploid nature of their population, this trait association was caused by differential expression within foliage and tubers of multiple alleles at a single locus (Oberhagemann *et al.* 1999). Our populations of sub-NILs are homozygous and diploid; therefore, the opposing allelic effects in this QTL set is likely caused by pleiotropy and/or tightly linked genes. If pleiotropy is involved, these QTL will not be useful in breeding for *P. infestans* resistance, as the resistance in one organ is associated with susceptibility in another. If the opposing allelic effects are caused by tightly linked genes with the favorable resistance alleles in repulsion phase linkage, a recombination event to separate the causal genes would be required to create a suitable donor parent line in coupling phase linkage for breeding purposes. For example, the *Ph-3* gene in tomato for *P. infestans* resistance and the *Sw-5* gene for *tomato spotted wilt virus* are closely linked but are naturally in repulsion phase linkage in cultivated tomato due to their different parental origins (Robbins *et al.* 2010). Using a large population size and MAS, Robbins *et al.* (2010) identified recombinant individuals with resistance alleles in coupling phase linkage at both genes that are useable as donor parent lines.

### Allele directionality

For the majority of the QTL detected in this study, the alleles conferring resistance were from the *P. infestans* resistant wild donor parent, *S. habrochaites*. This finding is not unexpected given the disease-resistant phenotype of the wild species. In most cases in tomato, the resistant wild species donor parent is the primary source of alleles contributing to disease resistance (Foolad *et al.* 2002; Bai *et al.* 2003; Zhang *et al.* 2003; Brouwer *et al.* 2004; Agrama and Scott 2006). Interestingly, in 4 of the 17 QTL groups, the alleles associated with increased disease resistance were from susceptible cultivated parent *S. lycopersicum* (Figures 1 and 2). The *S. lycopersicum* genome in the pedigree of the two sub-NIL populations is susceptible to late blight disease. Other studies have reported disease resistance conferred by QTL alleles from the susceptible parent (Zhang *et al.* 2003; Brouwer *et al.* 2004; Villamon *et al.* 2005; Anbinder *et al.* 2009; Davis *et al.* 2009). Horticultural trait studies in tomato have revealed instances of favorable alleles contributed by the phenotypically inferior parent (Fulton *et al.* 1997; Bernacchi *et al.* 1998; Truco *et al.* 2000; Frary *et al.* 2004; Stevens *et al.* 2007). A negative trait phenotype of the inferior parent can mask the presence of positive alleles (Zhang *et al.* 2003). Epistatic interactions between the resistance or horticultural trait loci from the donor parent and the genetic background of the recurrent parent may also contribute to the presence of favorable QTL allele effects in the progeny (Mackay *et al.* 2009; St.Clair 2010).

### Comparison with resistance QTL in the Solanaceae

The genomes of tomato, potato, and pepper are closely related, with the same base chromosome number (x = 12) and similar gene content and marker order (Livingstone *et al.* 1999; Grube *et al.* 2000; Rensink *et al.* 2005; Wang *et al.* 2008; Mazourek *et al.* 2009). The macrosynteny between the Solanaceae species facilitates the alignment and comparison of genetic maps and genomic sequences (Bombarely *et al.* 2011). With the exception of Brouwer *et al.* (2004) and Brouwer and St.Clair (2004), to our knowledge there are no other reports of *P. infestans* resistance genes/QTL mapped to either chromosome 5 or 11 in tomato. Therefore, we compared *P. infestans* resistance QTL on chromosomes 5 and 11 of tomato with those mapped in its close relative potato. A number of interesting alignments were found between the QTL detected in our study and the potato meta-QTL consensus map (Danan *et al.* 2011). To do the comparisons, we used the Tomato-Expen 2000 map (Fulton *et al.* 2002) on the Sol Genomics Network (http://solgenomics.net) (Bombarely *et al.* 2011) as an intermediary to align the tomato and potato maps. The tomato-potato QTL comparisons are suggestive but not precise for three reasons: an apparent potato translocation adjacent to the chr11 introgression region; a paucity of common markers across the Tomato-Expen 2000 map and the potato meta-QTL consensus map; and differences in recombination frequencies between our map and the Tomato-Expen 2000 map. Given these caveats, we determined the following putative tomato QTL–potato QTL alignments. On tomato chromosome 5, *P. infestans* resistance QTL *LFRes5-2*, *STRes5-3*, *STRes5-4*, and *STRes5-5* were in a similar location to potato MQTL_2_Late_blight_5. On tomato chromosome 11, the *S. habrochaites* introgressed region encompassed two potato meta-QTL: MQTL_1_Late_blight_11 and MQTL_2_Late_blight_11. Tomato QTL groups *LFRes11-2*, *STRes11-2*, and *STRes11-3* are located within or close to the location of potato MQTL_1_Late_blight_11. Tomato QTL *LFRes11-3*, *STRes11-4*, and *STRes11-5* are in a similar location to potato MQTL_2_Late_blight_11 (Fulton *et al.* 2002; Bombarely *et al.* 2011; Danan *et al.* 2011). The co-location of *P. infestans* resistance QTL between tomato and potato suggests the conservation of gene function during the evolution of these species from a common ancestor.

Interestingly, there are numerous genes and QTL conferring resistance to various pathogens that are co-located across the genomes of Solanaceae species (Grube *et al.* 2000; Gebhardt and Valkonen 2001; Thabius *et al.* 2003). Several of these genes/QTL co-locate to QTL *lb5b* and *lb11b* (Brouwer and St.Clair 2004), which are contained in the two introgressed chromosome regions in the NIL donor parents of our sub-NIL populations. For QTL *lb5b*, the co-located genes and QTL include those conferring resistance to *Xanthomonas campestris* (Yu *et al.* 1995; Yang *et al.* 2005) and *Alternaria solani* (Zhang *et al.* 2003) in tomato, nematodes in potato (Jacobs *et al.* 1996), and *P. capsici* in pepper (Quirin *et al.* 2005). For QTL *lb11b*, the co-located genes and QTL include those conferring resistance to *tomato yellow leaf curl virus* (Anbinder *et al.* 2009) and *Bemisia tabaci* (Momotaz *et al.* 2010) in tomato, and resistance to nematodes (Brown *et al.* 1996), *potato virus Y* (Brigneti *et al.* 1997; Hämäläinen *et al.* 1997), *Globodera pallida* (Tan *et al.* 2009), and *Erwinia carotovora* (Zimnoch-Guzowska *et al.* 2000) in potato. The co-location of tomato *P. infestans* resistance QTL with disease resistance genes and resistance QTL in related Solanaceae species also supports the hypothesis that gene function is conserved within the Solanaceae.

### Breeding applications

When additive QTL with moderate phenotypic effects are verified, effective disease resistance can be attained by simultaneously transferring (or pyramiding) multiple favorable QTL alleles into a single genotype or individual using MAS. As reviewed by St.Clair (2010), there are successful examples of pyramiding QTL alleles for increased disease resistance in crop plants, including resistance to common bacterial blight in bean (Mutlu *et al.* 2005), *Fusarium* head blight in wheat (Wilde *et al.* 2008), root rot and shoot blight in pepper (Thabius *et al.* 2004), and stripe rust in barley (Toojinda *et al.* 1998; Castro *et al.* 2003; Richardson *et al.* 2006). Previously, Brouwer and St.Clair (2004) observed that pyramiding QTL *lb5b* and *lb11b* (contained in the NIL parents of our sub-NIL populations) resulted in a higher level of *P. infestans* resistance (unpublished data). If the combined effects of the resistance QTL mapped in our experiments are predominantly additive, pyramiding QTL from chr5 and chr11 for both stem and leaf resistance may yield genotypes with a higher level of quantitative resistance to *P. infestans*.

The resistance QTL that were most consistently detected across environments and experiments (*i.e.* those with no or minimal QTL × E interactions) represent the most promising candidates to use in late blight resistance breeding programs. Due to the unpredictable nature of environmental conditions, QTL stability across environments is essential for effective breeding of quantitatively inherited traits (Bernardo 2008; Xu and Crouch 2008). The stability exhibited by these QTL suggests that they are more likely to be consistently expressed in a range of environments. Three regions of chr11 offer both foliar and stem resistance: marker intervals TG194 to J1 (encompassing *LFRes11-1* and *STRes11-1*), CT182 to TG147 (encompassing *LFRes11-2*, *LFRes11-3*, *STRes11-3*, and *STRes11-4*), and At5g04590 to TG400 (encompassing *LFRes11-4* and *STRes11-6*) (Figure 2). Of these QTL groups, *LFRes11-3*, *LFRes11-4*, and *STRes11-1* provide the strongest and most stable resistance in terms of the percent phenotypic variation explained by the QTL and consistency of expression across environments and experiments. One region of chr5 confers both foliar and stem resistance: marker intervals TG358 to T0536, which includes both *LFRes5-1* and *STRes5-1* (Figure 1). Although this region has a moderate to strong positive effect on both foliar and stem resistance, it was not consistently detected across environments (Table 4).

Prior to selecting lines to serve as donor parents in a breeding program, in order to maximize selection gain it is necessary to examine the sub-NILs containing these QTL for the potential presence of linkage drag from *S. habrochaites* alleles within the introgression (Charcosset *et al.* 1994; Bernardo 2008; Collard and Mackill 2008; St.Clair 2010). Our previous research suggested that the *lb5b* and *lb11b* QTL appear to be associated with linkage drag for several traits, including maturity, fruit size, and fruit yield (Brouwer and St.Clair 2004). To address the issue of linkage drag, research to fine-map these and other horticultural traits with the sub-NILs for the introgressed chr5 and chr11 regions has been completed recently (J. E. Haggard, E. B. Johnson, and D. A. St.Clair, unpublished results).

To summarize, in this study, we detected the fractionation of two introgressed QTL regions conferring foliar and stem resistance to *P. infestans* on chromosomes 5 and 11 of tomato. In total, 6 and 11 groups of LEAF and STEM QTL were detected, respectively. Of these groups, 4 LEAF groups and 4 STEM groups were stably expressed across environments. Growth chamber experiments testing resistance to multiple isolates suggested that the QTL detected may be isolate-specific. However, correlations among genotypic means from GC and field experiments were low or not significant, suggesting that sub-NIL performance in the growth chamber was not predicative of sub-NIL performance under field conditions. Map-based comparisons suggest that tomato *P. infestans* resistance QTL appear to co-locate with late blight resistance QTL in potato and with other resistance genes and QTL across Solanaceae species. We identified several marker-delimited regions in tomato containing *P. infestans* resistance QTL for both foliar and stem resistance that are promising candidates for use in *P. infestans* resistance breeding programs.

## Supplementary Material

Supporting Information
