# Supplementary material for: Fractionation, Stability, and Isolate-Specificity of QTL for Resistance to Phytophthora infestans in Cultivated Tomato (Solanum lycopersicum)
Source: G3 (Bethesda). 2012 Oct 1;2(10):1145–59. doi: 10.1534/g3.112.003459 (PMC3464107; doi:10.1534/g3.112.003459)
Supplement: Supporting Information [file supp_2_10_1145__index.html]

Supporting Information 

# Fractionation, Stability, and Isolate-Specificity of QTL for Resistance to *Phytophthora infestans* in Cultivated Tomato (*Solanum lycopersicum*)

## Supporting Information for Johnson, Haggard, and St. Clair, 2012

**Files in this Data Supplement:**

- Supporting Information - Tables S1-S3 and Files S1 and S2 (PDF, 355 KB)
- Table S1 - Markers used for chromosome 5 and chromosome 11 introgressed regions (PDF, 76 KB)
- Table S2 - Genotypes and means separation of sub-NILs for chr5 (PDF, 183 KB)
- Table S3 - Genotypes and means separation of sub-NILs for chr11 (PDF, 206 KB)
- File S1 - Supporting Data (.xlsx, 149 KB)
- File S2 - Supporting Data (.xlsx, 862 KB)
