# Supplementary material for: Fractionation, Stability, and Isolate-Specificity of QTL for Resistance to Phytophthora infestans in Cultivated Tomato (Solanum lycopersicum)
Source: G3 (Bethesda). 2012 Oct 1;2(10):1145–59. doi: 10.1534/g3.112.003459 (PMC3464107; doi:10.1534/g3.112.003459)
Supplement: Supporting Information [file supp_2.10.1145_003459SI.pdf]

Files S1 and S2

Supporting Data

Available for download as Excel files at http://www.g3journal.org/lookup/suppl/doi:10.1534/g3.112.003459/-/DC1.

Table S1 Markers used for chromosome 5 and chromosome 11 introgressed regions

All markers are PCR-based and co-dominant unless otherwise noted. Position refers to the position in cM on the genetic maps of the introgressed regions from *S. habrochaites* (not the position on the entire chromosome). In the table, A and B refer to the band sizes for the *S. lycopersicum* and *S. habrochaites* alleles, respectively.

Chr	Marker	Position	Forward Primer	Reverse Primer	Tm[a]	Mg conc[b]	Restriction enzyme	Band sizes (A, B)
**Chr5**	**TG358**	0.0	CAACTTTTCCAGGTTCATTTTCTC	ACACCTACATGCTACTAAGGGGTC	53	2.0	*Hha*I	250, 300
	**At1g10500**	0.0	ACGATTCAATCATCGAGTACAATGG	AGCAGTAAACGATTTTCCACAACCAC	55	1.5	*Hae*III	1000, 800
	**T0536**	0.3	GAGTTCGTAAGACACTGATGG	ATTTCACTTATGCAGGTACG	55	1.5	*Mn*lI	300, 410
	**cLEX-13-G5**	1.5	GCAGCATTTAGGCTCAGAGG	TTCTCAGTGGATCGTGATGG	55	1.5	*Hin*fI	320, 400
	**U221402**	1.8	AAGCCTCCTTGACAAATGCATATAG	AGATATAGCTACAGTGGCAGCTTCATC	55	1.5	*Dde*I	450, 650
	**At3g55800**	2.7	TTTGAAATCAAGCTCATTATTTGG	AGCTGTTCCTCCACAAGAAGCTG	55	1.5	*Dpn*II	200, 320
	**At2g39950 F2/R2**	3.0	TTGGCTGAGTTAACTGGAAT	AATGTGGAGTTCCAACTGAG	55	1.5	*Dpn*II	170 and 180, 370
	**TG23**	5.5	ATGAACTTCCCCCTTAAGTATCTG	CCTTATCCATAGGTTTCCAAAGG	57	2.0	*Ase*I	650, 500
	**At3g17210 F/R3**[c]	5.9	AGCACATTTTGCTAGCAAAGTTCAAAG	CAACATGAACAGGATGAGC	55	1.5	*Dde*I	A 450, B/h 600
	**At3g17210 F2/R**[c]	5.9	GAGTTGTGAAGCACATTTTG	TGATGAAGATTTTCTATGCTCACATCC	55	1.5	*Dde*I	A/h 380 and 500, B null
	**At5g49510**	6.3	AAGCCAGTTTAGAGGTTCTTGTGG	TTCTTTGGGAGTGGTAGCTTGTCG	59	1.5	*Dde*I	310, 250
	**TG60**	7.6	GGTTTGTGGTAGGTTCATCC	TTTTTCTTTGTGAAATGTGC	51	1.5	*Tsp*509I	250, 120 and 150
	**At2g31970**	7.7	TGCGAGGGAGGTGTAGTGCTGG	AACAGAGCTGCAGCAAGACTCTCAC	55	1.5	*Hpa*II	160 and 250, 400
	**At4g12590**	9.4	ACATGGCTATGGATATGATGAAGAAG	ACCCAGAGAAGAAGAAGTTGACCC	55	1.5	*Hin*fI	120, 170
	**T1777**	9.5	AATTCTTCCAGGAATTCACC	TCCAACCATTGAATATTTCC	55	1.5	*Hae*III	700, 800
	**T1541**	9.9	CTCAACTATGGGTGGTGACAATAC	CTTTAGGTTTTTCGGGCTCTTTAG	55	1.5	*Hin*fI	300, 350 and 450
	**TG69**	12.0	TCTTTCTTTTGAAACTTTTGG	CAATGAGGGAACTTCTTGG	51	1.5	*Fok*I	300, 350
	**At3g55360**[d]	12.3	TTTAGTGGTTACACGTAGCGGC	TGCCTCCTGCAGATCAGCAAC	55	2.0	--	750, 400

Table S1, cont.

Chr	Marker	Position	Forward Primer	Reverse Primer	Tm[a]	Mg conc[b]	Restriction enzyme	band sizes (A, B)
**Chr11**	**TG194**	0.0	CAGATGAAAGAAAAGCCAAAAGAG	AATGCTCAGAAGGGAAACATAAAG	57	2.0	*Mn*II	200, 400
	**T0408**	0.7	ATCAGGAACTAGCTCACAGG	AATTCTTCCAGGCTTATTGG	57	2.0	*Mn*II	480, 290 and 300
	**SSR67**[e]	0.7	GCACGAGACCAAGCAGATTA	GGGCCTTTCCTCCAGTAGAC	61	1.5	*Dde*I	850, 390 and 650
	**J1**	1.4	CATCCACCGCTATGTACGTG	CACCACTCACCCATCTTGTG	60	1.5	*Rsa*I	250, 300 and 470
	**TG523**	1.5	TGCAAATGAAGATAAAAGACC	GTGGATAACTCGTTAGTTTCG	53	1.5	*Dpn*II	350, 300
	**At2g22570**	1.5	ACTGAAGAGTGAGATTCCGGTGGAG	TCTGTTCCAGTGATACAATGAGGAGG	55	1.5	*Hin*fI	190, 300
	**At5g16710**	3.0	ACTTGATGAGCTGACAGCTTTCAATG	AGCTTTGGTCCAAGCGACAAATC	55	1.5	*Hae*III	900, 1100
	**U340899**	3.4	GTCCTTGAAGACTTTGATGC	CCCACATTACCAAGATATGG	53	1.5	*Dde*I	450, 300
	**CT182**	4.3	AGTCATTTCATTTGTATTGTAGC	AGGTGGCCAACTTCTTAGG	53	1.5	*Ase*I	500, 370
	**At4g22260**	4.4	TCCTCTAACGGTCTAGAGAAATGGG	AGGAACTCTTGCAATTGTTTCCAGAAC	55	1.5	*Hin*fI	300, 420
	**At3g02870**	4.8	TGAAGCTGCTAAAAAAGCTGGAGAG	ACAAAAGGGAACCCGTGCACAAAG	55	1.5	*Hin*fI	950, 800
	**At1g21690**	5.5	ATGCAGAGCTCTCAGCCATGGG	ACCTACAGCAACAGCAGCAAAGTTC	57	1.5	*Dde*I	400, 280
	**At1g44446**	5.5	AGATCTTGCACACGCACCTTTCAC	TCCTTGCAGACCAGATGCAGGAGTC	55	1.5	*Dde*I	200, 350
	**At1g44790**	5.6	TCGGTTTTATCAAAGGCTATCGTC	TGTTACTGTTCTACCTGGGAATTCTGG	55	1.5	*Ase*I	130 and 160, 290
	**cLEX-4-g10**	6.9	TCCTCCAGAATTATCTGAGC	ACCAAGCTTTTCCTTAAACC	51	1.5	*Hin*fI	450, 230 and 260
	**cLEB-7-LI**	7.5	GAGATCTGCTCTTCTCTTGC	ACAGCTCTGTTGATTCTTCC	59	1.5	*Rsa*I	850, 700
	**TG147**	7.6	ACTGAGGTTAATGATGATGC	GATTAATTGGGAGTATTTGTCC	51	1.5	*Rsa*I	180, 400
	**At4g10050**	8.4	ATCACCCTTCTGCCTTTTCTTC	ATCTGGGATCTGAATGTCATCCTC	55	1.5	*Hin*fI	500, 350
	**At2g14260**	8.4	AGGATCTATACCCCTCTATAGAGCC	TTATTGGGTGAAGTCCCACCTCC	55	1.5	*Dde*I	550, 500
	**At5g04590**	9.0	ATCACCACAGTCCTTGCACAGGG	AGGACAAAGTGGAAAAGCTGGG	58	1.5	*Dde*I	750, 250
	**TG400**	9.4	GCTAATTGAAGTCAAAGAGCACAC	ACCTGTTGTTTGCTTGGTTATATG	55	2.0	*Hae*III	275, 350

[a] Annealing temperature in Celsius.
[b] Mg concentration in mM.
[c] Marker is dominant.
[d] SCAR marker which does not require restriction enzyme digest for polymorphic bands.
[e] To detect a polymorphism between parental genotypes, a restriction enzyme digest of SSR67 was required.

Table S2 Genotypes and means separation of sub-NILs for chr5

Means separation is given for all 09FD_120, GC_120, and 10FD_83 traits. Line Name indicates the name of each control or sub-NIL. The four digit designation for each sub-NIL is preceded by "08GH". The "2010" column indicates which lines were included in the 2010 field experiment. In the Genotype section, "A" indicates homozygosity for the *S. lycopersicum* allele, "B" indicates homozygosity for the *S. habrochaites* allele, and "h" indicates heterozygosity. Trait names are given according to dataset, location or isolate, and trait evaluated (see Materials & Methods). The lowest mean AUDPC for each trait is in bold and underlined.

			Genotype																	Means Separation						
**Line Name**	**Genotype ID**	**2010**	**TG358**	**At10500**	**T0536**	**cLEX13G5**	**U221402**	**At55800**	**At39950**	**TG23**	**At17210**	**At49510**	**TG60**	**At31970**	**At12590**	**T1777**	**T1541**	**TG69**	**At55360**	**09FD_120 Loc1 LEAF**	**09FD_120 Loc2 LEAF**	**10FD-early_83 Loc1&2 LEAF**	**10FD-late_83 Loc1&2 LEAF**	**GC_120 p7629 LEAF**	**GC_120 p10353 LEAF**	**GC_120 Sal10 LEAF**
**Hyp45-A**	**121**	x	A	A	A	A	A	A	A	A	A	A	A	A	A	A	A	A	A	6.02^{ab}	4.70^{a-e}	5.33^{ab}	6.71^{ab}	3.71^{a}	4.51^{a}	4.30^{a}
**Hyp45-B**	**122**		A	A	A	A	A	A	A	A	A	A	A	A	A	A	A	A	A	6.17^{ab}	5.36^{ab}	--	--	--	--	--
**E6203-A**	**123**	x	A	A	A	A	A	A	A	A	A	A	A	A	A	A	A	A	A	6.24^{ab}	4.35^{a-e}	5.26^{a-c}	6.62^{a-e}	--	--	--
**E6203-B**	**124**	x	A	A	A	A	A	A	A	A	A	A	A	A	A	A	A	A	A	6.21^{ab}	4.62^{a-e}	5.05^{a-f}	6.52^{a-g}	3.36^{ab}	4.22^{a}	4.06^{ab}
**E6203-C**	**129**	x	A	A	A	A	A	A	A	A	A	A	A	A	A	A	A	A	A	--	--	4.96^{a-i}	6.44^{a-h}	--	--	--
**E6203-D**	**130**	x	A	A	A	A	A	A	A	A	A	A	A	A	A	A	A	A	A	--	--	5.03^{a-g}	6.32^{a-h}	--	--	--
**LB5-NIL-A**	**125**	x	B	B	B	B	B	B	B	B	B	B	B	B	B	B	B	B	B	5.74^{ab}	4.16^{a-e}	4.35^{h-l}	**5.78^{h}**	**2.42^{c}**	**3.02^{b}**	3.33^{b}
**LB5-NIL-B**	**127**	x	B	B	B	B	B	B	B	B	B	B	B	B	B	B	B	B	B	--	--	**4.20^{l}**	5.89^{f-h}	--	--	--
**7187**	**102**		B	B	A	A	A	A	A	A	A	A	A	A	A	A	A	A	A	5.43^{ab}	3.38^{c-e}	--	--	3.40^{ab}	4.14^{ab}	3.16^{ab}
**7214**	**103**	x	B	B	A	A	A	A	A	A	A	A	A	A	A	A	A	A	A	5.43^{ab}	3.47^{c-e}	4.65^{c-l}	6.52^{a-g}	2.92^{a-c}	3.93^{ab}	3.57^{ab}
**5455**	**52**	x	B	B	B	A	A	A	A	A	A	A	A	A	A	A	A	A	A	5.55^{ab}	4.61^{a-e}	4.91^{a-j}	6.06^{b-h}	3.70^{ab}	4.12^{ab}	4.20^{ab}
**5470**	**53**	x	B	B	B	A	A	A	A	A	A	A	A	A	A	A	A	A	A	6.38^{ab}	4.12^{a-e}	4.59^{d-l}	5.94^{d-h}	3.40^{ab}	4.33^{ab}	4.72^{ab}
**7127**	**100**	x	B	B	B	A	A	A	A	A	A	A	A	A	A	A	A	A	A	5.93^{ab}	4.48^{a-e}	4.50^{e-l}	6.06^{b-h}	3.43^{ab}	3.79^{ab}	3.44^{ab}

Table S2, cont.

Line Name	Genotype ID	2010	Genotype																	Means Separation						
			TG358	At10500	T0536	cLEX13G5	U221402	At55800	At39950	TG23	At17210	At49510	TG60	At31970	At12590	T1777	T1541	TG69	At55360	09FD_120 Loc1 LEAF	09FD_120 Loc2 LEAF	10FD-early_83 Loc1&2 LEAF	10FD-late_83 Loc1&2 LEAF	GC_120 p7629 LEAF	GC_120 p10353 LEAF	GC_120 Sal10 LEAF
**7094**	**99**	x	B	B	B	B	A	A	A	A	A	A	A	A	A	A	A	A	A	6.17^{ab}	3.26^{de}	4.52^{e-l}	5.94^{d-h}	3.32^{a-c}	4.03^{ab}	4.22^{ab}
**7226**	**104**		B	B	B	B	A	A	A	A	A	A	A	A	A	A	A	A	A	5.76^{ab}	3.75^{b-e}	--	--	3.78^{ab}	4.21^{ab}	4.30^{ab}
**6555**	**84**	x	B	B	B	B	B	A	A	A	A	A	A	A	A	A	A	A	A	5.86^{ab}	5.62^{a}	4.70^{a-l}	6.37^{a-h}	3.05^{a-c}	4.13^{ab}	3.34^{ab}
**7444**	**106**		B	B	B	B	B	A	A	A	A	A	A	A	A	A	A	A	A	5.95^{ab}	4.25^{a-e}	--	--	3.51^{ab}	4.23^{ab}	4.27^{ab}
**7471**	**108**	x	B	B	B	B	B	B	A	A	A	A	A	A	A	A	A	A	A	5.86^{ab}	**3.03^{e}**	5.01^{a-g}	6.57^{a-f}	3.34^{a-c}	3.81^{ab}	4.12^{ab}
**5592**	**56**	x	B	B	B	B	B	B	B	A	A	A	A	A	A	A	A	A	A	5.75^{ab}	3.92^{a-e}	4.84^{a-l}	6.27^{a-h}	3.35^{a-c}	4.12^{ab}	3.35^{ab}
**6215**	**72**	x	B	B	B	B	B	B	B	A	A	A	A	A	A	A	A	A	A	6.05^{ab}	3.75^{b-e}	4.62^{c-l}	6.71^{ab}	3.08^{a-c}	3.37^{ab}	4.08^{ab}
**6497**	**83**	x	B	B	B	B	B	B	B	A	A	A	A	A	A	A	A	A	A	5.74^{ab}	4.72^{a-e}	4.39^{g-l}	5.98^{c-h}	3.19^{a-c}	4.28^{ab}	4.52^{ab}
**6979**	**98**	x	B	B	B	B	B	B	B	A	A	A	A	A	A	A	A	A	A	5.86^{ab}	4.44^{a-e}	4.80^{a-l}	6.68^{a-c}	3.44^{ab}	3.66^{ab}	3.87^{ab}
**5553**	**55**		B	B	B	B	B	B	B	B	A	A	A	A	A	A	A	A	A	5.64^{ab}	4.46^{a-e}	--	--	3.38^{ab}	3.71^{ab}	3.68^{ab}
**5873**	**65**	x	B	B	B	B	B	B	B	B	A	A	A	A	A	A	A	A	A	5.69^{ab}	3.99^{a-e}	4.72^{a-l}	6.30^{a-h}	3.41^{ab}	3.80^{ab}	4.08^{ab}
**6941**	**96**	x	B	B	B	B	B	B	B	B	B	A	A	A	A	A	A	A	A	5.82^{ab}	4.20^{a-e}	4.52^{e-l}	6.14^{a-h}	2.55^{bc}	3.21^{ab}	3.25^{ab}
**7459**	**107**	x	B	B	B	B	B	B	B	B	B	A	A	A	A	A	A	A	A	5.71^{ab}	4.39^{a-e}	4.76^{a-l}	6.65^{a-d}	3.15^{a-c}	3.65^{ab}	3.55^{ab}
**6603**	**85**		B	B	B	B	B	B	B	B	B	B	A	A	A	A	A	A	A	5.76^{ab}	3.51^{c-e}	--	--	3.35^{ab}	4.18^{ab}	3.42^{ab}
**6947**	**97**	x	B	B	B	B	B	B	B	B	B	B	A	A	A	A	A	A	A	5.55^{ab}	3.72^{b-e}	4.71^{a-l}	6.33^{a-h}	3.41^{ab}	3.70^{ab}	3.11^{ab}
**6072**	**70**	x	B	B	B	B	B	B	B	B	B	B	B	B	A	A	A	A	A	5.74^{ab}	4.08^{a-e}	4.44^{f-l}	6.40^{a-h}	3.42^{ab}	3.57^{ab}	4.12^{ab}
**6927**	**95**	x	B	B	B	B	B	B	B	B	B	B	B	B	A	A	A	A	A	5.86^{ab}	4.22^{a-e}	4.83^{a-l}	6.55^{a-f}	2.73^{a-c}	3.84^{ab}	4.12^{ab}
**7374**	**105**	x	B	B	B	B	B	B	B	B	B	B	B	B	A	A	A	A	A	5.71^{ab}	3.79^{b-e}	4.39^{g-l}	6.17^{a-h}	3.43^{ab}	4.22^{ab}	4.46^{ab}
**6856**	**93**	x	B	B	B	B	B	B	B	B	B	B	B	B	B	B	B	A	A	5.52^{ab}	3.73^{b-e}	4.26^{j-l}	6.02^{b-h}	3.35^{ab}	4.20^{ab}	4.22^{ab}
**6900**	**94**	x	B	B	B	B	B	B	B	B	B	B	B	B	B	B	B	h	A	5.48^{ab}	4.07^{a-e}	4.46^{f-l}	6.44^{a-h}	2.76^{bc}	3.91^{ab}	3.73^{ab}

Table S2, cont.

Line Name	Genotype ID	2010	TG358	At10500	T0536	cLEX13G5	U221402	At55800	At39950	TG23	At17210	At49510	TG60	At31970	At12590	T1777	T1541	TG69	At55360	09FD_120 Loc1 LEAF	09FD_120 Loc2 LEAF	10FD-early_83 Loc1&2 LEAF	10FD-late_83 Loc1&2 LEAF	GC_120 p7629 LEAF	GC_120 p10353 LEAF	GC_120 Sal10 LEAF
			Genotype																	Means Separation						
**6321**	**79**	x	A	A	B	B	B	B	B	B	B	B	B	B	B	B	B	B	B	5.64^{ab}	3.69^{b-e}	4.58^{d-l}	6.33^{a-h}	3.33^{ab}	3.74^{ab}	3.18^{ab}
**6747**	**89**		A	A	B	B	B	B	B	B	B	B	B	B	B	B	B	B	B	5.50^{ab}	4.29^{a-e}	--	--	3.76^{ab}	4.45^{ab}	4.21^{ab}
**6328**	**80**	x	A	A	A	B	B	B	B	B	B	B	B	B	B	B	B	B	B	5.43^{ab}	4.01^{a-e}	4.58^{d-l}	6.63^{a-e}	3.61^{ab}	3.56^{ab}	4.29^{ab}
**6681**	**86**		A	A	A	B	B	B	B	B	B	B	B	B	B	B	B	B	B	6.14^{ab}	4.22^{a-e}	--	--	3.54^{ab}	4.21^{ab}	4.40^{ab}
**6345**	**81**	x	A	A	A	A	B	B	B	B	B	B	B	B	B	B	B	B	B	5.93^{ab}	4.58^{a-e}	4.50^{e-l}	5.94^{d-h}	3.19^{a-c}	4.25^{ab}	3.22^{ab}
**6709**	**87**		A	A	A	A	B	B	B	B	B	B	B	B	B	B	B	B	B	5.76^{ab}	4.10^{a-e}	--	--	3.15^{a-c}	3.88^{ab}	3.53^{ab}
**6288**	**77**	x	A	A	A	A	A	B	B	B	B	B	B	B	B	B	B	B	B	5.50^{ab}	4.22^{a-e}	4.70^{b-l}	6.52^{a-g}	3.25^{a-c}	3.78^{ab}	4.09^{ab}
**5516**	**54**	x	A	A	A	A	A	A	A	B	B	B	B	B	B	B	B	B	B	5.24^{ab}	3.59^{b-e}	4.31^{j-l}	6.35^{a-h}	3.51^{ab}	3.29^{ab}	3.70^{ab}
**6226**	**73**		A	A	A	A	A	A	A	B	B	B	B	B	B	B	B	B	B	5.76^{ab}	4.49^{a-e}	--	--	3.10^{a-c}	3.17^{ab}	4.32^{ab}
**6234**	**74**		A	A	A	A	A	A	A	B	B	B	B	B	B	B	B	B	B	**4.69^{b}**	3.25^{de}	--	--	2.85^{a-c}	4.42^{ab}	3.79^{ab}
**6423**	**82**		A	A	A	A	A	A	A	B	B	B	B	B	B	B	B	B	B	6.00^{ab}	3.98^{a-e}	--	--	3.56^{ab}	3.58^{ab}	4.21^{ab}
**6724**	**88**		A	A	A	A	A	A	A	B	B	B	B	B	B	B	B	B	B	5.17^{ab}	3.96^{a-e}	--	--	3.52^{ab}	3.85^{ab}	3.48^{ab}
**7166**	**101**		A	A	A	A	A	A	A	B	B	B	B	B	B	B	B	B	B	5.33^{ab}	3.93^{a-e}	--	--	3.00^{a-c}	4.23^{ab}	3.11^{ab}
**6261**	**75**	x	A	A	A	A	A	A	A	A	B	B	B	B	B	B	B	B	B	5.00^{ab}	4.12^{a-e}	4.70^{a-l}	6.17^{a-h}	3.07^{a-c}	3.60^{ab}	**2.76^{ab}**
**6782**	**90**		A	A	A	A	A	A	A	A	B	B	B	B	B	B	B	B	B	5.60^{ab}	4.24^{a-e}	--	--	2.96^{a-c}	4.12^{ab}	3.76^{ab}
**5861**	**64**	x	A	A	A	A	A	A	A	A	A	B	B	B	B	B	B	B	B	5.24^{ab}	4.24^{a-e}	4.25^{kl}	6.09^{b-h}	2.95^{a-c}	3.44^{ab}	4.11^{ab}
**6799**	**91**		A	A	A	A	A	A	A	A	A	B	B	B	B	B	B	B	B	5.43^{ab}	3.61^{b-e}	--	--	3.19^{a-c}	4.10^{ab}	3.47^{ab}
**5616**	**58**	x	A	A	A	A	A	A	A	A	A	A	B	B	B	B	B	B	B	5.36^{ab}	4.20^{a-e}	4.32^{i-l}	5.83^{gh}	3.12^{a-c}	4.47^{a}	3.64^{ab}
**5920**	**66**		A	A	A	A	A	A	A	A	A	A	B	B	B	B	B	B	B	5.76^{ab}	4.14^{a-e}	--	--	3.21^{a-c}	4.07^{ab}	3.17^{ab}
**5941**	**67**		A	A	A	A	A	A	A	A	A	A	B	B	B	B	B	B	B	5.40^{ab}	4.46^{a-e}	--	--	3.46^{ab}	4.89^{a}	3.40^{ab}

Table S2, cont.

Line Name	Genotype ID	2010	TG358	At10500	T0536	cLEX13G5	U221402	At55800	At39950	TG23	At17210	At49510	TG60	At31970	At12590	T1777	T1541	TG69	At55360
			**Genotype**																
**5968**	**68**		A	A	A	A	A	A	A	A	A	A	B	B	B	B	B	B	B
**5705**	**59**	x	A	A	A	A	A	A	A	A	A	A	A	A	B	B	B	B	B
**5722**	**60**	x	A	A	A	A	A	A	A	A	A	A	A	A	B	B	B	B	B
**6011**	**69**	x	A	A	A	A	A	A	A	A	A	A	A	A	B	B	B	B	B
**6302**	**78**	x	A	A	A	A	A	A	A	A	A	A	A	A	B	B	B	B	B
**5605**	**57**	x	A	A	A	A	A	A	A	A	A	A	A	A	A	A	B	B	B
**5771**	**62**	x	A	A	A	A	A	A	A	A	A	A	A	A	A	A	A	B	B
**6094**	**71**	x	A	A	A	A	A	A	A	A	A	A	A	A	A	A	A	B	B
**6278**	**76**	x	A	A	A	A	A	A	A	A	A	A	A	A	A	A	A	B	B
**7610**	**109**	x	A	A	A	A	A	A	A	A	A	A	A	A	A	A	A	B	B
**5726**	**61**	x	A	A	A	A	A	A	A	A	A	A	A	A	A	A	A	A	B
**6042**	**63**	x	A	A	A	A	A	A	A	A	A	A	A	A	A	A	A	A	B
**6805**	**92**	x	A	A	A	A	A	A	A	A	A	B	A	A	A	A	A	A	A

Line Name	Genotype ID	09FD_120 Loc1 LEAF	09FD_120 Loc2 LEAF	10FD-early_83 Loc1&2 LEAF	10FD-late_83 Loc1&2 LEAF	GC_120 p7629 LEAF	GC_120 p10353 LEAF	GC_120 Sal10 LEAF
		**Means Separation**						
**5968**	**68**	5.67^{ab}	4.40^{a-e}	--	--	2.94^{a-c}	4.37^{ab}	3.87^{ab}
**5705**	**59**	6.57^{a}	4.82^{a-d}	5.00^{a-h}	6.40^{a-h}	2.91^{a-c}	3.43^{ab}	4.49^{ab}
**5722**	**60**	5.67^{ab}	4.84^{a-d}	4.98^{a-h}	6.49^{a-h}	3.01^{a-c}	3.91^{ab}	4.40^{ab}
**6011**	**69**	6.00^{ab}	4.58^{a-e}	4.66^{c-l}	6.32^{a-h}	3.17^{a-c}	4.46^{ab}	4.61^{ab}
**6302**	**78**	4.69^{b}	5.07^{a-c}	5.13^{a-e}	6.33^{a-h}	2.91^{a-c}	4.04^{ab}	3.77^{ab}
**5605**	**57**	6.40^{a}	5.29^{ab}	5.00^{a-g}	6.60^{a-f}	3.40^{ab}	3.81^{ab}	3.88^{ab}
**5771**	**62**	6.00^{ab}	4.12^{a-e}	4.90^{a-k}	5.92^{e-h}	3.30^{a-c}	3.69^{ab}	4.35^{ab}
**6094**	**71**	6.33^{ab}	4.17^{a-e}	4.77^{a-l}	6.36^{a-h}	3.53^{ab}	4.39^{ab}	3.58^{ab}
**6278**	**76**	6.00^{ab}	3.89^{a-e}	5.34^{ab}	6.62^{a-e}	2.94^{a-c}	3.52^{ab}	3.45^{ab}
**7610**	**109**	6.07^{ab}	4.60^{a-e}	5.19^{a-d}	6.30^{a-h}	3.18^{a-c}	4.42^{ab}	3.68^{ab}
**5726**	**61**	6.00^{ab}	4.39^{a-e}	5.35^{a}	6.84^{a}	3.08^{a-c}	3.66^{ab}	3.77^{ab}
**6042**	**63**	5.64^{ab}	4.11^{a-e}	4.84^{a-l}	6.25^{a-h}	3.88^{a}	4.34^{ab}	3.96^{ab}
**6805**	**92**	5.55^{ab}	4.30^{a-e}	4.84^{a-l}	6.48^{a-h}	3.20^{a-c}	3.88^{ab}	4.13^{ab}
**Means Separation**		*	*	*	*	*	*	*

Table S2, cont.

Line Name	Genotype ID	2010	Means Separation							
			09FD_120 Loc1&2 STEM	10FD-early_83 Loc1 STEM	10FD-early_83 Loc2 STEM	10FD-late_83 Loc1 STEM	10FD-late_83 Loc2 STEM	GC_120 p7629 STEM	GC_120 p10353 STEM	GC_120 Sal10 STEM
**Hyp45-A**	**121**	x	3.38[a-d]	3.78[a-h]	3.02[a-e]	5.00	4.77[ab]	2.13[a]	2.45[a]	2.20[ab]
**Hyp45-B**	**122**		3.37[a-d]	--	--	--	--	--	--	--
**E6203-A**	**123**	x	2.61[b-j]	3.68[b-h]	2.70[b-i]	4.82	4.55[a-d]	--	--	--
**E6203-B**	**124**	x	2.64[b-j]	3.78[a-h]	2.70[b-i]	4.81	4.25[a-f]	1.62[a-c]	2.10[ab]	1.97[ab]
**E6203-C**	**129**	x	--	3.64[b-h]	2.75[b-i]	4.82	4.61[a-c]	--	--	--
**E6203-D**	**130**	x	--	3.56[b-h]	2.38[b-i]	4.56	4.30[a-f]	--	--	--
**LB5-NIL-A**	**125**	x	3.35[a-e]	3.86[a-e]	3.10[a-d]	5.00	4.23[a-f]	1.37[bc]	1.61[b]	1.96[ab]
**LB5-NIL-B**	**127**	x	--	3.90[a-d]	3.30[ab]	4.82	4.23[a-f]	--	--	--
**7187**	**102**		2.39[f-j]	--	--	--	--	1.60[a-c]	2.48[ab]	1.03[b]
**7214**	**103**	x	2.82[b-j]	3.23[f-i]	1.80[hi]	4.78	3.66[d-f]	1.27[a-c]	2.60[ab]	2.48[ab]
**5455**	**52**	x	3.04[a-h]	3.62[b-h]	2.84[a-h]	4.75	4.11[a-f]	2.09[a-c]	2.24[ab]	2.62[ab]
**5470**	**53**	x	2.59[b-j]	3.74[a-h]	2.65[b-i]	4.65	3.66[d-f]	1.60[a-c]	2.50[ab]	2.67[ab]
**7127**	**100**	x	3.28[a-f]	3.66[b-h]	3.00[a-f]	4.71	3.79[c-f]	1.84[a-c]	2.63[ab]	2.16[ab]

Table S2, cont.

Line Name	Genotype ID	2010	Means Separation							
			09FD_120 Loc1&2 STEM	10FD-early_83 Loc1 STEM	10FD-early_83 Loc2 STEM	10FD-late_83 Loc1 STEM	10FD-late_83 Loc2 STEM	GC_120 p7629 STEM	GC_120 p10353 STEM	GC_120 Sal10 STEM
**7094**	**99**	x	2.65^{b-j}	3.38^{d-i}	2.73^{b-i}	4.53	3.98^{b-f}	1.48^{a-c}	2.27^{ab}	2.50^{ab}
**7226**	**104**		2.72^{b-j}	--	--	--	--	1.85^{a-c}	2.78^{ab}	2.35^{ab}
**6555**	**84**	x	2.89^{b-j}	3.80^{a-f}	2.48^{b-i}	4.75	4.30^{a-f}	1.53^{a-c}	2.10^{ab}	1.67^{ab}
**7444**	**106**		2.92^{b-j}	--	--	--	--	1.88^{a-c}	2.24^{ab}	2.06^{ab}
**7471**	**108**	x	2.48^{d-j}	3.76^{a-h}	2.40^{b-i}	4.82	3.96^{b-f}	1.86^{a-c}	2.01^{ab}	2.37^{ab}
**5592**	**56**	x	2.42^{e-j}	3.62^{b-h}	2.08^{d-i}	**4.47**	3.86^{c-f}	1.54^{a-c}	2.18^{ab}	1.52^{ab}
**6215**	**72**	x	2.31^{g-j}	3.18^{g-i}	**1.67^{i}**	4.88	3.73^{c-f}	1.61^{a-c}	1.98^{ab}	2.24^{ab}
**6497**	**83**	x	2.83^{b-j}	3.34^{d-i}	2.32^{b-i}	4.75	3.91^{b-f}	1.20^{a-c}	2.45^{a}	2.20^{ab}
**6979**	**98**	x	2.66^{b-j}	3.51^{b-i}	2.53^{b-i}	5.00	4.18^{a-f}	2.12^{a-c}	2.43^{ab}	2.33^{ab}
**5553**	**55**		3.02^{a-i}	--	--	--	--	1.75^{a-c}	2.13^{ab}	2.29^{ab}
**5873**	**65**	x	2.55^{b-j}	3.54^{b-i}	2.48^{b-i}	4.81	3.84^{c-f}	2.04^{a-c}	1.99^{ab}	2.32^{ab}
**6941**	**96**	x	2.63^{b-j}	3.58^{b-h}	1.90^{f-i}	4.75	3.54^{ef}	1.11^{a-c}	1.66^{ab}	1.18^{b}
**7459**	**107**	x	2.50^{c-j}	3.25^{e-i}	2.45^{b-i}	4.75	3.98^{b-f}	1.38^{a-c}	1.79^{ab}	1.69^{ab}
**6603**	**85**		**2.02^{j}**	--	--	--	--	1.82^{a-c}	2.64^{ab}	1.59^{ab}
**6947**	**97**	x	2.10^{ij}	**2.93^{i}**	2.07^{d-i}	4.77	**3.48^{f}**	1.84^{a-c}	1.81^{ab}	**1.03^{b}**
**6072**	**70**	x	2.76^{b-j}	3.49^{b-i}	1.98^{e-i}	5.00	3.80^{c-f}	1.82^{a-c}	1.65^{ab}	2.09^{ab}
**6927**	**95**	x	2.65^{b-j}	3.44^{c-i}	2.15^{c-i}	4.71	3.93^{b-f}	1.44^{a-c}	1.79^{ab}	2.18^{ab}
**7374**	**105**	x	2.63^{b-j}	3.48^{b-i}	2.80^{b-h}	4.75	3.91^{b-f}	2.05^{a-c}	2.53^{ab}	2.39^{ab}
**6856**	**93**	x	3.29^{a-f}	3.59^{b-h}	2.58^{b-i}	4.71	3.96^{b-f}	1.91^{a-c}	2.59^{ab}	2.26^{ab}
**6900**	**94**	x	3.22^{a-g}	3.48^{b-i}	2.57^{b-i}	4.94	3.79^{c-f}	1.64^{a-c}	2.41^{ab}	1.83^{ab}

Table S2, cont.

Line Name	Genotype ID	2010	Means Separation							
			09FD_120 Loc1&2 STEM	10FD-early_83 Loc1 STEM	10FD-early_83 Loc2 STEM	10FD-late_83 Loc1 STEM	10FD-late_83 Loc2 STEM	GC_120 p7629 STEM	GC_120 p10353 STEM	GC_120 Sal10 STEM
**6321**	**79**	x	3.42^{a-c}	4.08^{ab}	3.15^{a-d}	5.00	4.46^{a-d}	2.51^{ab}	1.88^{ab}	1.89^{ab}
**6747**	**89**		2.90^{b-j}	--	--	--	--	1.92^{a-c}	2.53^{ab}	1.94^{ab}
**6328**	**80**	x	2.84^{b-j}	3.58^{b-h}	2.57^{b-i}	4.83	4.29^{a-f}	1.59^{a-c}	1.97^{ab}	2.61^{ab}
**6681**	**86**		2.74^{b-j}	--	--	--	--	1.99^{a-c}	2.39^{ab}	2.34^{ab}
**6345**	**81**	x	2.97^{a-i}	3.66^{b-h}	2.90^{a-h}	4.65	3.91^{b-f}	1.37^{a-c}	2.18^{ab}	1.40^{ab}
**6709**	**87**		2.93^{b-j}	--	--	--	--	1.95^{a-c}	2.34^{ab}	1.85^{ab}
**6288**	**77**	x	3.43^{ab}	3.79^{a-g}	3.35^{ab}	5.00	4.41^{a-e}	1.79^{a-c}	2.33^{ab}	2.16^{ab}
**5516**	**54**	x	3.17^{a-h}	3.66^{b-h}	2.70^{b-i}	4.75	3.98^{b-f}	1.93^{a-c}	1.58^{ab}	1.93^{ab}
**6226**	**73**		3.12^{a-h}	--	--	--	--	1.55^{a-c}	1.58^{ab}	2.25^{ab}
**6234**	**74**		2.95^{b-i}	--	--	--	--	1.43^{a-c}	2.36^{ab}	2.08^{ab}
**6423**	**82**		2.28^{h-j}	--	--	--	--	1.90^{a-c}	1.86^{ab}	2.28^{ab}
**6724**	**88**		2.74^{b-j}	--	--	--	--	2.35^{ab}	2.25^{ab}	1.45^{ab}
**7166**	**101**		3.00^{a-i}	--	--	--	--	1.52^{a-c}	2.23^{ab}	1.26^{b}
**6261**	**75**	x	2.71^{b-j}	3.84^{a-f}	3.27^{ab}	4.84	4.46^{a-d}	1.30^{a-c}	2.20^{ab}	1.47^{ab}
**6782**	**90**		2.93^{b-j}	--	--	--	--	1.88^{a-c}	2.58^{ab}	2.15^{ab}
**5861**	**64**	x	2.79^{b-j}	3.76^{a-h}	3.13^{a-d}	4.82	3.98^{b-f}	1.45^{a-c}	2.01^{ab}	2.37^{ab}
**6799**	**91**		3.23^{a-g}	--	--	--	--	1.48^{a-c}	2.38^{ab}	2.05^{ab}
**5616**	**58**	x	2.96^{b-i}	4.01^{a-c}	3.09^{a-d}	4.79	4.48^{a-d}	1.86^{a-c}	2.71^{ab}	2.03^{ab}
**5920**	**66**		3.25^{a-f}	--	--	--	--	2.16^{a-c}	2.20^{ab}	2.26^{ab}
**5941**	**67**		3.13^{a-h}	--	--	--	--	2.43^{a}	3.07^{a}	1.74^{ab}

Table S2, cont.

Line Name	Genotype ID	2010	Means Separation							
			09FD_120 Loc1&2 STEM	10FD-early_83 Loc1 STEM	10FD-early_83 Loc2 STEM	10FD-late_83 Loc1 STEM	10FD-late_83 Loc2 STEM	GC_120 p7629 STEM	GC_120 p10353 STEM	GC_120 Sal10 STEM
**5968**	**68**		2.85^{b-j}	--	--	--	--	1.70^{a-c}	2.89^{ab}	1.93^{ab}
**5705**	**59**	x	3.16^{a-h}	3.86^{a-e}	2.80^{b-h}	5.00	4.36^{a-f}	1.18^{a-c}	2.30^{ab}	2.81^{ab}
**5722**	**60**	x	2.27^{h-j}	3.26^{e-i}	2.30^{b-i}	4.69	4.00^{a-f}	**0.96^{c}**	1.87^{ab}	1.71^{ab}
**6011**	**69**	x	3.89^{a}	4.31^{a}	3.92^{a}	5.00	4.89^{a}	1.97^{a-c}	2.75^{ab}	3.43^{a}
**6302**	**78**	x	2.55^{b-j}	3.82^{a-f}	3.22^{a-c}	4.93	4.63^{a-c}	1.83^{a-c}	2.48^{ab}	1.98^{ab}
**5605**	**57**	x	2.80^{b-j}	3.72^{a-h}	2.98^{a-f}	4.94	4.36^{a-f}	1.80^{a-c}	2.10^{ab}	1.73^{ab}
**5771**	**62**	x	3.00^{a-i}	3.84^{a-f}	2.98^{a-f}	4.88	4.11^{a-f}	1.63^{a-c}	1.84^{ab}	2.63^{ab}
**6094**	**71**	x	2.82^{b-j}	3.76^{a-h}	3.00^{a-f}	4.72	4.09^{a-f}	1.85^{a-c}	2.23^{ab}	1.89^{ab}
**6278**	**76**	x	2.96^{a-i}	3.66^{b-h}	2.52^{b-i}	4.88	4.61^{a-c}	1.76^{a-c}	1.94^{ab}	1.75^{ab}
**7610**	**109**	x	2.94^{b-j}	3.66^{b-h}	3.00^{a-f}	5.00	4.09^{a-f}	1.37^{a-c}	2.32^{ab}	1.92^{ab}
**5726**	**61**	x	2.30^{h-j}	3.18^{hi}	1.83^{g-i}	4.93	3.77^{c-f}	1.43^{a-c}	**1.36^{ab}**	1.95^{ab}
**6042**	**63**	x	2.70^{b-j}	3.86^{a-e}	2.47^{b-i}	4.75	4.27^{a-f}	2.45^{a}	2.65^{ab}	2.04^{ab}
**6805**	**92**	x	2.80^{b-j}	3.84^{a-f}	2.93^{a-g}	4.74	4.41^{a-e}	1.43^{a-c}	2.04^{ab}	1.92^{ab}
**Means Separation**			*	*	*	ns	*	*	*	*

[a] In Proc MIXED in SAS, means separation are calculated using both the difference between means and the number of replications for each entry. Therefore, letter rankings are not always contiguous.
A double dash (--) indicates that the line was not included in the experiment.
An asterisk (*) indicates significance at $P \leq 0.05$ and "ns" indicates not significant.

Table S3 Genotypes and means separation of sub-NILs for chr11

Means separation is given for all 09FD_120, GC_120, and 10FD_83 traits. Line Name indicates the name of each control or sub-NIL. The four digit designation for each sub-NIL is preceded by “08GH”. The “2010” column indicates which lines were included in the 2010 field experiment. In the Genotype section, "A" indicates homozygosity for the *S. lycopersicum* allele and "B" indicates homozygosity for the *S. habrochaites* allele. Trait names are given according to dataset, location or isolate, and trait evaluated. The lowest mean AUDPC for each trait is in bold and underlined.

			Genotype																					Means Separation			
Line Name	Genotype ID	2010	TG194	T0408	SSR67	J1	At22570	TG523	At16710	U340899	CT182	At22260	At02870	At44446	At21690	At44790	cLEX4G10	cLEB7LI	TG147	At14260	At10050	At04590	TG400	09FD_120 Loc1&2 LEAF	10FD-early_83 Loc1&2 LEAF	10FD-late_83 Loc1 LEAF	10FD-late_83 Loc2 LEAF
**Hyp45-A**	**121**	x	A	A	A	A	A	A	A	A	A	A	A	A	A	A	A	A	A	A	A	A	A	5.54^{a}	5.37^{a}	6.70^{a-d}	6.71^{ab}
**Hyp45-B**	**122**		A	A	A	A	A	A	A	A	A	A	A	A	A	A	A	A	A	A	A	A	A	5.89^{a}	--	--	--
**E6203-A**	**123**	x	A	A	A	A	A	A	A	A	A	A	A	A	A	A	A	A	A	A	A	A	A	5.55^{a}	5.31^{ab}	6.40^{a-g}	6.89^{a}
**E6203-B**	**124**	x	A	A	A	A	A	A	A	A	A	A	A	A	A	A	A	A	A	A	A	A	A	5.67^{a}	5.10^{a-d}	6.50^{a-f}	6.54^{a-c}
**E6203-C**	**129**	x	A	A	A	A	A	A	A	A	A	A	A	A	A	A	A	A	A	A	A	A	A	--	5.04^{a-d}	6.40^{a-g}	6.48^{a-c}
**E6203-D**	**130**	x	A	A	A	A	A	A	A	A	A	A	A	A	A	A	A	A	A	A	A	A	A	--	5.07^{a-d}	6.00^{b-g}	6.71^{ab}
**LB11-NIL-A**	**126**	x	B	B	B	B	B	B	B	B	B	B	B	B	B	B	B	B	B	B	B	B	B	4.95^{ab}	4.35^{f-h}	5.90^{c-g}	6.05^{a-c}
**LB11-NIL-B**	**128**	x	B	B	B	B	B	B	B	B	B	B	B	B	B	B	B	B	B	B	B	B	B	--	**<u>4.15^{h}</u>**	5.60^{fg}	**<u>5.52^{c}</u>**
**3951**	**9**	x	B	A	A	A	A	A	A	A	A	A	A	A	A	A	A	A	A	A	A	A	A	5.45^{ab}	4.62^{c-h}	6.40^{a-g}	5.95^{a-c}
**5103**	**42**		B	A	A	A	A	A	A	A	A	A	A	A	A	A	A	A	A	A	A	A	A	5.56^{a}	--	--	--
**4228**	**16**	x	B	B	A	A	A	A	A	A	A	A	A	A	A	A	A	A	A	A	A	A	A	4.76^{ab}	4.89^{a-f}	6.50^{a-f}	6.25^{a-c}
**3999**	**11**	x	B	B	B	A	A	A	A	A	A	A	A	A	A	A	A	A	A	A	A	A	A	4.97^{ab}	4.21^{gh}	6.00^{b-g}	6.18^{a-c}
**4945**	**35**		B	B	B	A	A	A	A	A	A	A	A	A	A	A	A	A	A	A	A	A	A	5.61^{a}	--	--	--
**3892**	**8**	x	B	B	B	B	A	A	A	A	A	A	A	A	A	A	A	A	A	A	A	A	A	5.59^{a}	5.17^{a-c}	6.80^{a-c}	6.54^{a-c}

Table S3, cont.

Line Name	Genotype ID	2010	TG194	T0408	SSR67	J1	At22570	TG523	At16710	U340899	CT182	At22260	At02870	At44446	At21690	At44790	cLEX4G10	cLEB7LI	TG147	At14260	At10050	At04590	TG400	09FD_120 Loc1&2 LEAF	10FD-early_83 Loc1&2 LEAF	10FD-late_83 Loc1 LEAF	10FD-late_83 Loc2 LEAF
			Genotype																					Means Separation			
3969	10	x	B	B	B	B	B	B	A	A	A	A	A	A	A	A	A	A	A	A	A	A	A	5.77^{a}	5.05^{a-d}	6.70^{a-d}	6.71^{ab}
4257	17		B	B	B	B	B	B	A	A	A	A	A	A	A	A	A	A	A	A	A	A	A	5.52^{a}	--	--	--
4940	34		B	B	B	B	B	B	A	A	A	A	A	A	A	A	A	A	A	A	A	A	A	5.62^{a}	--	--	--
7717	110	x	B	B	B	B	B	B	B	A	A	A	A	A	A	A	A	A	A	A	A	A	A	5.69^{a}	4.90^{a-f}	6.40^{a-g}	6.95^{a}
7731	111		B	B	B	B	B	B	B	A	A	A	A	A	A	A	A	A	A	A	A	A	A	5.60^{a}	--	--	--
4265	18	x	B	B	B	B	B	B	B	B	A	A	A	A	A	A	A	A	A	A	A	A	A	5.24^{ab}	4.75^{a-h}	6.40^{a-g}	6.66^{a-c}
4290	19		B	B	B	B	B	B	B	B	A	A	A	A	A	A	A	A	A	A	A	A	A	5.19^{ab}	--	--	--
5224	45		B	B	B	B	B	B	B	B	A	A	A	A	A	A	A	A	A	A	A	A	A	5.77^{a}	--	--	--
7852	116	x	B	B	B	B	B	B	B	B	B	A	A	A	A	A	A	A	A	A	A	A	A	5.26^{ab}	4.62^{c-h}	6.10^{a-g}	6.66^{a-c}
3827	6	x	B	B	B	B	B	B	B	B	B	B	A	A	A	A	A	A	A	A	A	A	A	5.26^{ab}	5.10^{a-d}	6.80^{a-c}	6.59^{a-c}
4659	24		B	B	B	B	B	B	B	B	B	B	A	A	A	A	A	A	A	A	A	A	A	5.39^{ab}	--	--	--
4305	20	x	B	B	B	B	B	B	B	B	B	B	B	A	A	A	A	A	A	A	A	A	A	4.98^{ab}	4.70^{b-h}	6.40^{a-g}	6.36^{a-c}
7743	112		B	B	B	B	B	B	B	B	B	B	B	A	A	A	A	A	A	A	A	A	A	5.46^{ab}	--	--	--
7766	113	x	B	B	B	B	B	B	B	B	B	B	B	B	B	A	A	A	A	A	A	A	A	5.45^{ab}	4.97^{a-f}	6.40^{a-g}	6.89^{a}
7868	117	x	B	B	B	B	B	B	B	B	B	B	B	B	B	A	A	A	A	A	A	A	A	5.38^{ab}	4.77^{a-h}	6.70^{a-d}	5.88^{a-c}
4718	25	x	B	B	B	B	B	B	B	B	B	B	B	B	B	B	A	A	A	A	A	A	A	5.53^{a}	4.84^{a-g}	6.50^{a-f}	6.13^{a-c}
4759	26	x	B	B	B	B	B	B	B	B	B	B	B	B	B	B	A	A	A	A	A	A	A	5.67^{a}	4.90^{a-f}	6.70^{a-d}	6.23^{a-c}
5339	48	x	B	B	B	B	B	B	B	B	B	B	B	B	B	B	B	A	A	A	A	A	A	5.65^{a}	4.90^{a-f}	6.40^{a-g}	6.48^{a-c}
4969	37	x	B	B	B	B	B	B	B	B	B	B	B	B	B	B	B	B	B	A	A	A	A	5.22^{ab}	4.66^{c-h}	6.40^{a-g}	5.93^{a-c}
7799	114		B	B	B	B	B	B	B	B	B	B	B	B	B	B	B	B	B	A	A	A	A	4.74^{ab}	--	--	--

Table S3, cont.

			Genotype																				Means Separation			
Line Name	Genotype ID	2010	TG194	T0408	SSR67	J1	At22570	TG523	At16710	U340899	CT182	At22260	At02870	At44446	At21690	At44790	cLEX4G10	cLEB7LI	TG147	At14260	At10050	At04590	TG400	09FD_120 Loc1&2 LEAF	10FD-early_83 Loc1&2 LEAF	10FD-late_83 Loc1 LEAF	10FD-late_83 Loc2 LEAF
**3845**	**7**	x	B	B	B	B	B	B	B	B	B	B	B	B	B	B	B	B	B	B	B	A	A	5.33^{ab}	4.97^{a-f}	6.30^{a-g}	6.54^{a-c}
**4961**	**36**		B	B	B	B	B	B	B	B	B	B	B	B	B	B	B	B	B	B	B	A	A	4.76^{ab}	--	--	--
**7952**	**118**		B	B	B	B	B	B	B	B	B	B	B	B	B	B	B	B	B	B	B	A	A	5.01^{ab}	--	--	--
**5385**	**50**	x	B	B	B	B	B	B	B	B	B	B	B	B	B	B	B	B	B	B	B	B	A	5.37^{ab}	4.60^{c-h}	5.80^{d-g}	6.00^{a-c}
**5422**	**51**		B	B	B	B	B	B	B	B	B	B	B	B	B	B	B	B	B	B	B	B	A	4.81^{ab}	--	--	--
**4993**	**38**	x	A	B	B	B	B	B	B	B	B	B	B	B	B	B	B	B	B	B	B	B	B	5.24^{ab}	4.73^{b-h}	5.70^{e-g}	5.57^{bc}
**5010**	**39**	x	A	B	B	B	B	B	B	B	B	B	B	B	B	B	B	B	B	B	B	B	B	5.15^{ab}	4.65^{c-h}	6.20^{a-g}	5.88^{a-c}
**5015**	**40**		A	B	B	B	B	B	B	B	B	B	B	B	B	B	B	B	B	B	B	B	B	5.36^{ab}	--	--	--
**5055**	**41**	x	A	A	B	B	B	B	B	B	B	B	B	B	B	B	B	B	B	B	B	B	B	4.87^{ab}	4.68^{b-h}	6.00^{b-g}	5.57^{bc}
**4056**	**13**	x	A	A	A	B	B	B	B	B	B	B	B	B	B	B	B	B	B	B	B	B	B	5.50^{ab}	4.97^{a-f}	6.60^{a-e}	6.25^{a-c}
**4558**	**23**		A	A	A	B	B	B	B	B	B	B	B	B	B	B	B	B	B	B	B	B	B	5.28^{ab}	--	--	--
**4018**	**12**	x	A	A	A	A	A	A	B	B	B	B	B	B	B	B	B	B	B	B	B	B	B	4.88^{ab}	4.35^{f-h}	5.90^{c-g}	6.05^{a-c}
**4861**	**29**		A	A	A	A	A	A	B	B	B	B	B	B	B	B	B	B	B	B	B	B	B	4.63^{ab}	--	--	--
**5162**	**43**	x	A	A	A	A	A	A	A	B	B	B	B	B	B	B	B	B	B	B	B	B	B	5.15^{ab}	4.73^{b-h}	6.00^{b-g}	5.89^{a-c}
**7826**	**115**		A	A	A	A	A	A	A	B	B	B	B	B	B	B	B	B	B	B	B	B	B	5.08^{ab}	--	--	--
**4182**	**15**	x	A	A	A	A	A	A	A	A	B	B	B	B	B	B	B	B	B	B	B	B	B	4.83^{ab}	4.56^{c-h}	5.90^{c-g}	6.36^{a-c}
**5186**	**44**		A	A	A	A	A	A	A	A	B	B	B	B	B	B	B	B	B	B	B	B	B	4.92^{ab}	--	--	--
**8032**	**120**	x	A	A	A	A	A	A	A	A	A	B	B	B	B	B	B	B	B	B	B	B	B	**4.04^{b}**	4.36^{e-h}	5.70^{e-g}	6.00^{a-c}
**4442**	**21**	x	A	A	A	A	A	A	A	A	A	A	B	B	B	B	B	B	B	B	B	B	B	5.00^{ab}	5.00^{a-e}	6.00^{b-g}	6.13^{a-c}
**4922**	**32**		A	A	A	A	A	A	A	A	A	A	B	B	B	B	B	B	B	B	B	B	B	5.12^{ab}	--	--	--
**3688**	**1**	x	A	A	A	A	A	A	A	A	A	A	A	B	B	B	B	B	B	B	B	B	B	5.04^{ab}	4.52^{d-h}	5.80^{d-g}	6.00^{a-c}

Table S3, cont.

Line Name	Genotype ID	2010	TG194	T0408	SSR67	J1	At22570	TG523	At16710	U340899	CT182	At22260	At02870	At44446	At21690	At44790	cLEX4G10	cLEB7LI	TG147	At14260	At10050	At04590	TG400	09FD_120 Loc1&2 LEAF	10FD-early_83 Loc1&2 LEAF	10FD-late_83 Loc1 LEAF	10FD-late_83 Loc2 LEAF
			Genotype																					Means Separation			
**4106**	**14**	x	A	A	A	A	A	A	A	A	A	A	A	B	B	B	B	B	B	B	B	B	B	4.93^{ab}	4.16^{h}	5.90^{c-g}	6.00^{a-c}
**4925**	**33**	x	A	A	A	A	A	A	A	A	A	A	A	A	A	B	B	B	B	B	B	B	B	5.19^{ab}	4.93^{a-f}	6.90^{ab}	6.59^{a-c}
**4498**	**22**	x	A	A	A	A	A	A	A	A	A	A	A	A	A	A	B	B	B	B	B	B	B	4.92^{ab}	5.04^{a-d}	6.60^{a-e}	6.30^{a-c}
**3723**	**2**	x	A	A	A	A	A	A	A	A	A	A	A	A	A	A	B	B	B	B	B	B	B	4.88^{ab}	4.34^{f-h}	6.20^{a-g}	6.13^{a-c}
**4794**	**27**	x	A	A	A	A	A	A	A	A	A	A	A	A	A	A	B	B	B	B	B	B	B	5.03^{ab}	4.76^{a-h}	6.10^{a-g}	6.71^{ab}
**4802**	**28**	x	A	A	A	A	A	A	A	A	A	A	A	A	A	A	A	B	B	B	B	B	B	4.93^{ab}	5.03^{a-d}	6.70^{a-d}	5.95^{a-c}
**5245**	**46**	x	A	A	A	A	A	A	A	A	A	A	A	A	A	A	A	B	B	B	B	B	B	5.20^{ab}	4.76^{a-h}	6.30^{a-g}	6.18^{a-c}
**3740**	**3**	x	A	A	A	A	A	A	A	A	A	A	A	A	A	A	A	A	A	B	B	B	B	4.65^{ab}	4.51^{d-h}	6.50^{a-f}	6.30^{a-c}
**5285**	**47**	x	A	A	A	A	A	A	A	A	A	A	A	A	A	A	A	A	A	B	B	B	B	4.93^{ab}	4.73^{b-h}	6.40^{a-g}	6.43^{a-c}
**3765**	**4**	x	A	A	A	A	A	A	A	A	A	A	A	A	A	A	A	A	A	A	A	B	B	4.97^{ab}	4.48^{d-h}	6.30^{a-g}	6.05^{a-c}
**4897**	**31**	x	A	A	A	A	A	A	A	A	A	A	A	A	A	A	A	A	A	A	A	B	B	5.04^{ab}	4.47^{d-h}	<u>**5.50^{g}**</u>	5.82^{a-c}
**5362**	**49**	x	A	A	A	A	A	A	A	A	A	A	A	A	A	A	A	A	A	A	A	B	B	4.82^{ab}	4.82^{a-g}	6.50^{a-f}	6.07^{a-c}
**4877**	**30**	x	A	A	A	A	A	A	A	A	A	A	A	A	A	A	A	A	A	A	A	A	B	5.18^{ab}	4.82^{a-g}	7.00^{a}	6.54^{a-c}
**8009**	**119**	x	A	A	A	A	A	A	A	A	A	A	A	A	A	A	A	A	A	A	A	A	B	4.80^{ab}	4.69^{b-h}	6.60^{a-e}	6.36^{a-c}
**3814**	**5**		A	A	A	A	A	A	A	A	A	A	A	A	A	A	A	A	A	A	A	A	A	5.98^{a}	--	--	--
**Means Separation**																								*	*	*	*

Table S3, cont.

Line Name	Genotype ID	2010	Means Separation												
			GC_120 p7629 LEAF	GC_120 p10353 LEAF	GC_120 Sal10 LEAF	09FD_120 Loc1&2 STEM	10FD-early_83 Loc1 STEM	10FD-early_83 Loc2 STEM	10FD-late_83 Loc1 STEM	10FD-late_83 Loc2 STEM	GC_120 p7629 STEM	GC_120 p10353 STEM	GC-1_120 Sal10 STEM	GC-2_120 Sal10 STEM	GC-3_120 Sal10 STEM
**Hyp45-A**	**121**	x	3.75^{a}	4.45^{a}	4.08	3.38^{ab}	3.78^{b-e}	3.02^{a-d}	4.90^{a}	4.79^{ab}	2.26^{a}	2.45	2.03	2.45	1.77^{ab}
**Hyp45-B**	**122**		--	--	--	3.37^{a-c}	--	--	--	--	--	--	--	--	--
**E6203-A**	**123**	x	--	--	--	2.61^{bc}	3.68^{c-e}	2.70^{b-d}	4.69^{ab}	4.53^{a-d}	--	--	--	--	--
**E6203-B**	**124**	x	3.41^{ab}	4.25^{a-c}	3.87	2.64^{bc}	3.78^{b-e}	2.70^{b-d}	4.56^{ab}	4.18^{a-d}	1.80^{ab}	2.19	1.76	2.22	1.70^{ab}
**E6203-C**	**129**	x	--	--	--	--	3.64^{c-e}	2.75^{b-d}	4.71^{ab}	4.62^{a-d}	--	--	--	--	--
**E6203-D**	**130**	x	--	--	--	--	3.56^{c-e}	2.38^{de}	4.47^{ab}	4.33^{a-d}	--	--	--	--	--
**LB11-NIL-A**	**126**	x	3.15^{b}	3.55^{bc}	3.47	2.75^{a-c}	3.64^{c-e}	2.66^{b-d}	4.21^{ab}	3.88^{b-d}	1.34^{b}	1.77	1.49	2.25	1.66^{b}
**LB11-NIL-B**	**128**	x	--	--	--	--	3.62^{c-e}	2.50^{cd}	**4.07^{b}**	3.79^{cd}	--	--	--	--	--
**3951**	**9**	x	3.83^{ab}	4.80^{a-c}	4.29	2.95^{a-c}	3.59^{c-e}	2.85^{a-d}	4.26^{ab}	3.83^{cd}	1.93^{ab}	2.42	2.39	3.05	1.68^{ab}
**5103**	**42**		3.25^{ab}	3.43^{a-c}	4.62	**2.33^{c}**	--	--	--	--	1.53^{ab}	1.47	2.42	2.84	1.93^{ab}
**4228**	**16**	x	3.00^{ab}	3.75^{a-c}	4.10	2.84^{a-c}	3.41^{c-e}	**1.27^{e}**	4.36^{ab}	3.96^{b-d}	1.90^{ab}	1.79	1.44	3.81	0.86^{b}
**3999**	**11**	x	3.29^{ab}	4.48^{a-c}	4.33	2.90^{a-c}	**3.26^{e}**	2.28^{de}	4.28^{ab}	3.91^{b-d}	1.69^{ab}	2.34	1.53	2.01	1.95^{ab}
**4945**	**35**		3.64^{ab}	4.12^{a-c}	4.16	2.92^{a-c}	--	--	--	--	1.31^{ab}	2.10	2.19	2.25	1.41^{ab}
**3892**	**8**	x	3.21^{ab}	4.43^{a-c}	4.55	2.69^{bc}	3.53^{c-e}	2.10^{de}	4.56^{ab}	4.11^{a-d}	1.70^{ab}	2.41	2.34	2.74	1.78^{ab}

Table S3, cont.

			Means Separation												
Line Name	Genotype ID	2010	GC_120 p7629 LEAF	GC_120 p10353 LEAF	GC_120 Sal10 LEAF	09FD_120 Loc1&2 STEM	10FD-early_83 Loc1 STEM	10FD-early_83 Loc2 STEM	10FD-late_83 Loc1 STEM	10FD-late_83 Loc2 STEM	GC_120 p7629 STEM	GC_120 p10353 STEM	GC-1_120 Sal10 STEM	GC-2_120 Sal10 STEM	GC-3_120 Sal10 STEM
**3969**	**10**	x	3.59^{ab}	3.88^{a-c}	3.44	3.55^{ab}	3.70^{c-e}	2.48^{cd}	4.69^{ab}	4.46^{a-d}	2.14^{ab}	1.77	1.02	2.09	2.32^{ab}
**4257**	**17**		3.62^{ab}	4.43^{a-c}	4.77	3.22^{a-c}	--	--	--	--	2.01^{ab}	2.42	2.83	3.25	2.19^{ab}
**4940**	**34**		3.53^{ab}	4.06^{a-c}	4.15	2.59^{bc}	--	--	--	--	1.55^{ab}	2.39	1.81	2.49	2.32^{ab}
**7717**	**110**	x	3.66^{ab}	3.90^{a-c}	3.38	3.26^{a-c}	3.62^{c-e}	2.77^{b-d}	4.71^{ab}	4.70^{a-c}	2.30^{ac}	2.18	1.61	1.89	2.28^{ab}
**7731**	**111**		3.66^{ab}	4.35^{a-c}	3.90	3.31^{a-c}	--	--	--	--	1.96^{ab}	2.88	1.44	2.29	1.85^{ab}
**4265**	**18**	x	3.26^{ab}	4.05^{a-c}	3.88	3.21^{a-c}	3.59^{c-e}	2.80^{b-d}	4.68^{ab}	4.79^{ab}	1.67^{ab}	2.09	1.82	2.59	1.14^{ab}
**4290**	**19**		3.21^{ab}	4.00^{a-c}	4.53	2.53^{bc}	--	--	--	--	1.62^{ab}	2.02	1.51	3.33	2.25^{ab}
**5224**	**45**		3.30^{ab}	4.24^{a-c}	3.75	2.60^{bc}	--	--	--	--	1.70^{ab}	2.10	1.93	3.29	1.34^{ab}
**7852**	**116**	x	2.94^{ab}	3.55^{a-c}	3.22	3.40^{ab}	3.94^{a-c}	2.70^{b-d}	4.40^{ab}	3.78^{cd}	1.43^{ab}	1.91	0.90	1.49	1.01^{b}
**3827**	**6**	x	3.90^{ab}	4.17^{a-c}	4.57	3.02^{a-c}	3.69^{c-e}	3.02^{a-d}	4.85^{a}	4.79^{ab}	2.07^{ab}	2.29	2.13	3.39	2.25^{ab}
**4659**	**24**		3.20^{ab}	**3.00^{c}**	3.49	2.91^{a-c}	--	--	--	--	1.83^{ab}	**1.20**	2.41	2.86	0.79^{b}
**4305**	**20**	x	3.48^{ab}	4.16^{a-c}	4.58	2.56^{bc}	3.78^{b-e}	2.85^{a-d}	4.52^{ab}	4.13^{a-d}	2.00^{ab}	2.11	2.48	3.78	2.52^{ab}
**7743**	**112**		3.61^{ab}	4.04^{a-c}	4.98	2.98^{a-c}	--	--	--	--	1.97^{ab}	2.22	1.75	2.44	2.32^{ab}
**7766**	**113**	x	3.88^{ab}	4.04^{a-c}	3.73	2.88^{a-c}	3.59^{c-e}	2.30^{de}	4.74^{ab}	4.62^{a-d}	2.18^{ab}	2.12	2.29	2.17	**0.55^{b}**
**7868**	**117**	x	3.27^{ab}	4.03^{a-c}	3.02	2.64^{bc}	3.52^{c-e}	2.63^{cd}	4.41^{ab}	3.86^{b-d}	1.87^{ab}	2.63	0.87	1.58	1.95^{ab}
**4718**	**25**	x	3.26^{ab}	3.79^{a-c}	4.48	2.76^{a-c}	3.71^{c-e}	2.60^{cd}	4.66^{ab}	4.20^{a-d}	1.33^{ab}	1.88	1.54	2.94	1.26^{ab}
**4759**	**26**	x	3.48^{ab}	3.35^{a-c}	2.97	3.41^{ab}	3.59^{c-e}	2.92^{a-d}	4.54^{ab}	4.27^{a-d}	2.14^{ab}	2.03	1.44	1.37	1.04^{ab}
**5339**	**48**	x	3.17^{ab}	4.30^{a-c}	3.53	2.92^{a-c}	3.68^{c-e}	2.55^{cd}	4.60^{ab}	4.34^{a-d}	1.48^{ab}	2.16	1.32	3.48	1.28^{ab}
**4969**	**37**	x	3.02^{ab}	3.30^{a-c}	3.62	3.04^{a-c}	3.36^{de}	2.98^{a-d}	4.58^{ab}	4.29^{a-d}	1.51^{ab}	1.65	1.15	2.36	0.63^{b}
**7799**	**114**		3.31^{ab}	3.83^{a-c}	3.58	3.16^{a-c}	--	--	--	--	1.60^{ab}	2.09	2.34	2.12	0.88^{b}

Table S3, cont.

Line Name	Genotype ID	2010	GC_120 p7629 LEAF	GC_120 p10353 LEAF	GC_120 Sal10 LEAF	09FD_120 Loc1&2 STEM	10FD-early_83 Loc1 STEM	10FD-early_83 Loc2 STEM	10FD-late_83 Loc1 STEM	10FD-late_83 Loc2 STEM	GC_120 p7629 STEM	GC_120 p10353 STEM	GC-1_120 Sal10 STEM	GC-2_120 Sal10 STEM	GC-3_120 Sal10 STEM
			Means Separation												
**3845**	**7**	x	3.23^{ab}	3.94^{a-c}	3.48	3.31^{a-c}	3.74^{c-e}	2.95^{a-d}	4.71^{ab}	4.55^{a-d}	1.50^{ab}	2.10	1.81	1.92	2.14^{ab}
**4961**	**36**		3.18^{ab}	4.14^{a-c}	4.17	2.96^{a-c}	--	--	--	--	1.68^{ab}	2.32	3.36	1.72	1.78^{ab}
**7952**	**118**		3.14^{ab}	4.00^{a-c}	3.41	3.25^{a-c}	--	--	--	--	1.97^{ab}	1.94	2.70	**0.91**	2.28^{ab}
**5385**	**50**	x	2.96^{ab}	4.15^{a-c}	4.06	3.16^{a-c}	3.64^{c-e}	2.65^{b-d}	4.38^{ab}	3.94^{b-d}	1.36^{ab}	2.58	1.85	3.70	1.05^{ab}
**5422**	**51**		3.46^{ab}	4.21^{a-c}	4.37	2.80^{a-c}	--	--	--	--	2.04^{ab}	2.14	2.55	3.45	2.42^{ab}
**4993**	**38**	x	3.19^{ab}	3.93^{a-c}	3.61	3.47^{ab}	3.82^{b-e}	3.82^{ab}	4.74^{ab}	4.71^{a-c}	2.01^{ab}	2.84	2.43	3.33	2.06^{ab}
**5010**	**39**	x	3.64^{ab}	3.67^{a-c}	3.67	2.87^{a-c}	3.64^{c-e}	2.80^{b-d}	4.52^{ab}	4.17^{a-d}	1.30^{ab}	1.20	1.90	1.36	1.60^{ab}
**5015**	**40**		3.59^{ab}	4.94^{ab}	4.09	2.82^{a-c}	--	--	--	--	1.42^{ab}	2.60	1.78	1.90	2.28^{ab}
**5055**	**41**	x	3.31^{ab}	4.03^{a-c}	3.74	3.21^{a-c}	3.78^{b-e}	2.83^{b-d}	4.46^{ab}	3.96^{b-d}	1.64^{ab}	2.43	1.03	2.96	2.17^{ab}
**4056**	**13**	x	3.52^{ab}	4.43^{a-c}	3.88	3.74^{a}	4.42^{a}	4.02^{a}	4.98^{a}	4.95^{a}	1.97^{ab}	2.40	1.89	2.75	2.85^{ab}
**4558**	**23**		3.37^{ab}	4.33^{a-c}	3.91	3.38^{ab}	--	--	--	--	1.88^{ab}	2.32	3.82	2.36	1.08^{ab}
**4018**	**12**	x	3.50^{ab}	4.65^{a-c}	4.14	2.83^{a-c}	3.80^{b-e}	2.95^{a-d}	4.41^{ab}	4.25^{a-d}	1.79^{ab}	2.26	0.94	4.19	2.29^{ab}
**4861**	**29**		3.12^{ab}	3.46^{a-c}	4.24	2.98^{a-c}	--	--	--	--	1.80^{ab}	1.64	1.48	1.89	2.48^{ab}
**5162**	**43**	x	3.67^{ab}	4.09^{a-c}	3.49	3.41^{ab}	4.32^{ab}	3.65^{a-c}	4.71^{ab}	4.52^{a-d}	1.94^{ab}	2.51	2.53	1.47	2.45^{ab}
**7826**	**115**		3.25^{ab}	4.05^{a-c}	3.67	3.52^{ab}	--	--	--	--	1.28^{ab}	2.19	0.83	1.87	2.95^{ab}
**4182**	**15**	x	3.61^{ab}	4.06^{a-c}	4.13	2.93^{a-c}	3.78^{b-e}	2.83^{b-d}	4.40^{ab}	4.15^{a-d}	2.28^{ab}	2.31	2.18	3.13	1.65^{ab}
**5186**	**44**		3.59^{ab}	4.51^{a-c}	4.19	3.22^{a-c}	--	--	--	--	2.07^{ab}	2.80	1.64	3.61	1.79^{ab}
**8032**	**120**	x	3.11^{ab}	3.77^{a-c}	3.05	3.00^{a-c}	3.60^{c-e}	2.05^{de}	4.29^{ab}	3.88^{b-d}	1.54^{ab}	1.85	2.69	1.87	2.33^{ab}
**4442**	**21**	x	3.71^{ab}	4.51^{a-c}	3.8	3.18^{a-c}	3.76^{b-e}	3.07^{a-d}	4.60^{ab}	4.43^{a-d}	2.21^{ab}	2.22	1.14	3.05	3.20^{ab}
**4922**	**32**		3.67^{ab}	4.59^{a-c}	3.63	2.71^{bc}	--	--	--	--	2.04^{ab}	2.25	1.94	2.62	2.22^{ab}
**3688**	**1**	x	3.14^{ab}	4.15^{a-c}	4.46	2.75^{a-c}	3.38^{c-e}	2.73^{b-d}	4.33^{ab}	4.02^{a-d}	**1.25^{bc}**	2.60	2.88	2.32	3.08^{ab}

Table S3, cont.

Line Name	Genotype ID	2010	Means Separation												
			GC_120 p7629 LEAF	GC_120 p10353 LEAF	GC_120 Sal10 LEAF	09FD_120 Loc1&2 STEM	10FD-early_83 Loc1 STEM	10FD-early_83 Loc2 STEM	10FD-late_83 Loc1 STEM	10FD-late_83 Loc2 STEM	GC_120 p7629 STEM	GC_120 p10353 STEM	GC-1_120 Sal10 STEM	GC-2_120 Sal10 STEM	GC-3_120 Sal10 STEM
**4106**	**14**	x	**2.72**^{b}	3.18^{a-c}	3.00	2.73^{a-c}	3.62^{c-e}	2.55^{cd}	4.35^{ab}	3.93^{b-d}	1.29^{bc}	1.33	1.88	2.54	1.44^{ab}
**4925**	**33**	x	3.44^{ab}	4.34^{a-c}	4.06	2.72^{a-c}	3.64^{c-e}	2.95^{a-d}	4.71^{ab}	4.46^{a-d}	1.84^{ab}	2.45	1.92	3.83	1.66^{ab}
**4498**	**22**	x	2.99^{ab}	3.16^{a-c}	3.38	3.16^{a-c}	3.72^{c-e}	2.17^{de}	4.58^{ab}	4.02^{a-d}	1.46^{ab}	1.72	0.62	2.50	1.62^{ab}
**3723**	**2**	x	3.44^{ab}	4.53^{a-c}	3.85	2.87^{a-c}	3.58^{c-e}	2.32^{de}	4.21^{ab}	3.73^{d}	1.78^{ab}	2.70	**0.54**	3.11	2.20^{ab}
**4794**	**27**	x	3.18^{ab}	4.13^{a-c}	4.49	2.84^{a-c}	3.39^{c-e}	2.42^{de}	4.52^{ab}	4.37^{a-d}	1.43^{ab}	2.67	1.69	3.36	2.73^{ab}
**4802**	**28**	x	3.66^{ab}	3.95^{a-c}	4.52	3.37^{ab}	3.90^{a-d}	2.95^{a-d}	4.63^{ab}	4.36^{a-d}	2.34^{ab}	2.59	1.92	3.12	3.71^{a}
**5245**	**46**	x	3.54^{ab}	4.47^{a-c}	3.83	2.95^{a-c}	3.48^{c-e}	2.37^{de}	4.27^{ab}	3.89^{b-d}	2.17^{ab}	2.07	2.57	2.06	1.97^{ab}
**3740**	**3**	x	3.58^{ab}	4.00^{a-c}	3.77	3.00^{a-c}	3.44^{c-e}	2.08^{de}	4.43^{ab}	**3.70**^{d}	2.05^{ab}	2.11	1.94	2.59	2.85^{ab}
**5285**	**47**	x	3.39^{ab}	3.99^{a-c}	4.23	3.27^{a-c}	3.84^{b-d}	3.15^{a-d}	4.60^{ab}	4.32^{a-d}	2.31^{ab}	2.43	1.73	2.89	3.12^{ab}
**3765**	**4**	x	3.42^{ab}	4.08^{a-c}	4.16	3.09^{a-c}	3.76^{b-e}	3.09^{a-d}	4.52^{ab}	4.12^{a-d}	2.05^{ab}	2.20	3.35	3.11	0.72^{b}
**4897**	**31**	x	3.42^{ab}	3.80^{a-c}	4.15	3.17^{a-c}	3.74^{c-e}	3.23^{a-d}	4.32^{ab}	3.98^{b-d}	1.98^{ab}	2.12	1.45	2.18	2.35^{ab}
**5362**	**49**	x	3.70^{ab}	4.02^{a-c}	3.86	3.27^{a-c}	3.66^{c-e}	2.93^{a-d}	4.43^{ab}	3.88^{b-d}	2.31^{ab}	2.12	2.42	3.60	1.03^{b}
**4877**	**30**	x	3.82^{ab}	4.12^{a-c}	3.74	2.93^{a-c}	3.79^{b-e}	2.53^{cd}	4.78^{ab}	4.53^{a-d}	2.05^{ab}	2.46	2.58	2.55	1.20^{ab}
**8009**	**119**	x	3.28^{ab}	4.55^{a-c}	3.72	3.25^{a-c}	3.64^{c-e}	2.78^{b-d}	4.49^{ab}	4.27^{a-d}	1.55^{ab}	2.94	1.47	3.62	2.14^{ab}
**3814**	**5**		3.53^{ab}	3.79^{a-c}	4.31	2.97^{a-c}	--	--	--	--	1.76^{ab}	1.54	1.93	3.18	1.81^{ab}
**Means Separation**			*	*	ns	*	*	*	*	*	*	ns	ns	ns	*

[a] In Proc MIXED in SAS, means separation are calculated using both the difference between means and the number of replications for each entry. Therefore, letter rankings are not always contiguous.

A double dash (--) indicates that the line was not included in the experiment.

An asterisk (*) indicates significance at $P \leq 0.05$ and "ns" indicates not significant.
