# Supplementary material for: Fractionation, Stability, and Isolate-Specificity of QTL for Resistance to Phytophthora infestans in Cultivated Tomato (Solanum lycopersicum)
Source: G3 (Bethesda). 2012 Oct 1;2(10):1145–59. doi: 10.1534/g3.112.003459 (PMC3464107; doi:10.1534/g3.112.003459)
Supplement: Supporting Information [file supp_2.10.1145_TableS1.pdf]

Table S1 Markers used for chromosome 5 and chromosome 11 introgressed regions

All markers are PCR-based and co-dominant unless otherwise noted. Position refers to the position in cM on the genetic maps of the introgressed regions from *S. habrochaites* (not the position on the entire chromosome). In the table, A and B refer to the band sizes for the *S. lycopersicum* and *S. habrochaites* alleles, respectively.

Chr	Marker	Position	Forward Primer	Reverse Primer	Tm[a]	Mg conc[b]	Restriction enzyme	Band sizes (A, B)
**Chr5**	**TG358**	0.0	CAACTTTTCCAGGTTCATTTTCTC	ACACCTACATGCTACTAAGGGGTC	53	2.0	*Hha*I	250, 300
	**At1g10500**	0.0	ACGATTCAATCATCGAGTACAATGG	AGCAGTAAACGATTTTCCACAACCAC	55	1.5	*Hae*III	1000, 800
	**T0536**	0.3	GAGTTCGTAAGACACTGATGG	ATTTCACTTATGCAGGTACG	55	1.5	*Mn*II	300, 410
	**cLEX-13-G5**	1.5	GCAGCATTTAGGCTCAGAGG	TTCTCAGTGGATCGTGATGG	55	1.5	*Hin*fI	320, 400
	**U221402**	1.8	AAGCCTCCTTGACAAATGCATATAG	AGATATAGCTACAGTGGCAGCTTCATC	55	1.5	*Dde*I	450, 650
	**At3g55800**	2.7	TTTGAAATCAAGCTCATTATTTGG	AGCTGTTCCTCCACAAGAAGCTG	55	1.5	*Dpn*II	200, 320
	**At2g39950 F2/R2**	3.0	TTGGCTGAGTTAACTGGAAT	AATGTGGAGTTCCAACTGAG	55	1.5	*Dpn*II	170 and 180, 370
	**TG23**	5.5	ATGAACTTCCCCCTTAAGTATCTG	CCTTATCCATAGGTTTCCAAAGG	57	2.0	*Ase*I	650, 500
	**At3g17210 F/R3[c]**	5.9	AGCACATTTTGCTAGCAAAGTTCAAAG	CAACATGAACAGGATGAGC	55	1.5	*Dde*I	A 450, B/h 600
	**At3g17210 F2/R[c]**	5.9	GAGTTGTGAAGCACATTTTG	TGATGAAGATTTTCTATGCTCACATCC	55	1.5	*Dde*I	A/h 380 and 500, B null
	**At5g49510**	6.3	AAGCCAGTTTAGAGGTTCTTGTGG	TTCTTTGGGAGTGGTAGCTTGTCG	59	1.5	*Dde*I	310, 250
	**TG60**	7.6	GGTTTGTGGTAGGTTCATCC	TTTTTCTTTGTGAAATGTGC	51	1.5	*Tsp*509I	250, 120 and 150
	**At2g31970**	7.7	TGCGAGGGAGGTGTAGTGCTGG	AACAGAGCTGCAGCAAGACTCTCAC	55	1.5	*Hpa*II	160 and 250, 400
	**At4g12590**	9.4	ACATGGCTATGGATATGATGAAGAAG	ACCCAGAGAAGAAGAAGTTGACCC	55	1.5	*Hin*fI	120, 170
	**T1777**	9.5	AATTCTTCCAGGAATTCACC	TCCAACCATTGAATATTTCC	55	1.5	*Hae*III	700, 800
	**T1541**	9.9	CTCAACTATGGGTGGTGACAATAC	CTTTAGGTTTTTCGGGCTCTTTAG	55	1.5	*Hin*fI	300, 350 and 450
	**TG69**	12.0	TCTTTCTTTTGAAACTTTTGG	CAATGAGGGAACTTCTTGG	51	1.5	*Fok*I	300, 350
	**At3g55360[d]**	12.3	TTTAGTGGTTACACGTAGCGGC	TGCCTCCTGCAGATCAGCAAC	55	2.0	--	750, 400

Table S1, cont.

Chr	Marker	Position	Forward Primer	Reverse Primer	Tm[a]	Mg conc[b]	Restriction enzyme	band sizes (A, B)
**Chr11**	**TG194**	0.0	CAGATGAAAGAAAAGCCAAAAGAG	AATGCTCAGAAGGGAAACATAAAG	57	2.0	*Mn*II	200, 400
	**T0408**	0.7	ATCAGGAACTAGCTCACAGG	AATTCTTCCAGGCTTATTGG	57	2.0	*Mn*II	480, 290 and 300
	**SSR67**[e]	0.7	GCACGAGACCAAGCAGATTA	GGGCCTTTCCTCCAGTAGAC	61	1.5	*Dde*I	850, 390 and 650
	**J1**	1.4	CATCCACCGCTATGTACGTG	CACCACTCACCCATCTTGTG	60	1.5	*Rsa*I	250, 300 and 470
	**TG523**	1.5	TGCAAATGAAGATAAAAGACC	GTGGATAACTCGTTAGTTTCG	53	1.5	*Dpn*II	350, 300
	**At2g22570**	1.5	ACTGAAGAGTGAGATTCCGGTGGAG	TCTGTTCCAGTGATACAATGAGGAGG	55	1.5	*Hin*fI	190, 300
	**At5g16710**	3.0	ACTTGATGAGCTGACAGCTTTCAATG	AGCTTTGGTCCAAGCGACAAATC	55	1.5	*Hae*III	900, 1100
	**U340899**	3.4	GTCCTTGAAGACTTTGATGC	CCCACATTACCAAGATATGG	53	1.5	*Dde*I	450, 300
	**CT182**	4.3	AGTCATTTCATTTGTATTGTAGC	AGGTGGCCAACTTCTTAGG	53	1.5	*Ase*I	500, 370
	**At4g22260**	4.4	TCCTCTAACGGTCTAGAGAAATGGG	AGGAACTCTTGCAATTGTTTCCAGAAC	55	1.5	*Hin*fI	300, 420
	**At3g02870**	4.8	TGAAGCTGCTAAAAAAGCTGGAGAG	ACAAAAGGGAACCCGTGCACAAAG	55	1.5	*Hin*fI	950, 800
	**At1g21690**	5.5	ATGCAGAGCTCTCAGCCATGGG	ACCTACAGCAACAGCAGCAAAGTTC	57	1.5	*Dde*I	400, 280
	**At1g44446**	5.5	AGATCTTGCACACGCACCTTTCAC	TCCTTGCAGACCAGATGCAGGAGTC	55	1.5	*Dde*I	200, 350
	**At1g44790**	5.6	TCGGTTTTATCAAAGGCTATCGTC	TGTTACTGTTCTACCTGGGAATTCTGG	55	1.5	*Ase*I	130 and 160, 290
	**cLEX-4-g10**	6.9	TCCTCCAGAATTATCTGAGC	ACCAAGCTTTTCCTTAAACC	51	1.5	*Hin*fI	450, 230 and 260
	**cLEB-7-LI**	7.5	GAGATCTGCTCTTCTCTTGC	ACAGCTCTGTTGATTCTTCC	59	1.5	*Rsa*I	850, 700
	**TG147**	7.6	ACTGAGGTTAATGATGATGC	GATTAATTGGGAGTATTTGTCC	51	1.5	*Rsa*I	180, 400
	**At4g10050**	8.4	ATCACCCTTCTGCCTTTTCTTC	ATCTGGGATCTGAATGTCATCCTC	55	1.5	*Hin*fI	500, 350
	**At2g14260**	8.4	AGGATCTATACCCCTCTATAGAGCC	TTATTGGGTGAAGTCCCACCTCC	55	1.5	*Dde*I	550, 500
	**At5g04590**	9.0	ATCACCACAGTCCTTGCACAGGG	AGGACAAAGTGGAAAAGCTGGG	58	1.5	*Dde*I	750, 250
	**TG400**	9.4	GCTAATTGAAGTCAAAGAGCACAC	ACCTGTTGTTTGCTTGGTTATATG	55	2.0	*Hae*III	275, 350

[a] Annealing temperature in Celsius.
[b] Mg concentration in mM.
[c] Marker is dominant.
[d] SCAR marker which does not require restriction enzyme digest for polymorphic bands.
[e] To detect a polymorphism between parental genotypes, a restriction enzyme digest of SSR67 was required.
