# Supplementary material for: Fractionation, Stability, and Isolate-Specificity of QTL for Resistance to Phytophthora infestans in Cultivated Tomato (Solanum lycopersicum)
Source: G3 (Bethesda). 2012 Oct 1;2(10):1145–59. doi: 10.1534/g3.112.003459 (PMC3464107; doi:10.1534/g3.112.003459)
Supplement: Supporting Information [file supp_2.10.1145_TableS2.pdf]

Table S2   Genotypes and means separation of sub-NILs for chr5

Means separation is given for all 09FD_120, GC_120, and 10FD_83 traits. Line Name indicates the name of each control or sub-NIL. The four digit designation for each sub-NIL is preceded by “08GH”. The “2010” column indicates which lines were included in the 2010 field experiment. In the Genotype section, "A" indicates homozygosity for the *S. lycopersicum* allele, "B" indicates homozygosity for the *S. habrochaites* allele, and "h" indicates heterozygosity. Trait names are given according to dataset, location or isolate, and trait evaluated (see Materials & Methods). The lowest mean AUDPC for each trait is in bold and underlined.

			Genotype																	Means Separation						
**Line Name**	**Genotype ID**	**2010**	**TG358**	**At10500**	**T0536**	**cLEX13G5**	**U221402**	**At55800**	**At39950**	**TG23**	**At17210**	**At49510**	**TG60**	**At31970**	**At12590**	**T1777**	**T1541**	**TG69**	**At55360**	**09FD_120 Loc1 LEAF**	**09FD_120 Loc2 LEAF**	**10FD-early_83 Loc1&2 LEAF**	**10FD-late_83 Loc1&2 LEAF**	**GC_120 p7629 LEAF**	**GC_120 p10353 LEAF**	**GC_120 Sal10 LEAF**
**Hyp45-A**	**121**	x	A	A	A	A	A	A	A	A	A	A	A	A	A	A	A	A	A	6.02^{ab}	4.70^{a-e}	5.33^{ab}	6.71^{ab}	3.71^{a}	4.51^{a}	4.30^{a}
**Hyp45-B**	**122**		A	A	A	A	A	A	A	A	A	A	A	A	A	A	A	A	A	6.17^{ab}	5.36^{ab}	--	--	--	--	--
**E6203-A**	**123**	x	A	A	A	A	A	A	A	A	A	A	A	A	A	A	A	A	A	6.24^{ab}	4.35^{a-e}	5.26^{a-c}	6.62^{a-e}	--	--	--
**E6203-B**	**124**	x	A	A	A	A	A	A	A	A	A	A	A	A	A	A	A	A	A	6.21^{ab}	4.62^{a-e}	5.05^{a-f}	6.52^{a-g}	3.36^{ab}	4.22^{a}	4.06^{ab}
**E6203-C**	**129**	x	A	A	A	A	A	A	A	A	A	A	A	A	A	A	A	A	A	--	--	4.96^{a-i}	6.44^{a-h}	--	--	--
**E6203-D**	**130**	x	A	A	A	A	A	A	A	A	A	A	A	A	A	A	A	A	A	--	--	5.03^{a-g}	6.32^{a-h}	--	--	--
**LB5-NIL-A**	**125**	x	B	B	B	B	B	B	B	B	B	B	B	B	B	B	B	B	B	5.74^{ab}	4.16^{a-e}	4.35^{h-l}	**5.78^{h}**	**2.42^{c}**	**3.02^{b}**	3.33^{b}
**LB5-NIL-B**	**127**	x	B	B	B	B	B	B	B	B	B	B	B	B	B	B	B	B	B	--	--	**4.20^{l}**	5.89^{f-h}	--	--	--
**7187**	**102**		B	B	A	A	A	A	A	A	A	A	A	A	A	A	A	A	A	5.43^{ab}	3.38^{c-e}	--	--	3.40^{ab}	4.14^{ab}	3.16^{ab}
**7214**	**103**	x	B	B	A	A	A	A	A	A	A	A	A	A	A	A	A	A	A	5.43^{ab}	3.47^{c-e}	4.65^{c-l}	6.52^{a-g}	2.92^{a-c}	3.93^{ab}	3.57^{ab}
**5455**	**52**	x	B	B	B	A	A	A	A	A	A	A	A	A	A	A	A	A	A	5.55^{ab}	4.61^{a-e}	4.91^{a-j}	6.06^{b-h}	3.70^{ab}	4.12^{ab}	4.20^{ab}
**5470**	**53**	x	B	B	B	A	A	A	A	A	A	A	A	A	A	A	A	A	A	6.38^{ab}	4.12^{a-e}	4.59^{d-l}	5.94^{d-h}	3.40^{ab}	4.33^{ab}	4.72^{ab}
**7127**	**100**	x	B	B	B	A	A	A	A	A	A	A	A	A	A	A	A	A	A	5.93^{ab}	4.48^{a-e}	4.50^{e-l}	6.06^{b-h}	3.43^{ab}	3.79^{ab}	3.44^{ab}

Table S2, cont.

			Genotype																	Means Separation						
Line Name	Genotype ID	2010	TG358	At10500	T0536	cLEX13G5	U221402	At55800	At39950	TG23	At17210	At49510	TG60	At31970	At12590	T1777	T1541	TG69	At55360	09FD_120 Loc1 LEAF	09FD_120 Loc2 LEAF	10FD-early_83 Loc1&2 LEAF	10FD-late_83 Loc1&2 LEAF	GC_120 p7629 LEAF	GC_120 p10353 LEAF	GC_120 Sal10 LEAF
**7094**	**99**	x	B	B	B	B	A	A	A	A	A	A	A	A	A	A	A	A	A	6.17^{ab}	3.26^{de}	4.52^{e-l}	5.94^{d-h}	3.32^{a-c}	4.03^{ab}	4.22^{ab}
**7226**	**104**		B	B	B	B	A	A	A	A	A	A	A	A	A	A	A	A	A	5.76^{ab}	3.75^{b-e}	--	--	3.78^{ab}	4.21^{ab}	4.30^{ab}
**6555**	**84**	x	B	B	B	B	B	A	A	A	A	A	A	A	A	A	A	A	A	5.86^{ab}	5.62^{a}	4.70^{a-l}	6.37^{a-h}	3.05^{a-c}	4.13^{ab}	3.34^{ab}
**7444**	**106**		B	B	B	B	B	A	A	A	A	A	A	A	A	A	A	A	A	5.95^{ab}	4.25^{a-e}	--	--	3.51^{ab}	4.23^{ab}	4.27^{ab}
**7471**	**108**	x	B	B	B	B	B	B	A	A	A	A	A	A	A	A	A	A	A	5.86^{ab}	<u>**3.03^{e}**</u>	5.01^{a-g}	6.57^{a-f}	3.34^{a-c}	3.81^{ab}	4.12^{ab}
**5592**	**56**	x	B	B	B	B	B	B	B	A	A	A	A	A	A	A	A	A	A	5.75^{ab}	3.92^{a-e}	4.84^{a-l}	6.27^{a-h}	3.35^{a-c}	4.12^{ab}	3.35^{ab}
**6215**	**72**	x	B	B	B	B	B	B	B	A	A	A	A	A	A	A	A	A	A	6.05^{ab}	3.75^{b-e}	4.62^{c-l}	6.71^{ab}	3.08^{a-c}	3.37^{ab}	4.08^{ab}
**6497**	**83**	x	B	B	B	B	B	B	B	A	A	A	A	A	A	A	A	A	A	5.74^{ab}	4.72^{a-e}	4.39^{g-l}	5.98^{c-h}	3.19^{a-c}	4.28^{ab}	4.52^{ab}
**6979**	**98**	x	B	B	B	B	B	B	B	A	A	A	A	A	A	A	A	A	A	5.86^{ab}	4.44^{a-e}	4.80^{a-l}	6.68^{a-c}	3.44^{ab}	3.66^{ab}	3.87^{ab}
**5553**	**55**		B	B	B	B	B	B	B	B	A	A	A	A	A	A	A	A	A	5.64^{ab}	4.46^{a-e}	--	--	3.38^{ab}	3.71^{ab}	3.68^{ab}
**5873**	**65**	x	B	B	B	B	B	B	B	B	A	A	A	A	A	A	A	A	A	5.69^{ab}	3.99^{a-e}	4.72^{a-l}	6.30^{a-h}	3.41^{ab}	3.80^{ab}	4.08^{ab}
**6941**	**96**	x	B	B	B	B	B	B	B	B	B	A	A	A	A	A	A	A	A	5.82^{ab}	4.20^{a-e}	4.52^{e-l}	6.14^{a-h}	2.55^{bc}	3.21^{ab}	3.25^{ab}
**7459**	**107**	x	B	B	B	B	B	B	B	B	B	A	A	A	A	A	A	A	A	5.71^{ab}	4.39^{a-e}	4.76^{a-l}	6.65^{a-d}	3.15^{a-c}	3.65^{ab}	3.55^{ab}
**6603**	**85**		B	B	B	B	B	B	B	B	B	B	A	A	A	A	A	A	A	5.76^{ab}	3.51^{c-e}	--	--	3.35^{ab}	4.18^{ab}	3.42^{ab}
**6947**	**97**	x	B	B	B	B	B	B	B	B	B	B	A	A	A	A	A	A	A	5.55^{ab}	3.72^{b-e}	4.71^{a-l}	6.33^{a-h}	3.41^{ab}	3.70^{ab}	3.11^{ab}
**6072**	**70**	x	B	B	B	B	B	B	B	B	B	B	B	B	A	A	A	A	A	5.74^{ab}	4.08^{a-e}	4.44^{f-l}	6.40^{a-h}	3.42^{ab}	3.57^{ab}	4.12^{ab}
**6927**	**95**	x	B	B	B	B	B	B	B	B	B	B	B	B	A	A	A	A	A	5.86^{ab}	4.22^{a-e}	4.83^{a-l}	6.55^{a-f}	2.73^{a-c}	3.84^{ab}	4.12^{ab}
**7374**	**105**	x	B	B	B	B	B	B	B	B	B	B	B	B	A	A	A	A	A	5.71^{ab}	3.79^{b-e}	4.39^{g-l}	6.17^{a-h}	3.43^{ab}	4.22^{ab}	4.46^{ab}
**6856**	**93**	x	B	B	B	B	B	B	B	B	B	B	B	B	B	B	B	A	A	5.52^{ab}	3.73^{b-e}	4.26^{j-l}	6.02^{b-h}	3.35^{ab}	4.20^{ab}	4.22^{ab}
**6900**	**94**	x	B	B	B	B	B	B	B	B	B	B	B	B	B	B	B	h	A	5.48^{ab}	4.07^{a-e}	4.46^{f-l}	6.44^{a-h}	2.76^{bc}	3.91^{ab}	3.73^{ab}

Table S2, cont.

			Genotype																	Means Separation						
Line Name	Genotype ID	2010	TG358	At10500	T0536	cLEX13G5	U221402	At55800	At39950	TG23	At17210	At49510	TG60	At31970	At12590	T1777	T1541	TG69	At55360	09FD_120 Loc1 LEAF	09FD_120 Loc2 LEAF	10FD-early_83 Loc1&2 LEAF	10FD-late_83 Loc1&2 LEAF	GC_120 p7629 LEAF	GC_120 p10353 LEAF	GC_120 Sal10 LEAF
**6321**	**79**	x	A	A	B	B	B	B	B	B	B	B	B	B	B	B	B	B	B	5.64^{ab}	3.69^{b-e}	4.58^{d-l}	6.33^{a-h}	3.33^{ab}	3.74^{ab}	3.18^{ab}
**6747**	**89**		A	A	B	B	B	B	B	B	B	B	B	B	B	B	B	B	B	5.50^{ab}	4.29^{a-e}	--	--	3.76^{ab}	4.45^{ab}	4.21^{ab}
**6328**	**80**	x	A	A	A	B	B	B	B	B	B	B	B	B	B	B	B	B	B	5.43^{ab}	4.01^{a-e}	4.58^{d-l}	6.63^{a-e}	3.61^{ab}	3.56^{ab}	4.29^{ab}
**6681**	**86**		A	A	A	B	B	B	B	B	B	B	B	B	B	B	B	B	B	6.14^{ab}	4.22^{a-e}	--	--	3.54^{ab}	4.21^{ab}	4.40^{ab}
**6345**	**81**	x	A	A	A	A	B	B	B	B	B	B	B	B	B	B	B	B	B	5.93^{ab}	4.58^{a-e}	4.50^{e-l}	5.94^{d-h}	3.19^{a-c}	4.25^{ab}	3.22^{ab}
**6709**	**87**		A	A	A	A	B	B	B	B	B	B	B	B	B	B	B	B	B	5.76^{ab}	4.10^{a-e}	--	--	3.15^{a-c}	3.88^{ab}	3.53^{ab}
**6288**	**77**	x	A	A	A	A	A	B	B	B	B	B	B	B	B	B	B	B	B	5.50^{ab}	4.22^{a-e}	4.70^{b-l}	6.52^{a-g}	3.25^{a-c}	3.78^{ab}	4.09^{ab}
**5516**	**54**	x	A	A	A	A	A	A	A	B	B	B	B	B	B	B	B	B	B	5.24^{ab}	3.59^{b-e}	4.31^{j-l}	6.35^{a-h}	3.51^{ab}	3.29^{ab}	3.70^{ab}
**6226**	**73**		A	A	A	A	A	A	A	B	B	B	B	B	B	B	B	B	B	5.76^{ab}	4.49^{a-e}	--	--	3.10^{a-c}	3.17^{ab}	4.32^{ab}
**6234**	**74**		A	A	A	A	A	A	A	B	B	B	B	B	B	B	B	B	B	**4.69^{b}**	3.25^{de}	--	--	2.85^{a-c}	4.42^{ab}	3.79^{ab}
**6423**	**82**		A	A	A	A	A	A	A	B	B	B	B	B	B	B	B	B	B	6.00^{ab}	3.98^{a-e}	--	--	3.56^{ab}	3.58^{ab}	4.21^{ab}
**6724**	**88**		A	A	A	A	A	A	A	B	B	B	B	B	B	B	B	B	B	5.17^{ab}	3.96^{a-e}	--	--	3.52^{ab}	3.85^{ab}	3.48^{ab}
**7166**	**101**		A	A	A	A	A	A	A	B	B	B	B	B	B	B	B	B	B	5.33^{ab}	3.93^{a-e}	--	--	3.00^{a-c}	4.23^{ab}	3.11^{ab}
**6261**	**75**	x	A	A	A	A	A	A	A	A	B	B	B	B	B	B	B	B	B	5.00^{ab}	4.12^{a-e}	4.70^{a-l}	6.17^{a-h}	3.07^{a-c}	3.60^{ab}	**2.76^{ab}**
**6782**	**90**		A	A	A	A	A	A	A	A	B	B	B	B	B	B	B	B	B	5.60^{ab}	4.24^{a-e}	--	--	2.96^{a-c}	4.12^{ab}	3.76^{ab}
**5861**	**64**	x	A	A	A	A	A	A	A	A	A	B	B	B	B	B	B	B	B	5.24^{ab}	4.24^{a-e}	4.25^{kl}	6.09^{b-h}	2.95^{a-c}	3.44^{ab}	4.11^{ab}
**6799**	**91**		A	A	A	A	A	A	A	A	A	B	B	B	B	B	B	B	B	5.43^{ab}	3.61^{b-e}	--	--	3.19^{a-c}	4.10^{ab}	3.47^{ab}
**5616**	**58**	x	A	A	A	A	A	A	A	A	A	A	B	B	B	B	B	B	B	5.36^{ab}	4.20^{a-e}	4.32^{i-l}	5.83^{gh}	3.12^{a-c}	4.47^{a}	3.64^{ab}
**5920**	**66**		A	A	A	A	A	A	A	A	A	A	B	B	B	B	B	B	B	5.76^{ab}	4.14^{a-e}	--	--	3.21^{a-c}	4.07^{ab}	3.17^{ab}
**5941**	**67**		A	A	A	A	A	A	A	A	A	A	B	B	B	B	B	B	B	5.40^{ab}	4.46^{a-e}	--	--	3.46^{ab}	4.89^{a}	3.40^{ab}

Table S2, cont.

Line Name	Genotype ID	2010	TG358	At10500	T0536	cLEX13G5	U221402	At55800	At39950	TG23	At17210	At49510	TG60	At31970	At12590	T1777	T1541	TG69	At55360	09FD_120 Loc1 LEAF	09FD_120 Loc2 LEAF	10FD-early_83 Loc1&2 LEAF	10FD-late_83 Loc1&2 LEAF	GC_120 p7629 LEAF	GC_120 p10353 LEAF	GC_120 Sal10 LEAF
			Genotype																	Means Separation						
**5968**	**68**		A	A	A	A	A	A	A	A	A	A	B	B	B	B	B	B	B	5.67^{ab}	4.40^{a-e}	--	--	2.94^{a-c}	4.37^{ab}	3.87^{ab}
**5705**	**59**	x	A	A	A	A	A	A	A	A	A	A	A	A	B	B	B	B	B	6.57^{a}	4.82^{a-d}	5.00^{a-h}	6.40^{a-h}	2.91^{a-c}	3.43^{ab}	4.49^{ab}
**5722**	**60**	x	A	A	A	A	A	A	A	A	A	A	A	A	B	B	B	B	B	5.67^{ab}	4.84^{a-d}	4.98^{a-h}	6.49^{a-h}	3.01^{a-c}	3.91^{ab}	4.40^{ab}
**6011**	**69**	x	A	A	A	A	A	A	A	A	A	A	A	A	B	B	B	B	B	6.00^{ab}	4.58^{a-e}	4.66^{c-l}	6.32^{a-h}	3.17^{a-c}	4.46^{ab}	4.61^{ab}
**6302**	**78**	x	A	A	A	A	A	A	A	A	A	A	A	A	B	B	B	B	B	4.69^{b}	5.07^{a-c}	5.13^{a-e}	6.33^{a-h}	2.91^{a-c}	4.04^{ab}	3.77^{ab}
**5605**	**57**	x	A	A	A	A	A	A	A	A	A	A	A	A	A	A	B	B	B	6.40^{a}	5.29^{ab}	5.00^{a-g}	6.60^{a-f}	3.40^{ab}	3.81^{ab}	3.88^{ab}
**5771**	**62**	x	A	A	A	A	A	A	A	A	A	A	A	A	A	A	A	B	B	6.00^{ab}	4.12^{a-e}	4.90^{a-k}	5.92^{e-h}	3.30^{a-c}	3.69^{ab}	4.35^{ab}
**6094**	**71**	x	A	A	A	A	A	A	A	A	A	A	A	A	A	A	A	B	B	6.33^{ab}	4.17^{a-e}	4.77^{a-l}	6.36^{a-h}	3.53^{ab}	4.39^{ab}	3.58^{ab}
**6278**	**76**	x	A	A	A	A	A	A	A	A	A	A	A	A	A	A	A	B	B	6.00^{ab}	3.89^{a-e}	5.34^{ab}	6.62^{a-e}	2.94^{a-c}	3.52^{ab}	3.45^{ab}
**7610**	**109**	x	A	A	A	A	A	A	A	A	A	A	A	A	A	A	A	B	B	6.07^{ab}	4.60^{a-e}	5.19^{a-d}	6.30^{a-h}	3.18^{a-c}	4.42^{ab}	3.68^{ab}
**5726**	**61**	x	A	A	A	A	A	A	A	A	A	A	A	A	A	A	A	A	B	6.00^{ab}	4.39^{a-e}	5.35^{a}	6.84^{a}	3.08^{a-c}	3.66^{ab}	3.77^{ab}
**6042**	**63**	x	A	A	A	A	A	A	A	A	A	A	A	A	A	A	A	A	B	5.64^{ab}	4.11^{a-e}	4.84^{a-l}	6.25^{a-h}	3.88^{a}	4.34^{ab}	3.96^{ab}
**6805**	**92**	x	A	A	A	A	A	A	A	A	A	B	A	A	A	A	A	A	A	5.55^{ab}	4.30^{a-e}	4.84^{a-l}	6.48^{a-h}	3.20^{a-c}	3.88^{ab}	4.13^{ab}
**Means Separation**																				*	*	*	*	*	*	*

Table S2, cont.

Line Name	Genotype ID	2010	Means Separation							
			09FD_120 Loc1&2 STEM	10FD-early_83 Loc1 STEM	10FD-early_83 Loc2 STEM	10FD-late_83 Loc1 STEM	10FD-late_83 Loc2 STEM	GC_120 p7629 STEM	GC_120 p10353 STEM	GC_120 Sal10 STEM
**Hyp45-A**	**121**	x	3.38^{a-d}	3.78^{a-h}	3.02^{a-e}	5.00	4.77^{ab}	2.13^{a}	2.45^{a}	2.20^{ab}
**Hyp45-B**	**122**		3.37^{a-d}	--	--	--	--	--	--	--
**E6203-A**	**123**	x	2.61^{b-j}	3.68^{b-h}	2.70^{b-i}	4.82	4.55^{a-d}	--	--	--
**E6203-B**	**124**	x	2.64^{b-j}	3.78^{a-h}	2.70^{b-i}	4.81	4.25^{a-f}	1.62^{a-c}	2.10^{ab}	1.97^{ab}
**E6203-C**	**129**	x	--	3.64^{b-h}	2.75^{b-i}	4.82	4.61^{a-c}	--	--	--
**E6203-D**	**130**	x	--	3.56^{b-h}	2.38^{b-i}	4.56	4.30^{a-f}	--	--	--
**LB5-NIL-A**	**125**	x	3.35^{a-e}	3.86^{a-e}	3.10^{a-d}	5.00	4.23^{a-f}	1.37^{bc}	1.61^{b}	1.96^{ab}
**LB5-NIL-B**	**127**	x	--	3.90^{a-d}	3.30^{ab}	4.82	4.23^{a-f}	--	--	--
**7187**	**102**		2.39^{f-j}	--	--	--	--	1.60^{a-c}	2.48^{ab}	1.03^{b}
**7214**	**103**	x	2.82^{b-j}	3.23^{f-i}	1.80^{hi}	4.78	3.66^{d-f}	1.27^{a-c}	2.60^{ab}	2.48^{ab}
**5455**	**52**	x	3.04^{a-h}	3.62^{b-h}	2.84^{a-h}	4.75	4.11^{a-f}	2.09^{a-c}	2.24^{ab}	2.62^{ab}
**5470**	**53**	x	2.59^{b-j}	3.74^{a-h}	2.65^{b-i}	4.65	3.66^{d-f}	1.60^{a-c}	2.50^{ab}	2.67^{ab}
**7127**	**100**	x	3.28^{a-f}	3.66^{b-h}	3.00^{a-f}	4.71	3.79^{c-f}	1.84^{a-c}	2.63^{ab}	2.16^{ab}

Table S2, cont.

Line Name	Genotype ID	2010	Means Separation							
			09FD_120 Loc1&2 STEM	10FD-early_83 Loc1 STEM	10FD-early_83 Loc2 STEM	10FD-late_83 Loc1 STEM	10FD-late_83 Loc2 STEM	GC_120 p7629 STEM	GC_120 p10353 STEM	GC_120 Sal10 STEM
**7094**	**99**	x	2.65^{b-j}	3.38^{d-i}	2.73^{b-i}	4.53	3.98^{b-f}	1.48^{a-c}	2.27^{ab}	2.50^{ab}
**7226**	**104**		2.72^{b-j}	--	--	--	--	1.85^{a-c}	2.78^{ab}	2.35^{ab}
**6555**	**84**	x	2.89^{b-j}	3.80^{a-f}	2.48^{b-i}	4.75	4.30^{a-f}	1.53^{a-c}	2.10^{ab}	1.67^{ab}
**7444**	**106**		2.92^{b-j}	--	--	--	--	1.88^{a-c}	2.24^{ab}	2.06^{ab}
**7471**	**108**	x	2.48^{d-j}	3.76^{a-h}	2.40^{b-i}	4.82	3.96^{b-f}	1.86^{a-c}	2.01^{ab}	2.37^{ab}
**5592**	**56**	x	2.42^{e-j}	3.62^{b-h}	2.08^{d-i}	**4.47**	3.86^{c-f}	1.54^{a-c}	2.18^{ab}	1.52^{ab}
**6215**	**72**	x	2.31^{g-j}	3.18^{g-i}	**1.67^{i}**	4.88	3.73^{c-f}	1.61^{a-c}	1.98^{ab}	2.24^{ab}
**6497**	**83**	x	2.83^{b-j}	3.34^{d-i}	2.32^{b-i}	4.75	3.91^{b-f}	1.20^{a-c}	2.45^{a}	2.20^{ab}
**6979**	**98**	x	2.66^{b-j}	3.51^{b-i}	2.53^{b-i}	5.00	4.18^{a-f}	2.12^{a-c}	2.43^{ab}	2.33^{ab}
**5553**	**55**		3.02^{a-i}	--	--	--	--	1.75^{a-c}	2.13^{ab}	2.29^{ab}
**5873**	**65**	x	2.55^{b-j}	3.54^{b-i}	2.48^{b-i}	4.81	3.84^{c-f}	2.04^{a-c}	1.99^{ab}	2.32^{ab}
**6941**	**96**	x	2.63^{b-j}	3.58^{b-h}	1.90^{f-i}	4.75	3.54^{ef}	1.11^{a-c}	1.66^{ab}	1.18^{b}
**7459**	**107**	x	2.50^{c-j}	3.25^{e-i}	2.45^{b-i}	4.75	3.98^{b-f}	1.38^{a-c}	1.79^{ab}	1.69^{ab}
**6603**	**85**		**2.02^{j}**	--	--	--	--	1.82^{a-c}	2.64^{ab}	1.59^{ab}
**6947**	**97**	x	2.10^{ij}	**2.93^{i}**	2.07^{d-i}	4.77	**3.48^{f}**	1.84^{a-c}	1.81^{ab}	**1.03^{b}**
**6072**	**70**	x	2.76^{b-j}	3.49^{b-i}	1.98^{e-i}	5.00	3.80^{c-f}	1.82^{a-c}	1.65^{ab}	2.09^{ab}
**6927**	**95**	x	2.65^{b-j}	3.44^{c-i}	2.15^{c-i}	4.71	3.93^{b-f}	1.44^{a-c}	1.79^{ab}	2.18^{ab}
**7374**	**105**	x	2.63^{b-j}	3.48^{b-i}	2.80^{b-h}	4.75	3.91^{b-f}	2.05^{a-c}	2.53^{ab}	2.39^{ab}
**6856**	**93**	x	3.29^{a-f}	3.59^{b-h}	2.58^{b-i}	4.71	3.96^{b-f}	1.91^{a-c}	2.59^{ab}	2.26^{ab}
**6900**	**94**	x	3.22^{a-g}	3.48^{b-i}	2.57^{b-i}	4.94	3.79^{c-f}	1.64^{a-c}	2.41^{ab}	1.83^{ab}

Table S2, cont.

Line Name	Genotype ID	2010	09FD_120 Loc1&2 STEM	10FD-early_83 Loc1 STEM	10FD-early_83 Loc2 STEM	10FD-late_83 Loc1 STEM	10FD-late_83 Loc2 STEM	GC_120 p7629 STEM	GC_120 p10353 STEM	GC_120 Sal10 STEM
			Means Separation							
**6321**	**79**	x	3.42^{a-c}	4.08^{ab}	3.15^{a-d}	5.00	4.46^{a-d}	2.51^{ab}	1.88^{ab}	1.89^{ab}
**6747**	**89**		2.90^{b-j}	--	--	--	--	1.92^{a-c}	2.53^{ab}	1.94^{ab}
**6328**	**80**	x	2.84^{b-j}	3.58^{b-h}	2.57^{b-i}	4.83	4.29^{a-f}	1.59^{a-c}	1.97^{ab}	2.61^{ab}
**6681**	**86**		2.74^{b-j}	--	--	--	--	1.99^{a-c}	2.39^{ab}	2.34^{ab}
**6345**	**81**	x	2.97^{a-i}	3.66^{b-h}	2.90^{a-h}	4.65	3.91^{b-f}	1.37^{a-c}	2.18^{ab}	1.40^{ab}
**6709**	**87**		2.93^{b-j}	--	--	--	--	1.95^{a-c}	2.34^{ab}	1.85^{ab}
**6288**	**77**	x	3.43^{ab}	3.79^{a-g}	3.35^{ab}	5.00	4.41^{a-e}	1.79^{a-c}	2.33^{ab}	2.16^{ab}
**5516**	**54**	x	3.17^{a-h}	3.66^{b-h}	2.70^{b-i}	4.75	3.98^{b-f}	1.93^{a-c}	1.58^{ab}	1.93^{ab}
**6226**	**73**		3.12^{a-h}	--	--	--	--	1.55^{a-c}	1.58^{ab}	2.25^{ab}
**6234**	**74**		2.95^{b-i}	--	--	--	--	1.43^{a-c}	2.36^{ab}	2.08^{ab}
**6423**	**82**		2.28^{h-j}	--	--	--	--	1.90^{a-c}	1.86^{ab}	2.28^{ab}
**6724**	**88**		2.74^{b-j}	--	--	--	--	2.35^{ab}	2.25^{ab}	1.45^{ab}
**7166**	**101**		3.00^{a-i}	--	--	--	--	1.52^{a-c}	2.23^{ab}	1.26^{b}
**6261**	**75**	x	2.71^{b-j}	3.84^{a-f}	3.27^{ab}	4.84	4.46^{a-d}	1.30^{a-c}	2.20^{ab}	1.47^{ab}
**6782**	**90**		2.93^{b-j}	--	--	--	--	1.88^{a-c}	2.58^{ab}	2.15^{ab}
**5861**	**64**	x	2.79^{b-j}	3.76^{a-h}	3.13^{a-d}	4.82	3.98^{b-f}	1.45^{a-c}	2.01^{ab}	2.37^{ab}
**6799**	**91**		3.23^{a-g}	--	--	--	--	1.48^{a-c}	2.38^{ab}	2.05^{ab}
**5616**	**58**	x	2.96^{b-i}	4.01^{a-c}	3.09^{a-d}	4.79	4.48^{a-d}	1.86^{a-c}	2.71^{ab}	2.03^{ab}
**5920**	**66**		3.25^{a-f}	--	--	--	--	2.16^{a-c}	2.20^{ab}	2.26^{ab}
**5941**	**67**		3.13^{a-h}	--	--	--	--	2.43^{a}	3.07^{a}	1.74^{ab}

Table S2, cont.

			Means Separation							
**Line Name**	**Genotype ID**	**2010**	**09FD_120 Loc1&2 STEM**	**10FD-early_83 Loc1 STEM**	**10FD-early_83 Loc2 STEM**	**10FD-late_83 Loc1 STEM**	**10FD-late_83 Loc2 STEM**	**GC_120 p7629 STEM**	**GC_120 p10353 STEM**	**GC_120 Sal10 STEM**
**5968**	**68**		2.85^{b-j}	--	--	--	--	1.70^{a-c}	2.89^{ab}	1.93^{ab}
**5705**	**59**	x	3.16^{a-h}	3.86^{a-e}	2.80^{b-h}	5.00	4.36^{a-f}	1.18^{a-c}	2.30^{ab}	2.81^{ab}
**5722**	**60**	x	2.27^{h-j}	3.26^{e-i}	2.30^{b-i}	4.69	4.00^{a-f}	**0.96^{c}**	1.87^{ab}	1.71^{ab}
**6011**	**69**	x	3.89^{a}	4.31^{a}	3.92^{a}	5.00	4.89^{a}	1.97^{a-c}	2.75^{ab}	3.43^{a}
**6302**	**78**	x	2.55^{b-j}	3.82^{a-f}	3.22^{a-c}	4.93	4.63^{a-c}	1.83^{a-c}	2.48^{ab}	1.98^{ab}
**5605**	**57**	x	2.80^{b-j}	3.72^{a-h}	2.98^{a-f}	4.94	4.36^{a-f}	1.80^{a-c}	2.10^{ab}	1.73^{ab}
**5771**	**62**	x	3.00^{a-i}	3.84^{a-f}	2.98^{a-f}	4.88	4.11^{a-f}	1.63^{a-c}	1.84^{ab}	2.63^{ab}
**6094**	**71**	x	2.82^{b-j}	3.76^{a-h}	3.00^{a-f}	4.72	4.09^{a-f}	1.85^{a-c}	2.23^{ab}	1.89^{ab}
**6278**	**76**	x	2.96^{a-i}	3.66^{b-h}	2.52^{b-i}	4.88	4.61^{a-c}	1.76^{a-c}	1.94^{ab}	1.75^{ab}
**7610**	**109**	x	2.94^{b-j}	3.66^{b-h}	3.00^{a-f}	5.00	4.09^{a-f}	1.37^{a-c}	2.32^{ab}	1.92^{ab}
**5726**	**61**	x	2.30^{h-j}	3.18^{hi}	1.83^{g-i}	4.93	3.77^{c-f}	1.43^{a-c}	**1.36^{ab}**	1.95^{ab}
**6042**	**63**	x	2.70^{b-j}	3.86^{a-e}	2.47^{b-i}	4.75	4.27^{a-f}	2.45^{a}	2.65^{ab}	2.04^{ab}
**6805**	**92**	x	2.80^{b-j}	3.84^{a-f}	2.93^{a-g}	4.74	4.41^{a-e}	1.43^{a-c}	2.04^{ab}	1.92^{ab}
**Means Separation**			*	*	*	ns	*	*	*	*

[a] In Proc MIXED in SAS, means separation are calculated using both the difference between means and the number of replications for each entry. Therefore, letter rankings are not always contiguous.

A double dash (--) indicates that the line was not included in the experiment.

An asterisk (*) indicates significance at $P \leq 0.05$ and "ns" indicates not significant.
