# Supplementary material for: Fractionation, Stability, and Isolate-Specificity of QTL for Resistance to Phytophthora infestans in Cultivated Tomato (Solanum lycopersicum)
Source: G3 (Bethesda). 2012 Oct 1;2(10):1145–59. doi: 10.1534/g3.112.003459 (PMC3464107; doi:10.1534/g3.112.003459)
Supplement: Supporting Information [file supp_2.10.1145_TableS3.pdf]

Table S3 Genotypes and means separation of sub-NILs for chr11

Means separation is given for all 09FD_120, GC_120, and 10FD_83 traits. Line Name indicates the name of each control or sub-NIL. The four digit designation for each sub-NIL is preceded by "08GH". The "2010" column indicates which lines were included in the 2010 field experiment. In the Genotype section, "A" indicates homozygosity for the *S. lycopersicum* allele and "B" indicates homozygosity for the *S. habrochaites* allele. Trait names are given according to dataset, location or isolate, and trait evaluated. The lowest mean AUDPC for each trait is in bold and underlined.

			Genotype																					Means Separation			
Line Name	Genotype ID	2010	TG194	T0408	SSR67	J1	At22570	TG523	At16710	U340899	CT182	At22260	At02870	At44446	At21690	At44790	cLEX4G10	cLEB7LI	TG147	At14260	At10050	At04590	TG400	09FD_120 Loc1&2 LEAF	10FD-early_83 Loc1&2 LEAF	10FD-late_83 Loc1 LEAF	10FD-late_83 Loc2 LEAF
**Hyp45-A**	**121**	x	A	A	A	A	A	A	A	A	A	A	A	A	A	A	A	A	A	A	A	A	A	5.54^{a}	5.37^{a}	6.70^{a-d}	6.71^{ab}
**Hyp45-B**	**122**		A	A	A	A	A	A	A	A	A	A	A	A	A	A	A	A	A	A	A	A	A	5.89^{a}	--	--	--
**E6203-A**	**123**	x	A	A	A	A	A	A	A	A	A	A	A	A	A	A	A	A	A	A	A	A	A	5.55^{a}	5.31^{ab}	6.40^{a-g}	6.89^{a}
**E6203-B**	**124**	x	A	A	A	A	A	A	A	A	A	A	A	A	A	A	A	A	A	A	A	A	A	5.67^{a}	5.10^{a-d}	6.50^{a-f}	6.54^{a-c}
**E6203-C**	**129**	x	A	A	A	A	A	A	A	A	A	A	A	A	A	A	A	A	A	A	A	A	A	--	5.04^{a-d}	6.40^{a-g}	6.48^{a-c}
**E6203-D**	**130**	x	A	A	A	A	A	A	A	A	A	A	A	A	A	A	A	A	A	A	A	A	A	--	5.07^{a-d}	6.00^{b-g}	6.71^{ab}
**LB11-NIL-A**	**126**	x	B	B	B	B	B	B	B	B	B	B	B	B	B	B	B	B	B	B	B	B	B	4.95^{ab}	4.35^{f-h}	5.90^{c-g}	6.05^{a-c}
**LB11-NIL-B**	**128**	x	B	B	B	B	B	B	B	B	B	B	B	B	B	B	B	B	B	B	B	B	B	--	**4.15^{h}**	5.60^{fg}	**5.52^{c}**
**3951**	**9**	x	B	A	A	A	A	A	A	A	A	A	A	A	A	A	A	A	A	A	A	A	A	5.45^{ab}	4.62^{c-h}	6.40^{a-g}	5.95^{a-c}
**5103**	**42**		B	A	A	A	A	A	A	A	A	A	A	A	A	A	A	A	A	A	A	A	A	5.56^{a}	--	--	--
**4228**	**16**	x	B	B	A	A	A	A	A	A	A	A	A	A	A	A	A	A	A	A	A	A	A	4.76^{ab}	4.89^{a-f}	6.50^{a-f}	6.25^{a-c}
**3999**	**11**	x	B	B	B	A	A	A	A	A	A	A	A	A	A	A	A	A	A	A	A	A	A	4.97^{ab}	4.21^{gh}	6.00^{b-g}	6.18^{a-c}
**4945**	**35**		B	B	B	A	A	A	A	A	A	A	A	A	A	A	A	A	A	A	A	A	A	5.61^{a}	--	--	--
**3892**	**8**	x	B	B	B	B	A	A	A	A	A	A	A	A	A	A	A	A	A	A	A	A	A	5.59^{a}	5.17^{a-c}	6.80^{a-c}	6.54^{a-c}

Table S3, cont.

Line Name	Genotype ID	2010	TG194	T0408	SSR67	J1	At22570	TG523	At16710	U340899	CT182	At22260	At02870	At44446	At21690	At44790	cLEX4G10	cLEB7LI	TG147	At14260	At10050	At04590	TG400	09FD_120 Loc1&2 LEAF	10FD-early_83 Loc1&2 LEAF	10FD-late_83 Loc1 LEAF	10FD-late_83 Loc2 LEAF
			Genotype																					Means Separation			
3969	10	x	B	B	B	B	B	B	A	A	A	A	A	A	A	A	A	A	A	A	A	A	A	5.77^{a}	5.05^{a-d}	6.70^{a-d}	6.71^{ab}
4257	17		B	B	B	B	B	B	A	A	A	A	A	A	A	A	A	A	A	A	A	A	A	5.52^{a}	--	--	--
4940	34		B	B	B	B	B	B	A	A	A	A	A	A	A	A	A	A	A	A	A	A	A	5.62^{a}	--	--	--
7717	110	x	B	B	B	B	B	B	B	A	A	A	A	A	A	A	A	A	A	A	A	A	A	5.69^{a}	4.90^{a-f}	6.40^{a-g}	6.95^{a}
7731	111		B	B	B	B	B	B	B	A	A	A	A	A	A	A	A	A	A	A	A	A	A	5.60^{a}	--	--	--
4265	18	x	B	B	B	B	B	B	B	B	A	A	A	A	A	A	A	A	A	A	A	A	A	5.24^{ab}	4.75^{a-h}	6.40^{a-g}	6.66^{a-c}
4290	19		B	B	B	B	B	B	B	B	A	A	A	A	A	A	A	A	A	A	A	A	A	5.19^{ab}	--	--	--
5224	45		B	B	B	B	B	B	B	B	A	A	A	A	A	A	A	A	A	A	A	A	A	5.77^{a}	--	--	--
7852	116	x	B	B	B	B	B	B	B	B	B	A	A	A	A	A	A	A	A	A	A	A	A	5.26^{ab}	4.62^{c-h}	6.10^{a-g}	6.66^{a-c}
3827	6	x	B	B	B	B	B	B	B	B	B	B	A	A	A	A	A	A	A	A	A	A	A	5.26^{ab}	5.10^{a-d}	6.80^{a-c}	6.59^{a-c}
4659	24		B	B	B	B	B	B	B	B	B	B	A	A	A	A	A	A	A	A	A	A	A	5.39^{ab}	--	--	--
4305	20	x	B	B	B	B	B	B	B	B	B	B	B	A	A	A	A	A	A	A	A	A	A	4.98^{ab}	4.70^{b-h}	6.40^{a-g}	6.36^{a-c}
7743	112		B	B	B	B	B	B	B	B	B	B	B	A	A	A	A	A	A	A	A	A	A	5.46^{ab}	--	--	--
7766	113	x	B	B	B	B	B	B	B	B	B	B	B	B	B	A	A	A	A	A	A	A	A	5.45^{ab}	4.97^{a-f}	6.40^{a-g}	6.89^{a}
7868	117	x	B	B	B	B	B	B	B	B	B	B	B	B	B	A	A	A	A	A	A	A	A	5.38^{ab}	4.77^{a-h}	6.70^{a-d}	5.88^{a-c}
4718	25	x	B	B	B	B	B	B	B	B	B	B	B	B	B	B	A	A	A	A	A	A	A	5.53^{a}	4.84^{a-g}	6.50^{a-f}	6.13^{a-c}
4759	26	x	B	B	B	B	B	B	B	B	B	B	B	B	B	B	A	A	A	A	A	A	A	5.67^{a}	4.90^{a-f}	6.70^{a-d}	6.23^{a-c}
5339	48	x	B	B	B	B	B	B	B	B	B	B	B	B	B	B	B	A	A	A	A	A	A	5.65^{a}	4.90^{a-f}	6.40^{a-g}	6.48^{a-c}
4969	37	x	B	B	B	B	B	B	B	B	B	B	B	B	B	B	B	B	B	A	A	A	A	5.22^{ab}	4.66^{c-h}	6.40^{a-g}	5.93^{a-c}
7799	114		B	B	B	B	B	B	B	B	B	B	B	B	B	B	B	B	B	A	A	A	A	4.74^{ab}	--	--	--

Table S3, cont.

Line Name	Genotype ID	2010	Genotype																					Means Separation			
			TG194	T0408	SSR67	J1	At22570	TG523	At16710	U340899	CT182	At22260	At02870	At44446	At21690	At44790	cLEX4G10	cLEB7LI	TG147	At14260	At10050	At04590	TG400	09FD_120 Loc1&2 LEAF	10FD-early_83 Loc1&2 LEAF	10FD-late_83 Loc1 LEAF	10FD-late_83 Loc2 LEAF
**3845**	**7**	x	B	B	B	B	B	B	B	B	B	B	B	B	B	B	B	B	B	B	B	A	A	5.33^{ab}	4.97^{a-f}	6.30^{a-g}	6.54^{a-c}
**4961**	**36**		B	B	B	B	B	B	B	B	B	B	B	B	B	B	B	B	B	B	B	A	A	4.76^{ab}	--	--	--
**7952**	**118**		B	B	B	B	B	B	B	B	B	B	B	B	B	B	B	B	B	B	B	A	A	5.01^{ab}	--	--	--
**5385**	**50**	x	B	B	B	B	B	B	B	B	B	B	B	B	B	B	B	B	B	B	B	B	A	5.37^{ab}	4.60^{c-h}	5.80^{d-g}	6.00^{a-c}
**5422**	**51**		B	B	B	B	B	B	B	B	B	B	B	B	B	B	B	B	B	B	B	B	A	4.81^{ab}	--	--	--
**4993**	**38**	x	A	B	B	B	B	B	B	B	B	B	B	B	B	B	B	B	B	B	B	B	B	5.24^{ab}	4.73^{b-h}	5.70^{e-g}	5.57^{bc}
**5010**	**39**	x	A	B	B	B	B	B	B	B	B	B	B	B	B	B	B	B	B	B	B	B	B	5.15^{ab}	4.65^{c-h}	6.20^{a-g}	5.88^{a-c}
**5015**	**40**		A	B	B	B	B	B	B	B	B	B	B	B	B	B	B	B	B	B	B	B	B	5.36^{ab}	--	--	--
**5055**	**41**	x	A	A	B	B	B	B	B	B	B	B	B	B	B	B	B	B	B	B	B	B	B	4.87^{ab}	4.68^{b-h}	6.00^{b-g}	5.57^{bc}
**4056**	**13**	x	A	A	A	B	B	B	B	B	B	B	B	B	B	B	B	B	B	B	B	B	B	5.50^{ab}	4.97^{a-f}	6.60^{a-e}	6.25^{a-c}
**4558**	**23**		A	A	A	B	B	B	B	B	B	B	B	B	B	B	B	B	B	B	B	B	B	5.28^{ab}	--	--	--
**4018**	**12**	x	A	A	A	A	A	A	B	B	B	B	B	B	B	B	B	B	B	B	B	B	B	4.88^{ab}	4.35^{f-h}	5.90^{c-g}	6.05^{a-c}
**4861**	**29**		A	A	A	A	A	A	B	B	B	B	B	B	B	B	B	B	B	B	B	B	B	4.63^{ab}	--	--	--
**5162**	**43**	x	A	A	A	A	A	A	A	B	B	B	B	B	B	B	B	B	B	B	B	B	B	5.15^{ab}	4.73^{b-h}	6.00^{b-g}	5.89^{a-c}
**7826**	**115**		A	A	A	A	A	A	A	B	B	B	B	B	B	B	B	B	B	B	B	B	B	5.08^{ab}	--	--	--
**4182**	**15**	x	A	A	A	A	A	A	A	A	B	B	B	B	B	B	B	B	B	B	B	B	B	4.83^{ab}	4.56^{c-h}	5.90^{c-g}	6.36^{a-c}
**5186**	**44**		A	A	A	A	A	A	A	A	B	B	B	B	B	B	B	B	B	B	B	B	B	4.92^{ab}	--	--	--
**8032**	**120**	x	A	A	A	A	A	A	A	A	A	B	B	B	B	B	B	B	B	B	B	B	B	**4.04^{b}**	4.36^{e-h}	5.70^{e-g}	6.00^{a-c}
**4442**	**21**	x	A	A	A	A	A	A	A	A	A	A	B	B	B	B	B	B	B	B	B	B	B	5.00^{ab}	5.00^{a-e}	6.00^{b-g}	6.13^{a-c}
**4922**	**32**		A	A	A	A	A	A	A	A	A	A	B	B	B	B	B	B	B	B	B	B	B	5.12^{ab}	--	--	--
**3688**	**1**	x	A	A	A	A	A	A	A	A	A	A	A	B	B	B	B	B	B	B	B	B	B	5.04^{ab}	4.52^{d-h}	5.80^{d-g}	6.00^{a-c}

Table S3, cont.

			Genotype																					Means Separation			
Line Name	Genotype ID	2010	TG194	T0408	SSR67	J1	At22570	TG523	At16710	U340899	CT182	At22260	At02870	At44446	At21690	At44790	cLEX4G10	cLEB7LI	TG147	At14260	At10050	At04590	TG400	09FD_120 Loc1&2 LEAF	10FD-early_83 Loc1&2 LEAF	10FD-late_83 Loc1 LEAF	10FD-late_83 Loc2 LEAF
**4106**	**14**	x	A	A	A	A	A	A	A	A	A	A	A	B	B	B	B	B	B	B	B	B	B	4.93^{ab}	4.16^{h}	5.90^{c-g}	6.00^{a-c}
**4925**	**33**	x	A	A	A	A	A	A	A	A	A	A	A	A	A	B	B	B	B	B	B	B	B	5.19^{ab}	4.93^{a-f}	6.90^{ab}	6.59^{a-c}
**4498**	**22**	x	A	A	A	A	A	A	A	A	A	A	A	A	A	A	B	B	B	B	B	B	B	4.92^{ab}	5.04^{a-d}	6.60^{a-e}	6.30^{a-c}
**3723**	**2**	x	A	A	A	A	A	A	A	A	A	A	A	A	A	A	B	B	B	B	B	B	B	4.88^{ab}	4.34^{f-h}	6.20^{a-g}	6.13^{a-c}
**4794**	**27**	x	A	A	A	A	A	A	A	A	A	A	A	A	A	A	B	B	B	B	B	B	B	5.03^{ab}	4.76^{a-h}	6.10^{a-g}	6.71^{ab}
**4802**	**28**	x	A	A	A	A	A	A	A	A	A	A	A	A	A	A	A	B	B	B	B	B	B	4.93^{ab}	5.03^{a-d}	6.70^{a-d}	5.95^{a-c}
**5245**	**46**	x	A	A	A	A	A	A	A	A	A	A	A	A	A	A	A	B	B	B	B	B	B	5.20^{ab}	4.76^{a-h}	6.30^{a-g}	6.18^{a-c}
**3740**	**3**	x	A	A	A	A	A	A	A	A	A	A	A	A	A	A	A	A	A	B	B	B	B	4.65^{ab}	4.51^{d-h}	6.50^{a-f}	6.30^{a-c}
**5285**	**47**	x	A	A	A	A	A	A	A	A	A	A	A	A	A	A	A	A	A	B	B	B	B	4.93^{ab}	4.73^{b-h}	6.40^{a-g}	6.43^{a-c}
**3765**	**4**	x	A	A	A	A	A	A	A	A	A	A	A	A	A	A	A	A	A	A	A	B	B	4.97^{ab}	4.48^{d-h}	6.30^{a-g}	6.05^{a-c}
**4897**	**31**	x	A	A	A	A	A	A	A	A	A	A	A	A	A	A	A	A	A	A	A	B	B	5.04^{ab}	4.47^{d-h}	<u>**5.50^{g}**</u>	5.82^{a-c}
**5362**	**49**	x	A	A	A	A	A	A	A	A	A	A	A	A	A	A	A	A	A	A	A	B	B	4.82^{ab}	4.82^{a-g}	6.50^{a-f}	6.07^{a-c}
**4877**	**30**	x	A	A	A	A	A	A	A	A	A	A	A	A	A	A	A	A	A	A	A	A	B	5.18^{ab}	4.82^{a-g}	7.00^{a}	6.54^{a-c}
**8009**	**119**	x	A	A	A	A	A	A	A	A	A	A	A	A	A	A	A	A	A	A	A	A	B	4.80^{ab}	4.69^{b-h}	6.60^{a-e}	6.36^{a-c}
**3814**	**5**		A	A	A	A	A	A	A	A	A	A	A	A	A	A	A	A	A	A	A	A	A	5.98^{a}	--	--	--
**Means Separation**																								*	*	*	*

Table S3, cont.

Line Name	Genotype ID	2010	Means Separation												
			GC_120 p7629 LEAF	GC_120 p10353 LEAF	GC_120 Sal10 LEAF	09FD_120 Loc1&2 STEM	10FD-early_83 Loc1 STEM	10FD-early_83 Loc2 STEM	10FD-late_83 Loc1 STEM	10FD-late_83 Loc2 STEM	GC_120 p7629 STEM	GC_120 p10353 STEM	GC-1_120 Sal10 STEM	GC-2_120 Sal10 STEM	GC-3_120 Sal10 STEM
**Hyp45-A**	**121**	x	3.75^{a}	4.45^{a}	4.08	3.38^{ab}	3.78^{b-e}	3.02^{a-d}	4.90^{a}	4.79^{ab}	2.26^{a}	2.45	2.03	2.45	1.77^{ab}
**Hyp45-B**	**122**		--	--	--	3.37^{a-c}	--	--	--	--	--	--	--	--	--
**E6203-A**	**123**	x	--	--	--	2.61^{bc}	3.68^{c-e}	2.70^{b-d}	4.69^{ab}	4.53^{a-d}	--	--	--	--	--
**E6203-B**	**124**	x	3.41^{ab}	4.25^{a-c}	3.87	2.64^{bc}	3.78^{b-e}	2.70^{b-d}	4.56^{ab}	4.18^{a-d}	1.80^{ab}	2.19	1.76	2.22	1.70^{ab}
**E6203-C**	**129**	x	--	--	--	--	3.64^{c-e}	2.75^{b-d}	4.71^{ab}	4.62^{a-d}	--	--	--	--	--
**E6203-D**	**130**	x	--	--	--	--	3.56^{c-e}	2.38^{de}	4.47^{ab}	4.33^{a-d}	--	--	--	--	--
**LB11-NIL-A**	**126**	x	3.15^{b}	3.55^{bc}	3.47	2.75^{a-c}	3.64^{c-e}	2.66^{b-d}	4.21^{ab}	3.88^{b-d}	1.34^{b}	1.77	1.49	2.25	1.66^{b}
**LB11-NIL-B**	**128**	x	--	--	--	--	3.62^{c-e}	2.50^{cd}	**4.07^{b}**	3.79^{cd}	--	--	--	--	--
**3951**	**9**	x	3.83^{ab}	4.80^{a-c}	4.29	2.95^{a-c}	3.59^{c-e}	2.85^{a-d}	4.26^{ab}	3.83^{cd}	1.93^{ab}	2.42	2.39	3.05	1.68^{ab}
**5103**	**42**		3.25^{ab}	3.43^{a-c}	4.62	**2.33^{c}**	--	--	--	--	1.53^{ab}	1.47	2.42	2.84	1.93^{ab}
**4228**	**16**	x	3.00^{ab}	3.75^{a-c}	4.10	2.84^{a-c}	3.41^{c-e}	**1.27^{e}**	4.36^{ab}	3.96^{b-d}	1.90^{ab}	1.79	1.44	3.81	0.86^{b}
**3999**	**11**	x	3.29^{ab}	4.48^{a-c}	4.33	2.90^{a-c}	**3.26^{e}**	2.28^{de}	4.28^{ab}	3.91^{b-d}	1.69^{ab}	2.34	1.53	2.01	1.95^{ab}
**4945**	**35**		3.64^{ab}	4.12^{a-c}	4.16	2.92^{a-c}	--	--	--	--	1.31^{ab}	2.10	2.19	2.25	1.41^{ab}
**3892**	**8**	x	3.21^{ab}	4.43^{a-c}	4.55	2.69^{bc}	3.53^{c-e}	2.10^{de}	4.56^{ab}	4.11^{a-d}	1.70^{ab}	2.41	2.34	2.74	1.78^{ab}

Table S3, cont.

Line Name	Genotype ID	2010	GC_120 p7629 LEAF	GC_120 p10353 LEAF	GC_120 Sal10 LEAF	09FD_120 Loc1&2 STEM	10FD-early_83 Loc1 STEM	10FD-early_83 Loc2 STEM	10FD-late_83 Loc1 STEM	10FD-late_83 Loc2 STEM	GC_120 p7629 STEM	GC_120 p10353 STEM	GC-1_120 Sal10 STEM	GC-2_120 Sal10 STEM	GC-3_120 Sal10 STEM
			Means Separation												
**3969**	**10**	x	3.59^{ab}	3.88^{a-c}	3.44	3.55^{ab}	3.70^{c-e}	2.48^{cd}	4.69^{ab}	4.46^{a-d}	2.14^{ab}	1.77	1.02	2.09	2.32^{ab}
**4257**	**17**		3.62^{ab}	4.43^{a-c}	4.77	3.22^{a-c}	--	--	--	--	2.01^{ab}	2.42	2.83	3.25	2.19^{ab}
**4940**	**34**		3.53^{ab}	4.06^{a-c}	4.15	2.59^{bc}	--	--	--	--	1.55^{ab}	2.39	1.81	2.49	2.32^{ab}
**7717**	**110**	x	3.66^{ab}	3.90^{a-c}	3.38	3.26^{a-c}	3.62^{c-e}	2.77^{b-d}	4.71^{ab}	4.70^{a-c}	2.30^{ac}	2.18	1.61	1.89	2.28^{ab}
**7731**	**111**		3.66^{ab}	4.35^{a-c}	3.90	3.31^{a-c}	--	--	--	--	1.96^{ab}	2.88	1.44	2.29	1.85^{ab}
**4265**	**18**	x	3.26^{ab}	4.05^{a-c}	3.88	3.21^{a-c}	3.59^{c-e}	2.80^{b-d}	4.68^{ab}	4.79^{ab}	1.67^{ab}	2.09	1.82	2.59	1.14^{ab}
**4290**	**19**		3.21^{ab}	4.00^{a-c}	4.53	2.53^{bc}	--	--	--	--	1.62^{ab}	2.02	1.51	3.33	2.25^{ab}
**5224**	**45**		3.30^{ab}	4.24^{a-c}	3.75	2.60^{bc}	--	--	--	--	1.70^{ab}	2.10	1.93	3.29	1.34^{ab}
**7852**	**116**	x	2.94^{ab}	3.55^{a-c}	3.22	3.40^{ab}	3.94^{a-c}	2.70^{b-d}	4.40^{ab}	3.78^{cd}	1.43^{ab}	1.91	0.90	1.49	1.01^{b}
**3827**	**6**	x	3.90^{ab}	4.17^{a-c}	4.57	3.02^{a-c}	3.69^{c-e}	3.02^{a-d}	4.85^{a}	4.79^{ab}	2.07^{ab}	2.29	2.13	3.39	2.25^{ab}
**4659**	**24**		3.20^{ab}	**3.00^{c}**	3.49	2.91^{a-c}	--	--	--	--	1.83^{ab}	**1.20**	2.41	2.86	0.79^{b}
**4305**	**20**	x	3.48^{ab}	4.16^{a-c}	4.58	2.56^{bc}	3.78^{b-e}	2.85^{a-d}	4.52^{ab}	4.13^{a-d}	2.00^{ab}	2.11	2.48	3.78	2.52^{ab}
**7743**	**112**		3.61^{ab}	4.04^{a-c}	4.98	2.98^{a-c}	--	--	--	--	1.97^{ab}	2.22	1.75	2.44	2.32^{ab}
**7766**	**113**	x	3.88^{ab}	4.04^{a-c}	3.73	2.88^{a-c}	3.59^{c-e}	2.30^{de}	4.74^{ab}	4.62^{a-d}	2.18^{ab}	2.12	2.29	2.17	**0.55^{b}**
**7868**	**117**	x	3.27^{ab}	4.03^{a-c}	3.02	2.64^{bc}	3.52^{c-e}	2.63^{cd}	4.41^{ab}	3.86^{b-d}	1.87^{ab}	2.63	0.87	1.58	1.95^{ab}
**4718**	**25**	x	3.26^{ab}	3.79^{a-c}	4.48	2.76^{a-c}	3.71^{c-e}	2.60^{cd}	4.66^{ab}	4.20^{a-d}	1.33^{ab}	1.88	1.54	2.94	1.26^{ab}
**4759**	**26**	x	3.48^{ab}	3.35^{a-c}	2.97	3.41^{ab}	3.59^{c-e}	2.92^{a-d}	4.54^{ab}	4.27^{a-d}	2.14^{ab}	2.03	1.44	1.37	1.04^{ab}
**5339**	**48**	x	3.17^{ab}	4.30^{a-c}	3.53	2.92^{a-c}	3.68^{c-e}	2.55^{cd}	4.60^{ab}	4.34^{a-d}	1.48^{ab}	2.16	1.32	3.48	1.28^{ab}
**4969**	**37**	x	3.02^{ab}	3.30^{a-c}	3.62	3.04^{a-c}	3.36^{de}	2.98^{a-d}	4.58^{ab}	4.29^{a-d}	1.51^{ab}	1.65	1.15	2.36	0.63^{b}
**7799**	**114**		3.31^{ab}	3.83^{a-c}	3.58	3.16^{a-c}	--	--	--	--	1.60^{ab}	2.09	2.34	2.12	0.88^{b}

Table S3, cont.

			Means Separation												
**Line Name**	**Genotype ID**	**2010**	**GC_120 p7629 LEAF**	**GC_120 p10353 LEAF**	**GC_120 Sal10 LEAF**	**09FD_120 Loc1&2 STEM**	**10FD-early_83 Loc1 STEM**	**10FD-early_83 Loc2 STEM**	**10FD-late_83 Loc1 STEM**	**10FD-late_83 Loc2 STEM**	**GC_120 p7629 STEM**	**GC_120 p10353 STEM**	**GC-1_120 Sal10 STEM**	**GC-2_120 Sal10 STEM**	**GC-3_120 Sal10 STEM**
**3845**	**7**	x	3.23^{ab}	3.94^{a-c}	3.48	3.31^{a-c}	3.74^{c-e}	2.95^{a-d}	4.71^{ab}	4.55^{a-d}	1.50^{ab}	2.10	1.81	1.92	2.14^{ab}
**4961**	**36**		3.18^{ab}	4.14^{a-c}	4.17	2.96^{a-c}	--	--	--	--	1.68^{ab}	2.32	3.36	1.72	1.78^{ab}
**7952**	**118**		3.14^{ab}	4.00^{a-c}	3.41	3.25^{a-c}	--	--	--	--	1.97^{ab}	1.94	2.70	**0.91**	2.28^{ab}
**5385**	**50**	x	2.96^{ab}	4.15^{a-c}	4.06	3.16^{a-c}	3.64^{c-e}	2.65^{b-d}	4.38^{ab}	3.94^{b-d}	1.36^{ab}	2.58	1.85	3.70	1.05^{ab}
**5422**	**51**		3.46^{ab}	4.21^{a-c}	4.37	2.80^{a-c}	--	--	--	--	2.04^{ab}	2.14	2.55	3.45	2.42^{ab}
**4993**	**38**	x	3.19^{ab}	3.93^{a-c}	3.61	3.47^{ab}	3.82^{b-e}	3.82^{ab}	4.74^{ab}	4.71^{a-c}	2.01^{ab}	2.84	2.43	3.33	2.06^{ab}
**5010**	**39**	x	3.64^{ab}	3.67^{a-c}	3.67	2.87^{a-c}	3.64^{c-e}	2.80^{b-d}	4.52^{ab}	4.17^{a-d}	1.30^{ab}	1.20	1.90	1.36	1.60^{ab}
**5015**	**40**		3.59^{ab}	4.94^{ab}	4.09	2.82^{a-c}	--	--	--	--	1.42^{ab}	2.60	1.78	1.90	2.28^{ab}
**5055**	**41**	x	3.31^{ab}	4.03^{a-c}	3.74	3.21^{a-c}	3.78^{b-e}	2.83^{b-d}	4.46^{ab}	3.96^{b-d}	1.64^{ab}	2.43	1.03	2.96	2.17^{ab}
**4056**	**13**	x	3.52^{ab}	4.43^{a-c}	3.88	3.74^{a}	4.42^{a}	4.02^{a}	4.98^{a}	4.95^{a}	1.97^{ab}	2.40	1.89	2.75	2.85^{ab}
**4558**	**23**		3.37^{ab}	4.33^{a-c}	3.91	3.38^{ab}	--	--	--	--	1.88^{ab}	2.32	3.82	2.36	1.08^{ab}
**4018**	**12**	x	3.50^{ab}	4.65^{a-c}	4.14	2.83^{a-c}	3.80^{b-e}	2.95^{a-d}	4.41^{ab}	4.25^{a-d}	1.79^{ab}	2.26	0.94	4.19	2.29^{ab}
**4861**	**29**		3.12^{ab}	3.46^{a-c}	4.24	2.98^{a-c}	--	--	--	--	1.80^{ab}	1.64	1.48	1.89	2.48^{ab}
**5162**	**43**	x	3.67^{ab}	4.09^{a-c}	3.49	3.41^{ab}	4.32^{ab}	3.65^{a-c}	4.71^{ab}	4.52^{a-d}	1.94^{ab}	2.51	2.53	1.47	2.45^{ab}
**7826**	**115**		3.25^{ab}	4.05^{a-c}	3.67	3.52^{ab}	--	--	--	--	1.28^{ab}	2.19	0.83	1.87	2.95^{ab}
**4182**	**15**	x	3.61^{ab}	4.06^{a-c}	4.13	2.93^{a-c}	3.78^{b-e}	2.83^{b-d}	4.40^{ab}	4.15^{a-d}	2.28^{ab}	2.31	2.18	3.13	1.65^{ab}
**5186**	**44**		3.59^{ab}	4.51^{a-c}	4.19	3.22^{a-c}	--	--	--	--	2.07^{ab}	2.80	1.64	3.61	1.79^{ab}
**8032**	**120**	x	3.11^{ab}	3.77^{a-c}	3.05	3.00^{a-c}	3.60^{c-e}	2.05^{de}	4.29^{ab}	3.88^{b-d}	1.54^{ab}	1.85	2.69	1.87	2.33^{ab}
**4442**	**21**	x	3.71^{ab}	4.51^{a-c}	3.8	3.18^{a-c}	3.76^{b-e}	3.07^{a-d}	4.60^{ab}	4.43^{a-d}	2.21^{ab}	2.22	1.14	3.05	3.20^{ab}
**4922**	**32**		3.67^{ab}	4.59^{a-c}	3.63	2.71^{bc}	--	--	--	--	2.04^{ab}	2.25	1.94	2.62	2.22^{ab}
**3688**	**1**	x	3.14^{ab}	4.15^{a-c}	4.46	2.75^{a-c}	3.38^{c-e}	2.73^{b-d}	4.33^{ab}	4.02^{a-d}	$\mathbf{1.25}^{bc}$	2.60	2.88	2.32	3.08^{ab}

Table S3, cont.

Line Name	Genotype ID	2010	Means Separation												
			GC_120 p7629 LEAF	GC_120 p10353 LEAF	GC_120 Sal10 LEAF	09FD_120 Loc1&2 STEM	10FD-early_83 Loc1 STEM	10FD-early_83 Loc2 STEM	10FD-late_83 Loc1 STEM	10FD-late_83 Loc2 STEM	GC_120 p7629 STEM	GC_120 p10353 STEM	GC-1_120 Sal10 STEM	GC-2_120 Sal10 STEM	GC-3_120 Sal10 STEM
**4106**	**14**	x	**<u>2.72</u>**^{b}	3.18^{a-c}	3.00	2.73^{a-c}	3.62^{c-e}	2.55^{cd}	4.35^{ab}	3.93^{b-d}	1.29^{bc}	1.33	1.88	2.54	1.44^{ab}
**4925**	**33**	x	3.44^{ab}	4.34^{a-c}	4.06	2.72^{a-c}	3.64^{c-e}	2.95^{a-d}	4.71^{ab}	4.46^{a-d}	1.84^{ab}	2.45	1.92	3.83	1.66^{ab}
**4498**	**22**	x	2.99^{ab}	3.16^{a-c}	3.38	3.16^{a-c}	3.72^{c-e}	2.17^{de}	4.58^{ab}	4.02^{a-d}	1.46^{ab}	1.72	0.62	2.50	1.62^{ab}
**3723**	**2**	x	3.44^{ab}	4.53^{a-c}	3.85	2.87^{a-c}	3.58^{c-e}	2.32^{de}	4.21^{ab}	3.73^{d}	1.78^{ab}	2.70	**<u>0.54</u>**	3.11	2.20^{ab}
**4794**	**27**	x	3.18^{ab}	4.13^{a-c}	4.49	2.84^{a-c}	3.39^{c-e}	2.42^{de}	4.52^{ab}	4.37^{a-d}	1.43^{ab}	2.67	1.69	3.36	2.73^{ab}
**4802**	**28**	x	3.66^{ab}	3.95^{a-c}	4.52	3.37^{ab}	3.90^{a-d}	2.95^{a-d}	4.63^{ab}	4.36^{a-d}	2.34^{ab}	2.59	1.92	3.12	3.71^{a}
**5245**	**46**	x	3.54^{ab}	4.47^{a-c}	3.83	2.95^{a-c}	3.48^{c-e}	2.37^{de}	4.27^{ab}	3.89^{b-d}	2.17^{ab}	2.07	2.57	2.06	1.97^{ab}
**3740**	**3**	x	3.58^{ab}	4.00^{a-c}	3.77	3.00^{a-c}	3.44^{c-e}	2.08^{de}	4.43^{ab}	**<u>3.70</u>**^{d}	2.05^{ab}	2.11	1.94	2.59	2.85^{ab}
**5285**	**47**	x	3.39^{ab}	3.99^{a-c}	4.23	3.27^{a-c}	3.84^{b-d}	3.15^{a-d}	4.60^{ab}	4.32^{a-d}	2.31^{ab}	2.43	1.73	2.89	3.12^{ab}
**3765**	**4**	x	3.42^{ab}	4.08^{a-c}	4.16	3.09^{a-c}	3.76^{b-e}	3.09^{a-d}	4.52^{ab}	4.12^{a-d}	2.05^{ab}	2.20	3.35	3.11	0.72^{b}
**4897**	**31**	x	3.42^{ab}	3.80^{a-c}	4.15	3.17^{a-c}	3.74^{c-e}	3.23^{a-d}	4.32^{ab}	3.98^{b-d}	1.98^{ab}	2.12	1.45	2.18	2.35^{ab}
**5362**	**49**	x	3.70^{ab}	4.02^{a-c}	3.86	3.27^{a-c}	3.66^{c-e}	2.93^{a-d}	4.43^{ab}	3.88^{b-d}	2.31^{ab}	2.12	2.42	3.60	1.03^{b}
**4877**	**30**	x	3.82^{ab}	4.12^{a-c}	3.74	2.93^{a-c}	3.79^{b-e}	2.53^{cd}	4.78^{ab}	4.53^{a-d}	2.05^{ab}	2.46	2.58	2.55	1.20^{ab}
**8009**	**119**	x	3.28^{ab}	4.55^{a-c}	3.72	3.25^{a-c}	3.64^{c-e}	2.78^{b-d}	4.49^{ab}	4.27^{a-d}	1.55^{ab}	2.94	1.47	3.62	2.14^{ab}
**3814**	**5**		3.53^{ab}	3.79^{a-c}	4.31	2.97^{a-c}	--	--	--	--	1.76^{ab}	1.54	1.93	3.18	1.81^{ab}
**Means Separation**			*	*	ns	*	*	*	*	*	*	ns	ns	ns	*

[a] In Proc MIXED in SAS, means separation are calculated using both the difference between means and the number of replications for each entry. Therefore, letter rankings are not always contiguous.

A double dash (--) indicates that the line was not included in the experiment.

An asterisk (*) indicates significance at $P \leq 0.05$ and “ns” indicates not significant.
